# Impacts of ocean warming on echinoderms: A meta‐analysis

**DOI:** 10.1002/ece3.10307

**Published:** 2023-08-08

**Authors:** Bethan J. Lang, Jennifer M. Donelson, Kevin R. Bairos‐Novak, Carolyn R. Wheeler, Ciemon F. Caballes, Sven Uthicke, Morgan S. Pratchett

**Affiliations:** ^1^ Australian Research Council Centre of Excellence for Coral Reef Studies James Cook University Townsville Queensland Australia; ^2^ AIMS@JCU James Cook University Townsville Queensland Australia; ^3^ School for the Environment The University of Massachusetts Boston Boston Massachusetts USA; ^4^ National Science Foundation EPSCoR—Guam Ecosystems Collaboratorium for Corals and Oceans University of Guam Marine Laboratory Mangilao Guam USA; ^5^ Australian Institute of Marine Science Townsville Queensland Australia

**Keywords:** brittle stars, climate change, environmental change, global ecology, life stage, region, sea cucumbers, sea stars, sea urchins, temperature

## Abstract

Rising ocean temperatures are threatening marine species and populations worldwide, and ectothermic taxa are particularly vulnerable. Echinoderms are an ecologically important phylum of marine ectotherms and shifts in their population dynamics can have profound impacts on the marine environment. The effects of warming on echinoderms are highly variable across controlled laboratory‐based studies. Accordingly, synthesis of these studies will facilitate the better understanding of broad patterns in responses of echinoderms to ocean warming. Herein, a meta‐analysis incorporating the results of 85 studies (710 individual responses) is presented, exploring the effects of warming on various performance predictors. The mean responses of echinoderms to all magnitudes of warming were compared across multiple biological responses, ontogenetic life stages, taxonomic classes, and regions, facilitated by multivariate linear mixed effects models. Further models were conducted, which only incorporated responses to warming greater than the projected end‐of‐century mean annual temperatures at the collection sites. This meta‐analysis provides evidence that ocean warming will generally accelerate metabolic rate (+32%) and reduce survival (−35%) in echinoderms, and echinoderms from subtropical (−9%) and tropical (−8%) regions will be the most vulnerable. The relatively high vulnerability of echinoderm larvae to warming (−20%) indicates that this life stage may be a significant developmental bottleneck in the near‐future, likely reducing successful recruitment into populations. Furthermore, asteroids appear to be the class of echinoderms that are most negatively affected by elevated temperature (−30%). When considering only responses to magnitudes of warming representative of end‐of‐century climate change projections, the negative impacts on asteroids, tropical species and juveniles were exacerbated (−51%, −34% and −40% respectively). The results of these analyses enable better predictions of how keystone and invasive echinoderm species may perform in a warmer ocean, and the possible consequences for populations, communities and ecosystems.

## Graphical abstract

This meta‐analysis on the effects of ocean warming on echinoderms reveals that survival rates are substantially reduced. Echinoderms from regions closer to the equator appear to be the most vulnerable to elevated temperatures, and larvae may face significant developmental challenges that could hinder successful population recruitment. Among echinoderm classes, starfish were found to be particularly vulnerable, and when considering end‐of‐century climate change projections, the negative impacts on starfish and tropical echinoderms are exacerbated.

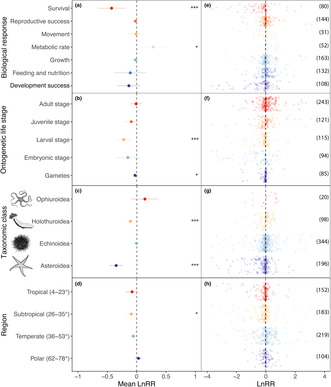

## 1 INTRODUCTION

Ocean warming, a repercussion of anthropogenic climate change, is one of the principal emerging threats facing marine ecosystems (IPCC, 2019). An increased prevalence and intensity of marine heatwaves (i.e. warming above the 90th percentile for at least five consecutive days; Hobday et al., 2016) in recent years has already resulted in the degradation of some of the world's most vulnerable marine ecosystems, such as coral reefs, sea grass beds and kelp forests (Filbee‐Dexter et al., 2020; Mellin et al., 2019; Strydom et al., 2020). Within these ecosystems, ectotherms are particularly thermosensitive because their body temperature, and therefore their physiology and behaviour, are naturally linked to their thermal environment (Lagerspetz & Vainio, 2006). Accordingly, ocean warming has been implicated in population declines, phenological shifts and range shifts in an array of marine ectotherms (Poloczanska et al., 2016). Considering the ecological and economic importance of many marine ectotherms (Li et al., 2011; Ling et al., 2009; Munday et al., 2013), a knowledge of how elevated temperatures will impact these organisms is essential.

Due to the widespread threat of ocean warming, it is important to understand how common biological and ecological aspects shape the responses of marine ectotherms to a warming environment. For example, certain biological responses (e.g. metabolic activity and survival rates) and life stages may have differing sensitivities to elevated temperatures (Dahlke et al., 2020; Pereira Santos et al., 2021; Sampaio et al., 2021). Biological responses associated with physiology are likely to be particularly sensitive to environmental warming in ectotherms, with their magnitude and direction dependent upon where the temperatures experienced fall within the organisms' thermal window (Pörtner et al., 2017; Sampaio et al., 2021). Physiological performance is often assessed nonlethally via oxygen consumption rate measurements, which serves as a proxy for aerobic metabolism, and evidence suggests that this measure can operate as a good proxy for other aspects of performance (Pörtner et al., 2017; Svendsen et al., 2016). In general, aerobic metabolism is only able to increase to the organisms' thermal limit, after which, constraints on oxygen delivery will lead to metabolic depression and declines in energy production (Lang et al., 2021; Pörtner et al., 2017). The additional aerobic metabolic costs of warming may impact growth, development, activity, feeding and reproduction (Pörtner et al., 2017; Schulte, 2015). Reproduction is typically sensitive to elevated temperatures, and resulting embryos and larvae are often highly vulnerable to warming (Byrne, 2011; Collin et al., 2021; Dahlke et al., 2020), and other concurrent stressors, such as ocean acidification (Byrne, 2011; Kroeker et al., 2010; Pandori & Sorte, 2018; Przeslawski et al., 2015). On the contrary, juveniles and adults are usually more thermotolerant, testament to their well‐developed physiological capacity and greater ability to modify their behaviour to withstand warmer conditions (Byrne, 2011; Dahlke et al., 2020; Lang et al., 2021; Nguyen et al., 2011; Sampaio et al., 2021). Behavioural plasticity and thermoregulation could allow marine ectotherms to buffer their experience of stressful thermal conditions and potentially reduce the costs of increased metabolic demands in warmer oceans (Wong & Candolin, 2015).

Responses of marine ectotherms to warming may be in part, a reflection of their evolutionary and thermal history. Responses to elevated temperatures in marine ectotherms have been demonstrated to vary taxonomically (e.g. Nguyen et al., 2011; Sampaio et al., 2021). These patterns are due to taxa having differing capacities to adjust their physiology and behaviour in response to warming, which may be associated with their phylogeny and thermal experience throughout evolutionary time (Bennett et al., 2021; Nguyen et al., 2011; Sunday et al., 2011). Tolerance to elevated temperatures is often attributed to the amount of thermal variation experienced (Hughes et al., 2018; Sunday et al., 2011). Seasonal thermal variability is low at tropical latitudes (Hughes et al., 2018; Madeira et al., 2017; Sunday et al., 2011). Consequently, marine ectotherms living in these relatively stable thermal environments often have a narrow thermal breadth, and live closer to their upper thermal limits compared with those inhabiting more variable thermal environments (Poloczanska et al., 2016; Pinsky et al., 2019; Schulte et al., 2011; Sherman, 2015; Sunday et al., 2011; Woolsey et al., 2015). Marine ectotherms exposed to greater seasonal thermal variability at temperate latitudes for instance often have wider thermal windows and have evolved greater capacity to withstand periods of thermal extremes via enhanced physiological and behavioural thermoregulation (Madeira et al., 2017; Sokolova & Pörtner, 2003; Sunday et al., 2011; Woolsey et al., 2015). This geographic pattern in vulnerability to warming may not always hold true, however, because vulnerability may also be dependent on other spatial factors, including the breadth of the geographic range, the part of the range in which they occur, their depth and habitat (Drake et al., 2017; O'Connor et al., 2012; Pey et al., 2011; Poloczanska et al., 2016; Sasaki et al., 2022; Zettlemoyer & Peterson, 2021).

The phylum Echinodermata, consisting of ~7000 extant species, including asteroids, echinoids, holothuroids and ophiuroids are an ecologically and economically important taxon in all oceans (Byrne & O'Hara, 2017). The ecological importance of echinoderms is partly linked to their ‘boom and bust’ population dynamics, and the remarkable beneficial or detrimental effects that these changes can have on marine ecosystems (Byrne & O'Hara, 2017; Lessios, 1988; Ling, 2013; Mellin et al., 2019; Menge et al., 2016; Uthicke et al., 2009). Population irruptions of crown‐of‐thorns starfish (*Acanthaster* cf. *solaris*), for instance, have resulted in significant coral loss on Australia's Great Barrier Reef (Mellin et al., 2019), while shifts in the distribution of large populations of the echinoid *Centrostephanus rodgersii* have contributed to the widespread and accelerated loss of highly diverse kelp forest ecosystems in Tasmania (Ling, 2013). Ocean warming is considered to be the primary cause of the distributional shift of *C. rodgersii* (Ling et al., 2009). Ocean warming has also been implicated in increasing the incidence and severity of disease outbreaks in a variety of echinoderm species (Clemente et al., 2014; Dungan et al., 1982; Lester et al., 2007; Menge et al., 2016). For instance, the asteroid *Pisaster ochraceus*, the first species to be coined a ‘keystone’ species (Paine, 1966; Wagner, 2010), helps maintain invertebrate diversity by providing top‐down control on mussel populations along the Pacific coast of North America, but has recently experienced significant population declines due to Sea Star Wasting Disease, which may be linked to ocean warming (Menge et al., 2016). Considering the numerous ecosystem impacts resulting from a ‘boom’ or ‘bust’ in echinoderm populations (Menge et al., 2016; Uthicke et al., 2009), there is a strong impetus to understand how echinoderm species may be impacted by a changing climate.

Despite an increasing number of controlled laboratory‐based studies focussing on understanding the effects of elevated temperatures on echinoderm species, there is little synthesis of the overarching impacts of warming, or explanations for the variation in vulnerability to elevated temperature observed within this phylum. Meta‐analyses have provided a useful statistical tool for quantitatively assessing broad patterns in the responses of marine ectotherms such as fish, molluscs and crustaceans to environmental change, including ocean warming, ocean acidification, hypoxia and reduced salinity (e.g. Hu et al., 2022; Kroeker et al., 2010; Pandori & Sorte, 2018; Pereira Santos et al., 2021; Przeslawski et al., 2015; Sampaio et al., 2021). To the authors' knowledge, there are no comparable meta‐analyses to date that have focussed on understanding whether echinoderms adhere to general biological, ecological or evolutionary expectations regarding their vulnerability to ocean warming (Byrne, 2011; Dahlke et al., 2020; Poloczanska et al., 2016; Sampaio et al., 2021; Sunday et al., 2011). Herein, a meta‐analysis using a comprehensive empirical dataset (see File S1 for the full dataset) is presented, consisting of information from 85 studies that explore the effect of warming on echinoderms. Specifically, the direction and magnitude of the effect of warming (relative to control temperatures) on performance was investigated, and how this varies depending on the (1) biological response tested, (2) ontogenetic life stage, (3) taxonomic class, (4) region and (5) experimental design. To further explore the potential effect of future ocean warming, a subset of the data was examined in which responses were only included if the experimental temperature was above the predicted end‐of‐century mean annual temperature (MAT; IPCC, 2019) at the location of collection. The outcomes of this meta‐analysis will aid in assessing the vulnerability of key echinoderm species to current and future ocean warming, and the broader impacts on the marine environment.

## 2 METHODS

### 2.1 Literature search

Relevant publications on the effect of warming on echinoderms were initially identified using systematic searches within ISI Web of Science (studies published before June 2021). The keywords used were: ‘temp*’, ‘thermal’, ‘thermotolerance’, ‘warm*’ and ‘climate change’ (an asterisk represents the wildcard operator in Web of Science, used to find different endings of keywords). These words were combined (using the ‘AND’ boolean) with both the common and/or Latin names of the five classes of echinoderms, that is, Asteroidea (starfish/sea stars), Echinoidea (sea urchins and sand dollars), Ophiuroidea (brittle stars), Holothuroidea (sea cucumbers) and Crinoidea (feather stars and sea lilies). The latter class was not included in this review due to a lack of relevant studies. The field tag used was ‘TS = Topic’. To avoid any bias associated with conducting literature searches in a single database (Martín‐Martín et al., 2018), systematic searches using ISI Web of Science were supplemented using Google Scholar and by exploring studies referenced in relevant publications. There was no limit on the year of publication for included studies, but field‐based studies were excluded, due to limited ability to control for other factors that may influence responses.

### 2.2 Data collection and selection

The papers that met these initial criteria (*n* = 143) were further screened for eligibility (see Appendix 1 for a flow diagram of the selection process). Studies were only incorporated if the responses fell under one of the following key biological responses: developmental success, feeding and nutrition, growth, metabolic rate (routine or resting), movement, reproductive success and survival (*n* = 3 studies that did not fall under the aforementioned biological responses were therefore excluded). Acute ‘ramping’ (i.e. warming rate > 2°C h^−1^) studies were not included due to the associated cumulative stress likely causing confounding deleterious effects on organism responses to warming (*n* = 5 studies excluded). Studies and individual responses were excluded if the control temperature was not specified or clearly stated (*n* = 21 studies excluded), or if the mean response for a control and at least one experimental temperature were not reported (*n* = 4 studies excluded). Finally, studies were excluded if the standard deviation or sufficient information for it to be calculated/estimated (i.e. standard error, upper and lower 95% confidence limits, interquartile range, minimum and maximum values) and/or the sample sizes were not provided (*n* = 22 studies excluded). If multiple synonymous responses were included in the publication (e.g. gonad wet weight and gonad dry weight), only one was included in the analyses. The responses at all elevated temperatures were included regardless of how realistic these temperatures were in the context of near‐future climate change. If a response was measured at multiple time points, in general the response at the first and last time point were used in the analyses, to avoid pseudoreplication, and taxonomic biases. If the first time point was immediately after environmental conditions were changed, the second and last time point were used instead (see Appendix 2 for a reference list of the data sources for the analyses).

For each response, the mean, standard deviation and number of replicates at both the control and experimental (elevated) temperatures were recorded. The control temperature was the temperature at which the individuals were habituated to prior to the experiments, or if there was no laboratory habituation period, then the temperature at the collection location was used. When raw or summary data tables were not published, but were presented in a graphical format, data were mined from the primary literature using the program WebPlotDigitizer (Automeris.io, 2021). The biological response being tested, ontogenetic life stage, the taxonomic class of the test subjects and the region were also recorded (see Appendix 3 for a table of these predictors and the groups within them). An attempt was made to gather data on the collection depth, and the latitudinal range of each species (to consider how range size, and the position of the individuals within the range, may impact vulnerability to warming), however, insufficient data were available (Living Australia, 2021). Experimental variables were recorded or calculated for each study, namely the habituation time at ambient temperature prior to experiments (days), the warming rate from the control to the experimental temperature (°C h^−1^), the exposure time at experimental temperatures (days) and the natural logarithm of the ratio of the experimental and control temperatures (*LnSR*; Pereira Santos et al., 2021; Sampaio et al., 2021).

The resulting values for *LnSR* were dependent not only on the difference between the temperature values, but also depended upon how hot or cold the control values were themselves; thus, the temperatures were adjusted accordingly. As in Pereira Santos et al. (2021) and Sampaio et al. (2021), all control temperatures were set to 2°C, and new experimental temperatures were calculated as the original experimental temperature minus the original control temperature, plus the new baseline control temperature. The mean annual temperature (MAT) for each study location was also established by extracting COBE long‐term mean sea surface temperature estimates from the National Oceanic and Atmospheric Administration (NOAA Physical Sciences Laboratory, 2021) using the coordinates of the collection locations (or approximate coordinates if not available). For each study, the temperature data from the 10 years prior to the year of the experiment were used to establish the MAT.

### 2.3 Effect size and variance calculation

Effect sizes, that is the ln‐transformed response ratios (*LnRR*), were calculated using the equation outlined in Hedges et al. (1999):

$$\text{LnRR}=\ln\left(\frac{{\bar{X}}_{E}}{{\bar{X}}_{C}}\right)$$

where ${\bar{X}}_{E}$ and ${\bar{X}}_{C}$ are the mean responses in the experimental and control treatments, respectively. The natural logarithm linearises these values, causing the values of both the experimental and control means to be weighted equally, and removes some of the skewness of the sampling distribution (Hedges et al., 1999). A positive *LnRR* indicates that the response is positively impacted by elevated temperature, while a negative *LnRR* means that the response is negatively impacted. Furthermore, if the *LnRR* is zero, then there is no effect of elevated temperature on the response. If for a given response, a higher mean value indicated a more detrimental effect (i.e. per cent abnormality, per cent mortality, development time and righting time), the sign of the *LnRR* was reversed for a more intuitive visualisation. The variance (inverse‐variance weights) of each response was calculated using the equation of Hedges et al. (1999):

$$v=\frac{{\left(S_{E}\right)}^{2}}{n_{E}{\bar{X}}_{E}^{2}}+\frac{{\left(S_{C}\right)}^{2}}{n_{C}{\bar{X}}_{C}^{2}}$$

where, *S* is the standard deviation and *n* is the sample size. The variance enables the precision of the estimate to be established. Observations with a greater sample size and lower standard deviation are more heavily weighted, as they are a more precise estimate of the effect size (Kroeker et al., 2010).

### 2.4 Data analyses

Multivariate multilevel linear mixed effects models were conducted in R v.4.1.2 (R Core Team, 2021) and used to establish the mean effects of various predictor variables (see Appendix 3). Separate models were fitted for each predictor (i.e. biological response, life stage, taxonomic class, region, habituation time at the control temperature, warming rate, and exposure time at the experimental temperature), as there were insufficient data available to consider multiple predictors simultaneously. Responses for metabolic rate were only included in the analysis for the ‘biological response’ predictor, due to the uncertainty regarding whether positive or negative changes in the metabolic rate translate to beneficial or detrimental effects on organismal fitness (Hu et al., 2022; Pörtner et al., 2017; Schulte, 2015). All models were carried out using the *rma.mv* function from the *‘*metafor’ package in R (Viechtbauer, 2010). These models account for the hierarchical structure of meta‐analytic data and considers the variation both within and between studies, while also accounting for any nonindependence of effect sizes (Cheung, 2019; Jackson et al., 2011; Konstantopoulos, 2011). The formula for each of the models, adapted from Pereira Santos et al. (2021) and Sampaio et al. (2021), was as follows:

$$\begin{array}{c} \text{Model}\;=\;\mathrm{rma}.\mathrm{mv}\left(\mathrm{yi}=\text{LnRR},𝑣\;=\;\text{Variance},\;\text{mods}=∼\text{LnSR}:\text{Predictor}-1,\text{test}\;=\;{}^{“}t^{”},\text{random}\;=\;\text{list}\left(∼1|\text{Study}\;\text{number}/\text{Response}\;\text{number}\right)\;\text{method}=“\text{REML}”,\;\text{data}\;=\;\text{data}\right) \end{array}$$

The categorical predictors (i.e. biological response, life stage, taxonomic class and region), were interacted with *LnSR* (‘*LnSR*: Predictor – 1’), which allowed for varying slopes of *LnSR* on *LnRR* for each predictor group, with a fixed intercept of 0 as in previous studies (Sampaio et al., 2021). *LnSR* was not required in the experimental variable models, as all predictors were continuous. The variance (v) was included in all models to weight responses based on their precision, as well as the random effects structure. ‘Response number’ was the number allocated to all responses at the different experimental temperatures for a given measure (e.g. per cent fertilisation success, gonad index, wet weight and locomotion speed) within a single study (see Appendix 4 for a full list of measures included within each ‘biological response’ group). The random effect was nested within the study number (~1|Study number/Response number). In most cases, there was only a single study per publication; however, if the paper included independent experiments on multiple echinoderm species, these were included as multiple studies per publication. Restricted maximum likelihood was used to fit all models, and *t*‐statistic methods were implemented, which are more conservative than default *z*‐statistic methods (Knapp & Hartung, 2003). To account for any deviations from the assumptions of the model, such as heteroscedasticity, non‐normality, as well as nonindependence of effect sizes, cluster‐robust confidence intervals (clustered by ‘Study number’) were calculated using the *robust* function in the ‘metafor’ package (Cheung, 2019; Viechtbauer, 2010). The Robust Test of Moderators (*Q*_M_) was used to establish whether there was a significant overall difference between the effect sizes for the different groups within each predictor model. Considering that it is not possible to get a test for residual heterogeneity based on cluster robust methods, the Test for Residual Heterogeneity (*Q*_E_) from the original model was utilised.

Sensitivity analyses were conducted on all models; specifically, the presence of influential observations and pseuedoreplication were tested. The influence of the three most represented species: *Acanthaster* spp. (tropical asteroid; *n* = 110 data points), *Apostichopus japonicus* (temperate holothuroid; *n* = 78 data points) and *Strongylocentrotus droebachiensis* (temperate and polar echinoid; *n* = 53 data points) were also tested in the taxonomic class and region models and were found not to substantially drive the results (see Appendix 5 for a table detailing the number of data points for each species of echinoderm included in the analyses, and Appendix 6.1 for model results). Furthermore, tests for publication bias were conducted, because studies are more likely to be published if their results are significant (Duval & Tweedie, 2000). Lastly, tests were conducted to see whether the relationship between the response (*LnRR*) and the degree of warming (*LnSR*) was linear (see Appendix 6 for methods and results of the sensitivity, publication bias and linearity tests).

#### 2.4.1 End‐of‐century scenario

All the predictor models above were re‐run using a subset of the data where responses were only included if the experimental temperatures were above the MAT predicted for the end of the century (+2.58°C, RCP8.5 global mean sea surface temperature projection 2081–2100; IPCC, 2019) at the collection sites. This allowed the assessment of whether there is evidence of an increased negative impact on echinoderms when experiments use magnitudes of warming tantamount to end‐of‐century predictions, and whether testing temperatures that are already frequently experienced by the organism may underestimate the potential effects of future warming (see File S2 for code for all models, including sensitivity analyses and publication bias detection).

## 3 RESULTS

**FIGURE 1:**
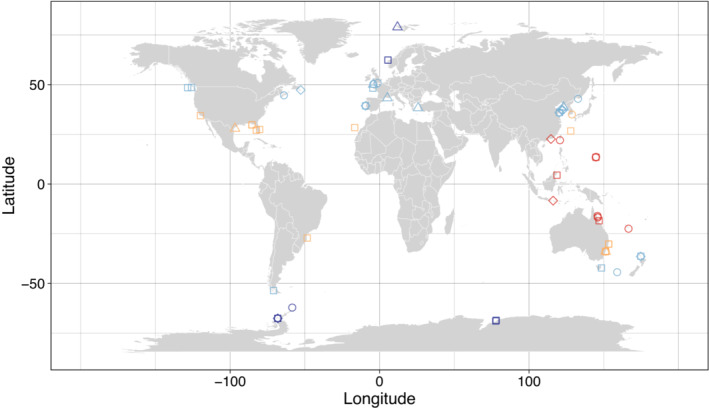
World map illustrating the collection locations of the echinoderms included in this meta‐analysis. The points are colour coded by whether the organisms were found in tropical (red), subtropical (yellow), temperate (light blue) or polar (dark blue) waters. The shapes represent the four classes of echinoderm included in the meta‐analysis: Asteroidea (circles), Echinoidea (squares), Holothuroidea (diamonds) and Ophiuroidea (triangles).

A total of 85 studies met the selection criteria for the ‘biological response’ model. The dataset contained 710 individual responses from 47 species of echinoderm from around the globe (Asteroidea, *n* = 13; Echinoidea, *n* = 21; Holothuroidea, *n* = 6; Ophiuroidea, *n* = 7; Figure 1; see Appendix 5). The models containing all other predictors excluded five of the 85 studies, which included 52 individual responses measuring the effect of temperature on metabolic rate. The effect sizes (converted to a percentage change) are based on the average magnitude of warming across the dataset (~5°C).

**FIGURE 2:**
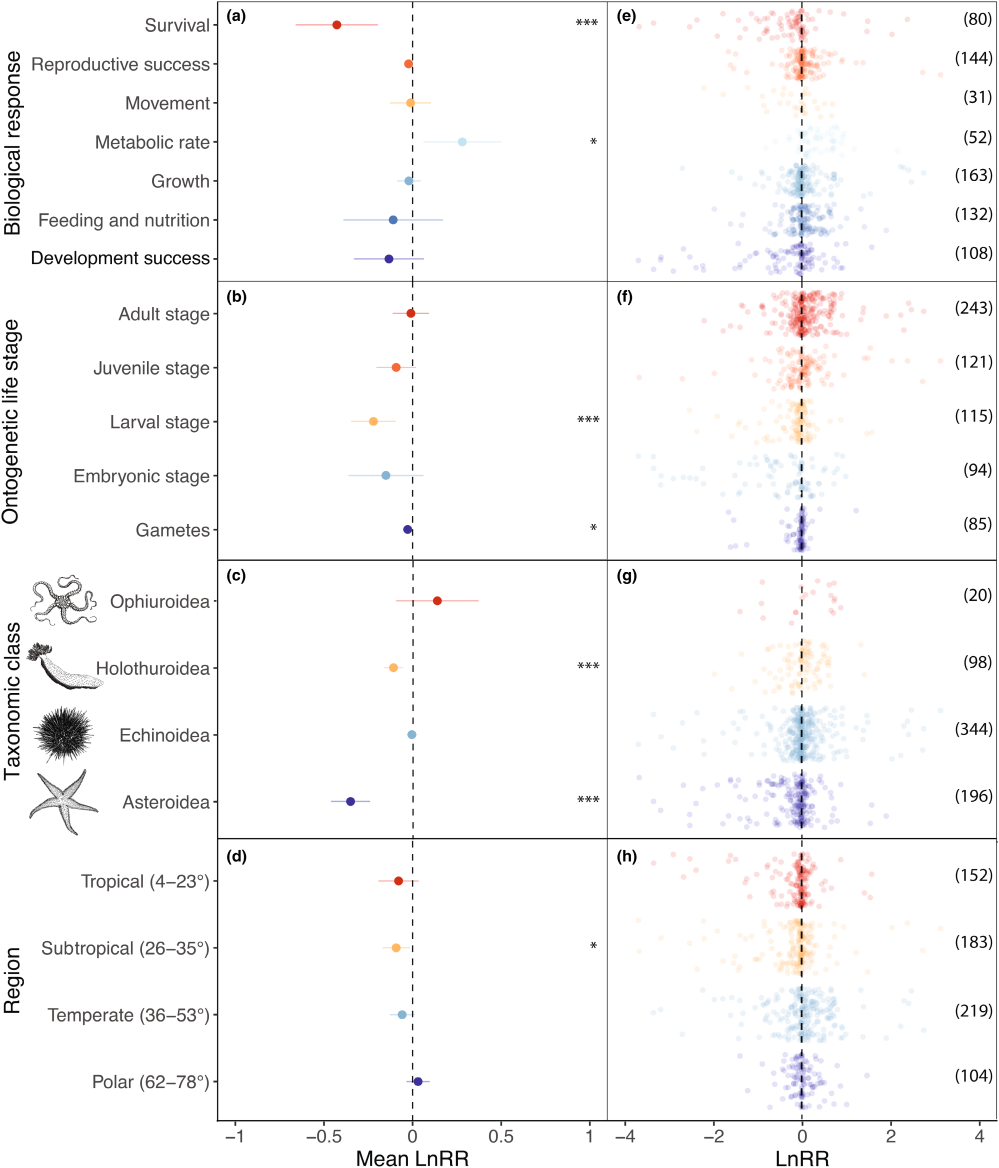
Effect of warming on echinoderms from around the globe, a comparison between biological responses (a, e), ontogenetic life stages (b, f), taxonomic classes (c, g) and regions (d, h). The mean effect sizes and the robust 95% confidence intervals are provided (left panels, a–d), as well as the raw data points (right panels, e–h). The number of responses in each group are provided in parentheses in the right panels. Significant mean effect sizes are indicated by an asterisk (*p < .05; **p < .01; ***p < .001) in the left panels. Mean effect sizes and 95% confidence intervals have been corrected for differences between the control and experimental temperatures (*LnSR*). Effect sizes above and below zero indicate a positive and negative response to warming, respectively.

The response to warming was found to vary in magnitude and direction among the different biological responses considered (Robust *Q*_M_; *F* = 5.00, df = 7,78, *p* < .001; Figure 2a,e; see File S3 for robust model outputs for all predictors). Survival was reduced by 35% on average (*p* < .001), while metabolic rate was the only biological response that increased with warming, and was on average 32% higher (*p* = .013). There was a negative effect of warming on both development success as well as feeding and nutrition (12% and 10% decline, respectively); however, these effects were not significant. In the case of development success, there was evidence of non‐linearity in the response, which could have resulted in the lack of significance (see Appendix 6.3). The effects of warming on growth, movement and reproductive success were not distinguishable from zero (*p* > .050).

Warming affected echinoderm life stages to differing extents (Robust *Q*_M_; *F* = 4.65, df = 5,75, *p* = .001; Figure 2b,f). The larval stage was the most vulnerable to warming, with elevated temperature leading to a 20% decline in performance (*p* < .001). To a lesser extent, gametes (including fertilisation) were also significantly negatively affected (*p* = .039); however, their performance only declined by 3% on average. This contradictory finding may be due to negative outliers and a relatively small sample size (*n* = 85 responses), that biased results towards significance. Despite the effect sizes not being significant, embryos and juveniles were the second and third most negatively affected life stages (based on percentage declines in performance), with a 14% (*p* = .162) and 9% (*p* = .103) decline in performance, respectively. The average *LnRR* was close to zero for adults, suggesting no overall effects of warming on this life stage (*p* = .855).

Vulnerability to warming was significantly different between taxonomic classes of echinoderms (Robust *Q*_M_; *F* = 14.74, df = 4,76, *p* < .001; Figure 2c,g). Asteroidea were the most negatively affected, with an average 30% decline in performance (*p* < .001). Holothuroidea exhibited a 10% decline in performance (*p* < .001). Ophiuroidea appeared to respond positively to thermal challenge, with a 15% increase in performance, but this was not significant (*p* = .244). Considering the relatively even spread of data points around zero for this class, the high estimate may be a consequence of low resolution due to the small sample size (*n* = 20 responses). For Echinoidea, the mean *LnRR* was close to zero (*p* = .638). Removal of the three species that contributed the most data points changed the model output very little (see Appendix 6.1).

The response to warming varied based on the region (Robust *Q*_M_; *F* = 2.84, df = 4,76, *p* = .030; Figure 2d,h); however, only echinoderms from subtropical (26–35°) latitudes were significantly affected by warming (*p* = .020), with an average 9% reduction in performance. Tropical (4–23°) and temperate (36–53°) echinoderms exhibited 8% and 6% declines in performance, respectively, while polar (62–78°) echinoderms experienced slight (3%) increases in performance (all *p* > .050). Tropical echinoderms exhibited non‐linearity in their response, which may explain the non‐significant mean effect (see Appendix 6.3). When the tropical *Acanthaster* sp. were removed from the analysis, the vulnerability tropical echinoderms became significantly negatively affect by warming (*p* = .040), although the percentage change was 5% lower (see Appendix 6.1). This discrepancy may be a consequence of the much smaller sample size for tropical echinoderms (*n* = 42 data points) after the removal of this species (which contributed *n* = 110 data points). The removal of *Apostichopus japonicus* (*n* = 78 data points) and *Strongylocentrotus droebachiensis* (*n* = 53 data points) individually from the analysis did not significantly alter the response in the different region groups. However, the removal of *A. japonicus* resulted in the overall effect of the model becoming nonsignificant (*p* = .086; see Appendix 6.1).

Differences in the experimental design (i.e. habituation time at the control temperature, warming rate, and exposure time at the experimental temperature) had no significant effect on echinoderm responses (*p* > .050). For all predictor models, there was significant residual heterogeneity (*p* < .001).

### 3.1 End‐of‐century scenario

When only the responses that applied experimental temperatures relevant to end‐of‐century projections were tested, it was found that for some of the groups within predictors, the effect size and/or significance had changed (see Appendix 7 for a figure illustrating these results). For instance, metabolic rate was no longer significantly positively impacted by warming, and gametes were no longer significantly negatively impacted by warming (*p* > .050), yet the negative impact of warming on juveniles increased by 31% (*p* < .001). Under this end‐of‐century scenario, Asteroidea and Holothuroidea were also more negatively affected, with performance declining by a further 21% (*p* < .001) and 18% (*p* = .049), respectively. There was also a further 26% decline in performance of echinoderms from tropical (4–23°) latitudes, although this group still failed to exceed the threshold for significance (*p* > .050). Subtropical (26–35°) echinoderms were no longer significantly impacted by warming under the end‐of‐century scenario (*p* = .384); however, the percentage change in performance remained the same.

## 4 DISCUSSION

For echinoderms, finding patterns in vulnerability to warming between biological responses, life stages, taxonomic classes or regions will aid the ability to predict how species and populations will respond to future climate change. The present meta‐analysis found a number of interesting patterns. While warming accelerated metabolic rates and reduced survival, several biological responses were robust to thermal challenge, including growth, feeding and nutrition, movement and reproduction. This may suggest that these biological responses are not good predictors of the vulnerability of echinoderms to ocean warming, as their performance can be maintained up until thermal limits are reached. The findings presented herein also suggest that under greater ocean warming, the larval stage may act as a developmental bottleneck in the life cycle of echinoderms, asteroids may be the most vulnerable group of echinoderms, and that populations from warmer latitudes may be living particularly close to their thermal limit. This information may facilitate predictions of how various ecologically and economically important echinoderms may fare in a warmer ocean, and will aid decision making for marine natural resources managers, and policy makers (Kroeker et al., 2010).

It is widely accepted that for ectotherms, metabolic rate increases up until a critical temperature, after which energy production can no longer keep pace with the energetic demands for the maintenance of homeostasis and normal behaviours (Pörtner et al., 2017; Schulte, 2015; Schulte et al., 2011). As expected, there was a significant (32%) increase in metabolism in echinoderms with warming, testament to the acceleratory effects of warming on cell processes (Schulte, 2015). Under the end‐of‐century experimental conditions the effect size for metabolic rate was less positive, indicating that echinoderms may live closer to their physiological thermal limits by 2100. Interestingly, while similar positive metabolic responses to warming were found in other meta‐analyses on marine invertebrates and fishes, they have generally found far lower metabolic rate increases for similar magnitudes of warming (~7%–17% increase with 3–4°C of warming on average; Hu et al., 2022; Pereira Santos et al., 2021; Sampaio et al., 2021). A comparable response could have been anticipated for development success, as this would also be expected to shift due to an increase in the rate of cell processes (Munday et al., 2008; Pörtner et al., 2017; Pörtner & Peck, 2010; Schulte, 2015). However, there was no evidence that warming increased the speed at which early life stages reached developmental milestones, and more than ~3°C of warming was particularly detrimental to development success. Consequently, when considering warming consistent with end‐of‐century predictions, development success declined by 35%, indicating a possible negative effect of temperature on the cell cycle (Schulte, 2015), which may be further exacerbated by other anthropogenic stressors, such as ocean acidification (Byrne, 2011; Kroeker et al., 2010; Przeslawski et al., 2015). Developmental success may be more closely linked with survival, considering that slower development may indicate incompetency, and is associated with a higher risk of predation in the field (Munday et al., 2008; Ross et al., 2011).

Mortality due to supra‐optimal thermal exposure will ultimately lead to declines in abundance, population viability, and even extinctions (Byrne, 2011; Eisenlord et al., 2016). A 35% decline in survival was observed across all echinoderms. Consistent with the findings in the present study, Sampaio et al. (2021) observed a ~32% reduction in survival for a variety of marine taxa (including corals, crustaceans, echinoderms, molluscs and fishes), but for an average 3°C of warming. In contrast, elasmobranch survival was robust, with a mere ~3% reduction in survival with ~4°C of warming (Pereira Santos et al., 2021). Surprisingly, although echinoderm survival declined with thermal challenge in the current analysis, limited impacts on other biological responses such as growth, feeding or movement were found. This is concerning, as previous studies have considered these metrics as indicators of sensitivity to climate change (Pereira Santos et al., 2021; Sampaio et al., 2021). Since these biological responses were not affected by warming in the models presented herein, echinoderms may maintain these behaviours and processes in a warmer ocean, leading to an energy deficit, and subsequent mortality. Behaviour is often expected to be the first response to environmental change as a means for individuals to buffer the negative effects (Wong & Candolin, 2015). However, since echinoderms are relatively limited in their mobility, they may have reduced capacity to alter their behaviour in response to warming. Given the relatively short experimental durations in most ex situ studies, it must be considered that behavioural responses to temperature may occur after lengthier exposure durations. Nevertheless, if echinoderm metabolic rate and hence energetic demands increase greatly with warming, while feeding and nutrition and therefore energy intake do not increase proportionally, an overall energy shortfall is expected during projected warming periods (Lawrence, 1984; Munday et al., 2008; Pörtner & Peck, 2010; Schulte, 2015). This may result in widespread mortality and population contractions (Eisenlord et al., 2016; Munday et al., 2008; Poloczanska et al., 2016).

In the life cycle of marine ectotherms, highly fecund adults immediately prior to and after spawning, as well as the planktonic early life stages are often considered the most vulnerable to ocean warming, and these stages will likely determine population persistence into the future (Dahlke et al., 2020). The present meta‐analysis found only partial support for this expectation, with reproduction and gametes being relatively robust to warming (3% decline in performance with warming), even when exploring only end‐of‐century relevant responses. It appears the negative impact of warming may increase as development progresses from gametes (including the process of fertilisation) to embryos and finally to larvae, respectively. A 20% decline in performance was observed at this latter planktonic life stage, and the greatest declines in larval performance were observed when warming exceeded ~3°C. These observations may be explained by protective maternal factors (e.g. heat shock proteins), which shield offspring from extreme environmental conditions, but have more of an effect in gametes and embryos than larvae (Byrne, 2011; Foo et al., 2012; Hamdoun & Epel, 2007; Lockwood et al., 2017; Sconzo et al., 1986). The findings presented herein are corroborated by those from previous studies, including meta‐analyses, which indicate a greater relative vulnerability of the larval stage to extreme environmental conditions, including ocean warming, ocean acidification, reduced salinity, and hypoxia (Byrne, 2011; Dahlke et al., 2020; Harvey et al., 2013; Kroeker et al., 2010; Pandori & Sorte, 2018; Przeslawski et al., 2015; Sampaio et al., 2021). Notably, ocean acidification appears to significantly reduce the calcification rate of marine larvae (Byrne, 2011; Harvey et al., 2013; Kroeker et al., 2010). Reduced larval performance under further climate change is concerning, as this will likely interfere with settlement success and recruitment into the population (Pörtner et al., 2017; Ross et al., 2011). Recruitment success may be further hindered by declines in performance of juvenile echinoderms, when exposed to magnitudes of warming consistent with future predicted temperatures. Juveniles may have a reduced physiological capacity and ability to exhibit adaptive strategies (e.g. aestivation and burrowing) to buffer the effects of supra‐optimal temperatures, compared to adults, which were found to be thermally robust even under end‐of‐century conditions (Christensen et al., 2017; Dahlke et al., 2020; Marshall et al., 2013; Nguyen et al., 2011; Pandori & Sorte, 2018; Sampaio et al., 2021; Zamora & Jeffs, 2012). Contrary to expectations, adult reproduction showed no measurable decline in response to thermal challenge. However, the thermosensitivity of reproduction likely requires further investigation into echinoderms with different modes of reproduction (e.g. brooding versus broadcasting), which could not be explored here due to the lack of studies on brooding species (Menge, 1975; Pechenik, 1999). Despite the relative consistency of patterns of vulnerability to warming between life stages in prior research (Byrne, 2011; Dahlke et al., 2020; Pandori & Sorte, 2018; Przeslawski et al., 2015; Sampaio et al., 2021), comparisons warrant some caution, considering that the measures used to assess responses to warming often differ between ontogenetic life stages, particularly between the planktonic and benthic stages (Pottier et al., 2022). Furthermore, some life stages have significantly fewer applied measures of performance than others. For instance, to measure the vulnerability of gametes to warming, most studies investigate fertilisation success (see File S1).

Variation in the response to warming in marine ectotherms may be attributable to phylogenetic or evolutionary differences, leading to variabilities in thermal tolerance windows and adaptive strategies (Bennett et al., 2021; Christensen et al., 2017; Marshall et al., 2013; Pereira Santos et al., 2021; Sampaio et al., 2021; Zamora & Jeffs, 2012). It was determined that both the classes Asteroidea and Holothuroidea were negatively impacted by warming, while Ophiuroidea and Echinoidea were thermally robust. However, this pattern was not clearly related to phylogeny as Asteroidea and Ophiuroidea cluster in the subphylum Asterozoa while Holothuroidea and Echinoidea are in the subphylum Echinozoa (Reich et al., 2015). The pattern may, however, be linked to variations in metabolic processes and the relative allocation of resulting energy to various body components, behaviours (e.g. movement) and processes (e.g. reproduction) within the body, which differs between taxonomic classes (Lawrence, 1984; Whitehill & Moran, 2012). A comparative study on echinoid (*Arbacia punctulata*) and ophiuroid (*Ophiocoma alexandri*) larvae, found that metabolic rates were lower in ophiuroids, suggested to be a consequence of their lower feeding rates which cannot sustain greater energy requirements (Whitehill & Moran, 2012). A lower metabolic rate in the ophiuroid larvae compared to the Echinoid larvae may be advantageous when food is limited, but a concomitant slower development rate may offset this benefit (Whitehill & Moran, 2012). In the present analysis, asteroids were deemed the most negatively affected by warming (30% decline in performance) and their performance declined by an even greater extent when only considering responses where warming magnitudes were in line with end‐of‐century predictions. In contrast to asteroid performance, ophiuroids showed trends of enhanced performance with warming. However, due to the paucity of studies on this class, this analysis may not have had the resolution to detect any negative effects. An ability to make generalisations of thermal performance in different taxonomic groups may be helpful when estimating the responses of species, particularly those that have the ability to alter the structure and function of an ecosystem, and further research on under‐represented taxa are warranted (Ling, 2013; Menge et al., 2016).

The findings in this meta‐analysis, to some extent, supports the general trend that the vulnerability of marine ectotherms to elevated temperatures increases with latitude from the poles to the equator, likely due to the increase in thermal stability (Hughes et al., 2018; Pinsky et al., 2019; Poloczanska et al., 2016; Sunday et al., 2011; Woolsey et al., 2015). When all data were included, echinoderms from subtropical latitudes, were the most negatively impacted by warming (9% decline in performance), and tropical echinoderms only exhibited declines in performance with ~4°C of warming. Although, removal of *Acanthaster* spp. led to tropical echinoderms becoming significantly negatively affected by warming. Furthermore, the negative impact was 25% greater for tropical compared to subtropical echinoderms in the end‐of‐century scenario, due to non‐linearity in the thermal response. While temperate echinoderms were more tolerant to warming, there was still an overall negative effect on performance, which more than doubled in the end‐of‐century scenario. In contrast, there was an overall positive impact of warming on polar echinoderms, which became three times more positive in the aforementioned scenario. It is necessary to consider, that since environmental conditions not only vary on a global scale, but also on a local scale, this may have had some influence on the patterns observed across regions. For instance, shallow water species in tidal environments experience greater diurnal temperature variation in comparison to deeper water species and may also be living closer to their thermal limits (Drake et al., 2017; Pey et al., 2011; Sunday et al., 2011).

### 4.1 Data gaps, limitations and future directions

The present meta‐analysis identified a number of data gaps and limitations. First, the authors were unable to gather sufficient information from studies in order to establish the effect of local scale thermal variability on the vulnerability to warming. Additionally, sufficient data on the spatial distribution of echinoderm species is lacking, and it is possible that responses to ocean warming may differ depending on the breadth of the species thermal range, and whether the individuals were collected from the leading or trailing edges, or the central part of the range (Collin et al., 2018; O'Connor et al., 2012; Poloczanska et al., 2016; Sasaki et al., 2022; Sunday et al., 2011; Zettlemoyer & Peterson, 2021). Some classes of echinoderms were underrepresented in the dataset, specifically there were only 20 data points for ophiuroids, and astonishingly, the authors did not find relevant studies on crinoids during the literature search. There were also relatively few studies that covered multiple life stages or generations, which are important to understand the plastic and adaptive capacity of echinoderms (Byrne et al., 2020; Pandori & Sorte, 2018; Przeslawski et al., 2015; Suckling et al., 2015; Uthicke et al., 2021; Wernberg et al., 2012). Moreover, many studies were excluded because not all data required for the models were provided in the papers (i.e. means, *n* = 4, error values or sample sizes, *n* = 22, temperatures, *n* = 21; see Appendix 1). A consequence of the fact that many relevant studies could not be included, there was insufficient data available to include multiple predictors in a single model. The detection of publication bias in the dataset suggests that even more studies relevant for this meta‐analysis were not included, because they were simply not published. This bias may have led to the over‐estimation of effects on taxa (Duval & Tweedie, 2000; see Appendix 6.2).

These data gaps and limitations gave rise to the suggestion of several key considerations for future experimental studies on the impacts of ocean warming on echinoderms:

- An increased effort should be made to record, not only co‐ordinates of the collection location, but information regarding the habitat and the geographic range of the species.
- There should be a greater focus on studying groups of echinoderms that are poorly represented in the literature, as well as those that are of high ecological importance.
- There should be an accelerated effort in publishing studies that span multiple life stages and generations.
- All relevant data required for meta‐analyses should be incorporated into the papers, including temperatures, means, standard deviations and sample sizes.
- All results should be published, even if they show non‐significant effects.

A greater number of studies overall would provide the resolution to detect patterns in thermal responses with greater reliability. Moreover, this would allow for the assessment of interactions between multiple predictors in meta‐analytic models, to better forecast the vulnerability of echinoderm species into the future. To provide a more realistic assessment however, it is necessary to consider the myriad of other environmental and ecological factors (e.g. ocean acidification, food availability and predation) that may exacerbate, or even alleviate, echinoderm vulnerability to a warming ocean (Kroeker et al., 2010; Lucey et al., 2020; Melzner et al., 2020; Sampaio et al., 2021; Uthicke et al., 2015). An important next step would be to incorporate multiple stressors associated with climate change, in a meta‐analysis on echinoderm performance, that may have synergistic, additive or antagonistic effects when combined with warming (Harvey et al., 2013; Pandori & Sorte, 2018; Przeslawski et al., 2015; Sampaio et al., 2021).

## 5 CONCLUSIONS

This meta‐analysis provides a critical first step in understanding patterns of thermal responses among echinoderms and adds to the ever‐growing body of literature predicting the negative impacts of climate change on marine species (Byrne, 2011; Dahlke et al., 2020; Pereira Santos et al., 2021; Poloczanska et al., 2016; Sampaio et al., 2021; Sunday et al., 2011). This taxon is clearly vulnerable to ocean warming, and many of the patterns observed in this meta‐analysis fitted with broad expectations for marine ectotherms. For instance, proportionally greater negative responses to warming were observed in subtropical and tropical echinoderms and at larval ontogenetic life stages. However, there were some interesting patterns that did not fit initial expectations, including a lack of thermal vulnerability for many biological responses (i.e. growth, feeding and nutrition, movement, and reproduction).

The impacts of ocean warming and accompanying marine heatwaves are already evident in echinoderm populations, with disease‐induced die‐offs in numerous species being linked to elevated temperatures (Clemente et al., 2014; Dungan et al., 1982; Lester et al., 2007; Menge et al., 2016). Despite this growing threat, echinoderms may be able to persist in warmer oceans through range shifts and/or acclimation and adaptation (Ling et al., 2009; Munday et al., 2008; Poloczanska et al., 2016). However, there is a growing consensus that future ocean warming may be too rapid and marine heatwaves may be too abrupt for these mitigation strategies to keep pace with climate change (Radchuk et al., 2019). Considering the disproportionately large role that echinoderms play in structuring communities and ecosystems, understanding how members of this phylum will fare under further warming is of paramount importance (Byrne & O'Hara, 2017; Ling, 2013; Menge et al., 2016).

## AUTHOR CONTRIBUTIONS

**Bethan J. Lang:** Conceptualization (lead); data curation (lead); formal analysis (lead); funding acquisition (equal); investigation (lead); methodology (lead); project administration (lead); validation (lead); visualization (lead); writing – original draft (lead); writing – review and editing (lead). **Carolyn R. Wheeler:** Formal analysis (supporting); methodology (supporting); writing – original draft (supporting); writing – review and editing (supporting). **Ciemon F. Caballes:** Supervision (equal); writing – original draft (supporting); writing – review and editing (supporting). **Jennifer M. Donelson:** Conceptualization (supporting); formal analysis (supporting); funding acquisition (equal); investigation (supporting); methodology (supporting); supervision (equal); visualization (supporting); writing – original draft (supporting); writing – review and editing (supporting). **Kevin R. Bairos‐Novak:** Formal analysis (supporting); methodology (supporting); writing – original draft (supporting); writing – review and editing (supporting). **Morgan S. Pratchett:** Conceptualization (supporting); funding acquisition (equal); project administration (supporting); supervision (equal); writing – original draft (supporting); writing – review and editing (supporting). **Sven Uthicke:** Supervision (equal); writing – original draft (supporting); writing – review and editing (supporting).

## CONFLICT OF INTEREST STATEMENT

The authors declare no competing interests.

## Supporting information


File S1

File S2

File S3

## Data Availability

Data and code used for this study are available at Research Data JCU https://doi.org/10.25903/2k76‐ym17.

## APPENDIX 1

### 1.1

Flow diagram illustrating how the literature was searched and screened for eligibility for inclusion in the meta‐analysis. The numbers of studies included and excluded (for various reasons) are detailed.

## APPENDIX 2

### 2.1

Data sources included in the meta‐analysis. * indicates papers measuring metabolic rate that were removed from the main analyses for all moderators except ‘biological response’. ** indicates papers where the experimental temperature was below the near‐future mean annual temperature projection, and were therefore removed from the associated subset of data.

## APPENDIX 3

### 3.1

Predictors of variation in echinoderm vulnerability to warming, tested using separate multivariate linear mixed effects models. The predictor name and groups within them are provided.

**Table:**

Model predictor	Groups
Biological response	Development success, feeding and nutrition, growth, metabolic rate, movement, reproductive success, survival
Life stage	Gametes (including fertilisation), embryonic stage, larval stage, juvenile stage, adult stage
Class	Asteroidea, Echinoidea, Holothuroidea, Ophiuroidea
Region	Tropical (4–23°), sub‐tropical (26–35°), temperate (36–53°), polar (62–78°)
Habituation time at the control temperature (days)	Continuous
Warming rate (°C h^−1^)	Continuous
Exposure time at experimental temperature (days)	Continuous

## APPENDIX 4

### 4.1

List of measures tested within each ‘biological response’ group.

**Table:**

Biological response group	Measures
Development success	Percentage undergoing cleavage/gastrulation/hatching; percentage reaching various larval stages; development rate/time; larval arm asymmetry; percentage settled; percentage metamorphosing into juveniles
Feeding and nutrition	Feeding rate/total food consumed; proportion of individuals feeding; absorption efficiency; feed conversion efficiency; energy consumed; food encounter rate/capture probability; protein/lipid/carbohydrate content; protein/lipid/carbohydrate absorption efficiency
Growth	Body length/width/height; body area/radius/diameter/volume; growth rate; wet weight/ash free dry weight; number of arms/spines; ciliated band length Mg/Ca ratio; scope for growth; net growth efficiency; percentage intact individuals/regeneration rate/functional recovery/percentage regained arms; muscle density
Metabolic rate	Larval oxygen consumption rate; standard/resting/routine oxygen consumption rate
Movement	Righting time/number of successful righting attempts; locomotion speed; percentage individuals moving; foraging time; percentage exhibiting covering behaviour; tentacle locomotion time
Reproductive success	Sperm speed/motility; percentage of successful fertilisations; sperm mitochondrial activity; egg diameter/volume/sphericity; gonado‐somatic index/gonad growth/gonad maturity index
Survival	Percentage mortality/survival of embryos/larvae/juveniles; percentage abnormal embryos/larvae/juveniles; percentage of blastulae released from egg membrane; density of larvae; percentage settlement and post‐settlement survival

## APPENDIX 5

### 5.1

Table of species of echinoderm included in the analysis, their taxonomic class and region groupings and the number of data points for each species.

**Table:**

Species	Class	Region	Number of points
*Acanthaster* sp.	Asteroidea	Tropical	110
*Apostichopus japonicus*	Holothuroidea	Temperate	78
*Arbacia lixula*	Echinoidea	Sub‐tropical	3
*Arbacia punctulata*	Echinoidea	Sub‐tropical	22
*Asterias amurensis*	Asteroidea	Sub‐tropical	6
*Asterias rubens*	Asteroidea	Temperate	5
*Asterias vulgaris*	Asteroidea	Temperate	9
*Asterina pectinifera*	Asteroidea	Temperate	3
*Australostichopus mollis*	Holothuroidea	Temperate	12
*Centrostephanus rodgersii*	Echinoidea	Temperate	22
*Cucumaria frondosa*	Holothuroidea	Temperate	10
*Culcita novaeguineae*	Asteroidea	Tropical	3
*Dendraster excentricus*	Echinoidea	Temperate	8
*Diadema africanum*	Echinoidea	Sub‐tropical	3
*Diadema savignyi*	Echinoidea	Tropical	3
*Echinometra lucunter*	Echinoidea	Sub‐tropical	10
*Echinometra mathaei*	Echinoidea	Tropical	12
*Echinometra* sp. *A*	Echinoidea	Tropical	1
*Evechinus chloroticus*	Echinoidea	Temperate	5
*Heliocidaris erythrogramma*	Echinoidea	Sub‐tropical	39
*Heliocidaris tuberculata*	Echinoidea	Sub‐tropical	6
*Holothuria forskali*	Holothuroidea	Temperate	8
*Holothuria moebii*	Holothuroidea	Tropical	2
*Holothuria scabra*	Holothuroidea	Tropical	8
*Linckia laevigata*	Asteroidea	Tropical	5
*Loxechinus albus*	Echinoidea	Temperate	4
*Lytechinus variegatus*	Echinoidea	Sub‐tropical	49
*Marthasterias glacialis*	Asteroidea	Temperate	3
*Meridiastra calcar*	Asteroidea	Sub‐tropical	9
*Microphiopholis gracillima*	Ophiuroidea	Sub‐tropical	1
*Odontaster validus*	Asteroidea	Polar	21
*Ophiocten sericeum*	Ophiuroidea	Polar	5
*Ophioderma longicauda*	Ophiuroidea	Temperate	10
*Ophionereis schayeri*	Ophiuroidea	Sub‐tropical	1
*Ophiopholis mirabilis*	Ophiuroidea	Temperate	2
*Ophiopholis sarsii*	Ophiuroidea	Temperate	2
*Ophiura ophiura*	Ophiuroidea	Temperate	6
*Paracentrotus lividus*	Echinoidea	Sub‐tropical/Temperate	45
*Parvulastra exigua*	Asteroidea	Sub‐tropical	12
*Patiriella pseudoexigua*	Asteroidea	Tropical	4
*Patiriella regularis*	Asteroidea	Temperate	8
*Pseudechinus magellanicus*	Echinoidea	Temperate	4
*Sphaerechinus granularis*	Echinoidea	Sub‐tropical	6
*Sterechinus neumayeri*	Echinoidea	Polar	37
*Strongylocentrotus droebachiensis*	Echinoidea	Temperate/Polar	53
*Strongylocentrotus purpuratus*	Echinoidea	Sub‐tropical	2
*Tripneustes gratilla*	Echinoidea	Tropical/Sub‐tropical	33

## APPENDIX 6

### 6.1

Sensitivity, publication bias and non‐linearity tests methods and results.

#### 6.1 Sensitivity analyses

##### Methods

The presence of influential observations was tested by ranking the data by the effect size and removing the observations with the largest 10 effect sizes (in either direction) in a stepwise fashion and re‐running all categorical models (i.e., biological response, ontogenetic life stage, taxonomic class, and region), to see if the overall significance of *Q*_M_, and the significance of individual groups of a predictor were affected. The effect of pseudoreplication was then explored by removing publications one at a time that contributed two or more ‘studies’ due to measuring responses of multiple species and observing any changes in significance. Again, this was conducted for each of the categorical predictors. Lastly, the ontogenetic life stage and taxonomic class models were re‐run, removing the three species that contributed the most data points: *Acanthaster* spp. (tropical asteroid; *n* = 110 data points), *Apostichopus japonicus* (temperate holothuroid; *n* = 78 data points) and *Strongylocentrotus droebachiensis* (temperate and polar echinoid; *n* = 53 data points).

##### Results

These models were robust to the removal of the 10 responses with the highest effect sizes (in either direction). Removal of papers that contribute two or more studies, in most cases did not change the model outputs to a significant extent. However, the responses reported in Rupp (1973) which contributed six studies to the analysis appears to be particularly influential in two models. In the ontogenetic life stage model, gametes (and fertilisation) were no longer being significantly impacted by warming when responses from this paper were removed (*p* = .661). Furthermore, in the region model, echinoderms from tropical (4–22°) latitudes became significantly negatively affected by warming (*p* = .025), when Rupp (1973) was removed. Moreover, removing Garcia et al. (2018) that contributed four studies to the meta‐analysis, resulted in sub‐tropical (26–35°) echinoderms being no longer significantly impacted by warming (*p* = 0.079), along with the overall Robust *Q*_M_ (*p* = .077). Removal of *Acanthaster* spp. resulted in tropical echinoderms becoming significantly affected by warming (*p* = .040), and removal of *Apostichopus japonicus* resulted in the overall effect of the model becoming non‐significant (*p* = .086). In all other instances the significances and direction of the response remained the same for the overall predictor models, and the individual groups.

In the tables, the groups in which the significance changed from the original models are in bold.

Life stage model output after removal of Rupp (1973).

**Table:**

Life stage	Estimate	SE	*t* Value	df	*p* Value	ci.lb	ci.ub
**Gametes**	**−0.0127**	**0.0288**	**−0.4407**	**69**	**.6608**	**−0.07**	**0.0447**
Embryonic stage	−0.1981	0.1499	−1.3218	69	.1906	−0.497	0.1009
Larval stage	−0.222	0.0633	−3.5059	69	.0008	−0.3483	−0.0957
Juvenile stage	−0.0929	0.0564	−1.648	69	.1039	−0.2054	0.0196
Adult stage	−0.0096	0.0516	−0.1857	69	.8532	−0.1126	0.0934

*Q*_M_; *F* = 3.21, df = 5,69, *p* = .012, *n* = 630.

Region model output after removal of Rupp (1973).

**Table:**

Region	Estimate	SE	*t* Value	df	*p* Value	ci.lb	ci.ub
**Tropical (4–23°)**	**−0.2496**	**0.1087**	**−2.2972**	**70**	**.0246**	**−0.4664**	**−0.2496**
Sub–tropical (26–35°)	−0.0928	0.0391	−2.3701	70	.0205	−0.1709	−0.0928
Temperate (36–53°)	−0.059	0.0348	−1.6961	70	.0943	−0.1284	−0.059
Polar (62–78°)	0.0308	0.033	0.9312	70	.3549	−0.0351	0.0308

*Q*_M_; *F* = 3.66, df = 4,70, *p* = .009, *n* = 630.

Region model output after removal of Garcia et al. (2018).

**Table:**

Region	Estimate	SE	*t* Value	df	*p* Value	ci.lb	ci.ub
Tropical (4–23°)	−0.079	0.0572	−1.3807	72	.1716	−0.193	−0.079
**Sub–tropical (26–35°)**	**−0.1209**	**0.0679**	**−1.7808**	**72**	**.0792**	**−0.2562**	**−0.1209**
Temperate (36–53°)	−0.0589	0.0348	−1.6946	72	.0945	−0.1282	−0.0589
Polar (62–78°)	0.0308	0.033	0.9337	72	.3536	−0.035	0.0308

***Q***_**M**_ **; *F* = 2.21, df = 4,72, *p* = .077, *n* = 637.**

Class model output after removal of *Acanthaster* spp.

**Table:**

Class	Estimate	SE	*t* Value	df	*p* Value	ci.lb	ci.ub
Asteroidea	−0.3668	0.1609	−2.2794	68	.0258	−0.6880	−0.0457
Echinoidea	−0.0066	0.0141	−0.4705	68	.6395	−0.0348	0.0215
Holothuroidea	−0.1101	0.0269	−4.0968	68	.0001	−0.1637	−0.0565
Ophiuroidea	0.1360	0.1179	1.1537	68	.2527	−0.0993	0.3713

*Q*_M_; *F* = 5.88, df = 4,68, *p* < .001, *n* = 548.

Region model output after removal of *Acanthaster* spp.

**Table:**

Region	Estimate	SE	*t* Value	df	*p* Value	ci.lb	ci.ub
**Tropical (4–23°)**	**−0.0280**	**0.0133**	**−2.0974**	**68**	**.0397**	**−0.0546**	**−0.0014**
Sub–tropical (26–35°)	−0.0927	0.0391	−2.3725	68	.0205	−0.1707	−0.0147
Temperate (36–53°)	−0.0589	0.0348	−1.6926	68	.0951	−0.1284	0.0105
Polar (62–78°)	0.0308	0.0331	0.9312	68	.3550	−0.0352	0.0967

*Q*_M_; *F* = 3.44, df = 4,68, *p* = .013, *n* = 548.

Class model output after removal of *Apostichopus japonicus*.

**Table:**

Class	Estimate	SE	*t* Value	df	*p* Value	ci.lb	ci.ub
Asteroidea	−0.3549	0.0557	−6.3776	71	<.0001	−0.4659	−0.2440
Echinoidea	−0.0067	0.0141	−0.4758	71	.6357	−0.0348	0.0214
Holothuroidea	−1.0628	0.5004	−2.1241	71	.0371	−2.0605	−0.0651
Ophiuroidea	0.1331	0.1156	1.1512	71	.2535	−0.0974	0.3636

*Q*_M_; *F* = 11.68, df = 4,71, *p* < .001, *n* = 592.

Region model output after removal of *Apostichopus japonicus*.

**Table:**

Region	Estimate	SE	*t* Value	df	*p* Value	ci.lb	ci.ub
Tropical (4–23°)	−0.0790	0.0572	−1.3803	71	.1718	−0.1930	0.0351
Sub–tropical (26–35°)	−0.0926	0.0389	−2.3798	71	.0200	−0.1703	−0.0150
Temperate (36–53°)	−0.0012	0.0442	−0.0267	71	.9788	−0.0893	0.0869
Polar (62–78°)	0.0308	0.0330	0.9334	71	.3538	−0.0350	0.0966

***Q***_**M**_ **; *F* = 2.11, df = 4,71, *p* = .086, *n* = 592.**

Class model output after removal of *Strongylocentrotus droebachiensis*.

**Table:**

Class	Estimate	SE	*t* Value	df	*p* Value	ci.lb	ci.ub
Asteroidea	−0.3522	0.0554	−6.3601	73	<.0001	−0.4625	−0.2418
Echinoidea	−0.0169	0.0105	−1.6190	73	.1098	−0.0378	0.0039
Holothuroidea	−0.1101	0.0268	−4.1072	73	.0001	−0.1636	−0.0567
Ophiuroidea	0.1361	0.1170	1.1630	73	.2486	−0.0971	0.3693

*Q*_M_; *F* = 15.32, df = 4,73, *p* < 0.001, *n* = 605.

Region model output after removal of *Strongylocentrotus droebachiensis*.

**Table:**

Region	Estimate	SE	*t* Value	df	*p* Value	ci.lb	ci.ub
Tropical (4–23°)	−0.0790	0.0572	−1.3812	73	.1714	−0.1929	−0.0790
Sub–tropical (26–35°)	−0.0927	0.0389	−2.3800	73	.0199	−0.1703	−0.0927
Temperate (36–53°)	−0.0588	0.0349	−1.6857	73	.0961	−0.1283	−0.0588
Polar (62–78°)	−0.0310	0.0520	−0.5964	73	.5528	−0.1346	−0.0310

*Q*_M_; *F* = 2.69, df = 4,73, *p* = .038, *n* = 605.

#### 6.2 Publication bias

##### Methods

Publication bias commonly occurs in meta‐analyses because studies are more likely to be published if results are significant (Duval & Tweedie, 2000; Rosenberg, 2005; Shi & Lin, 2019). To test for publication bias, Duval and Tweedie's trim‐and‐fill method (Duval & Tweedie, 2000) was used, which supresses the most extreme data points on either side of a funnel plot, and by doing so, estimates the number of missing responses due to publication bias in the dataset (Duval & Tweedie, 2000; Sampaio et al., 2021; Shi & Lin, 2019). This method provides conservative estimates, as it does not take into account the random effects (Duval & Tweedie, 2000).

##### Results

Evidence of publication bias was detected, with an estimated 168 responses missing on the left (i.e., negative *LnRR*'s) side. An estimated 150 responses were missing on the left side, when metabolic rate responses were removed. It was estimated that there were no responses missing on the right (i.e., positive *LnRR*'s) side.

#### 6.3 Linearity

##### Methods

It is a possibility that the response (*LnRR*) may be dependent on the degree of warming (*LnSR*), leading to non‐linearity (i.e., thermal performance curves). Any non‐linearity in thermal effects on performance was tested for each group of a predictor using polynomial linear models. For those groups where a second or third order polynomial was detected, where relevant, the degree of warming in which *LnRR* became non‐linear in a negative direction was recorded.

##### Results

Non‐linearity in the correlation between *LnSR* and *LnRR* was observed for some groups for the biological responses, life stages and regions. Most notably, the relationship between *LnSR* and *LnRR* for both development success and larvae is a second order polynomial, with deviation from linearity in the negative direction observed at a *LnSR* of 1.2 (equivalent to 3.3°C of warming) in both instances. Second order polynomials are also detected for embryos and the tropical (4–23°) latitude, with negative deviations from linearity detected at *LnSR*'s of 1.1 (equivalent to 3°C of warming) and 1.4 (equivalent to 3.9°C of warming), respectively.

In the tables, *LnSR* (the natural logarithm of the degree of warming) is the *LnSR* at which a clear negative deviation from linearity is observed for the relationship between this variable and the effect size (*LnRR*). If the relationship is linear ‘NA’ is given, and if a second or third order polynomial is detected, but there is no clear point at which there is a deviation from linearity ‘NA*’ is given. Exp‐Ctl is the degree of warming at which the non‐linearity is observed, that is, the back transformation of *LnSR*.

Detection of non‐linearity in the biological response groups.

**Table:**

Biological response	Relationship	LnSR	Exp‐Ctl (°C)
**Development**	**Second order polynomial**	**1.17**	**3.25**
Feeding	Linear	NA	NA
Growth	Linear	NA	NA
Metabolism	Linear	NA	NA
Movement	Linear	NA	NA
Reproduction	Third order polynomial	NA*	NA
Survival	Linear	NA	NA

Detection of non‐linearity in the life stage groups.

**Table:**

Life stage	Relationship	LnSR	Exp‐Ctl
Gametes	Linear	NA	NA
**Embryos**	**Second order polynomial**	**1.08**	**2.95**
**Larvae**	**Second order polynomial**	**1.19**	**3.29**
Juveniles	Linear	NA	NA
Adults	Linear	NA	NA

Detection of non‐linearity in the class groups.

**Table:**

Class	Relationship	LnSR	Exp‐Ctl
Asteroidea	Linear	NA	NA
Echinoidea	Third order polynomial	NA*	NA
Holothuroidea	Linear	NA	NA
Ophiuroidea	Linear	NA	NA

Detection of non‐linearity in the region groups.

**Table:**

Region	Relationship	LnSR	Exp‐Ctl
**Tropical (4–23°)**	**Second order polynomial**	**1.36**	**3.89**
Sub–tropical (26–35°)	Linear	NA	NA
Temperate (36–53°)	Linear	NA	NA
Polar (62–78°)	Second order polynomial	NA*	NA*

## APPENDIX 7

### 7.1

The effect of warming above the predicted mean annual temperature (MAT) for the end of the century (+2.58°C, RCP8.5, 2081–2100; IPCC, 2019) on echinoderms, a comparison between biological responses (a, e), ontogenetic life stages (b, f), taxonomic classes (c, g) and regions (d, h). The mean effect sizes and the robust 95% confidence intervals are provided (left panels, a–d), as well as the raw data points (right panels, e–h). The number of responses in each group is provided in parentheses in the right panels. Significant mean effect sizes are indicated by an asterisk (**p* < .05; ***p* < .01; ****p* < .001) in the left panels. Mean effect sizes and 95% confidence intervals have been corrected for differences between the control and experimental temperatures (*LnSR*). Effect sizes above and below zero indicate a positive and negative response to warming, respectively.
