# Supplementary material for: Impacts of ocean warming on echinoderms: A meta‐analysis
Source: Ecol Evol. 2023 Aug 8;13(8):e10307. doi: 10.1002/ece3.10307 (PMC10409743; doi:10.1002/ece3.10307)
Supplement: Supplementary file 2 — File S2 [file ECE3-13-e10307-s001.html]

S2\_code.knit


# S2: Code for meta-analysis on the impacts of warming on echinoderms.

## Starting new session

```
rm(list=ls())
```

## Installing data

```
#pacman::p_load(devtools, tidyverse, metafor, patchwork, R.rsp)
#install.packages("metafor")
library(metafor)
```

```
## Loading required package: Matrix
```

```
## Loading required package: metadat
```

```
## Loading required package: numDeriv
```

```
## 
## Loading the 'metafor' package (version 4.2-0). For an
## introduction to the package please type: help(metafor)
```

```
#install.packages("RCurl")
library(RCurl)
#install.packages("bitops")
library(bitops)
```

```
## 
## Attaching package: 'bitops'
```

```
## The following object is masked from 'package:Matrix':
## 
##     %&%
```

```
#install.packages("Formula")
library(Formula)
#install.packages("tidyverse")
library(tidyverse)
```

```
## ── Attaching core tidyverse packages ──────────────────────── tidyverse 2.0.0 ──
## ✔ dplyr     1.1.2     ✔ readr     2.1.4
## ✔ forcats   1.0.0     ✔ stringr   1.5.0
## ✔ ggplot2   3.4.2     ✔ tibble    3.2.1
## ✔ lubridate 1.9.2     ✔ tidyr     1.3.0
## ✔ purrr     1.0.1
```

```
## ── Conflicts ────────────────────────────────────────── tidyverse_conflicts() ──
## ✖ tidyr::complete() masks RCurl::complete()
## ✖ tidyr::expand()   masks Matrix::expand()
## ✖ dplyr::filter()   masks stats::filter()
## ✖ dplyr::lag()      masks stats::lag()
## ✖ tidyr::pack()     masks Matrix::pack()
## ✖ tidyr::unpack()   masks Matrix::unpack()
## ℹ Use the conflicted package (<http://conflicted.r-lib.org/>) to force all conflicts to become errors
```

```
#install.packages("ggpubr")
library(ggpubr)
#install.packages("jtools")
library(jtools)
#install.packages("multcomp")
library(multcomp)
```

```
## Loading required package: mvtnorm
## 
## Attaching package: 'mvtnorm'
## 
## The following object is masked from 'package:jtools':
## 
##     standardize
## 
## Loading required package: survival
## Loading required package: TH.data
## Loading required package: MASS
## 
## Attaching package: 'MASS'
## 
## The following object is masked from 'package:dplyr':
## 
##     select
## 
## 
## Attaching package: 'TH.data'
## 
## The following object is masked from 'package:MASS':
## 
##     geyser
```

```
#install.packages("car")
library(car)
```

```
## Loading required package: carData
## 
## Attaching package: 'car'
## 
## The following object is masked from 'package:dplyr':
## 
##     recode
## 
## The following object is masked from 'package:purrr':
## 
##     some
## 
## The following object is masked from 'package:metafor':
## 
##     vif
```

```
#install.packages("clubSandwich")
library(clubSandwich)
```

```
## Registered S3 method overwritten by 'clubSandwich':
##   method    from    
##   bread.mlm sandwich
```

# Biological responses

## Biological responses (full model)

## Loading data

```
getwd()
```

```
## [1] "/Users/bethanlang/Documents/PhD/Literature Review/Docs Ecol & Evol submission"
```

```
setwd('/Users/bethanlang/Documents/PhD/Literature Review')
Traitcatdata <- read_csv("Lang et al. 2022_dataset_2906.csv")
```

```
## New names:
## Rows: 710 Columns: 50
## ── Column specification
## ──────────────────────────────────────────────────────── Delimiter: "," chr
## (9): Study_name, Species, Class, Lifestage, Latitude_cat, Latitude_cat2... dbl
## (40): Study_number, Year, Effect_size_id, lat...8, Response_number, Ctl_... lgl
## (1): Exp_temp_between_CC_MAT_WARM
## ℹ Use `spec()` to retrieve the full column specification for this data. ℹ
## Specify the column types or set `show_col_types = FALSE` to quiet this message.
## • `lat` -> `lat...8`
## • `Exp_temp` -> `Exp_temp...19`
## • `lat` -> `lat...35`
## • `Exp_temp` -> `Exp_temp...42`
```

## Turning random effects into factors

```
Traitcatdata$Study_number = factor(Traitcatdata$Study_number)
Traitcatdata$Response_number = factor(Traitcatdata$Response_number)
```

## Ordering data

```
Traitcatdata <- Traitcatdata[order(Traitcatdata$Trait_cat),]
```

## Number in each trait

```
Numbertraits <- Traitcatdata %>% group_by(Trait_cat) %>% summarise(n()) 
Numbertraits$n <- Numbertraits$`n()`
```

## The model

```
Traitcat_model <- rma.mv(yi = LnRR, V = Variance, mods = ~LnSR:Trait_cat-1, test="t", random = list(~1|Study_number/Response_number), method = "REML", data = Traitcatdata)
print(Traitcat_model)
```

```
## 
## Multivariate Meta-Analysis Model (k = 710; method: REML)
## 
## Variance Components:
## 
##             estim    sqrt  nlvls  fixed                        factor 
## sigma^2.1  0.0802  0.2832     85     no                  Study_number 
## sigma^2.2  0.1186  0.3444    396     no  Study_number/Response_number 
## 
## Test for Residual Heterogeneity:
## QE(df = 703) = 19754.3111, p-val < .0001
## 
## Test of Moderators (coefficients 1:7):
## F(df1 = 7, df2 = 703) = 66.5835, p-val < .0001
## 
## Model Results:
## 
##                                      estimate      se      tval   df    pval 
## LnSR:Trait_catDevelopment success     -0.1333  0.0128  -10.4258  703  <.0001 
## LnSR:Trait_catFeeding and nutrition   -0.1095  0.0189   -5.8067  703  <.0001 
## LnSR:Trait_catGrowth                  -0.0208  0.0143   -1.4561  703  0.1458 
## LnSR:Trait_catMetabolic rate           0.2806  0.0331    8.4712  703  <.0001 
## LnSR:Trait_catMovement                -0.0111  0.0208   -0.5330  703  0.5942 
## LnSR:Trait_catReproductive success    -0.0226  0.0070   -3.2109  703  0.0014 
## LnSR:Trait_catSurvival                -0.4274  0.0280  -15.2473  703  <.0001 
##                                        ci.lb    ci.ub      
## LnSR:Trait_catDevelopment success    -0.1584  -0.1082  *** 
## LnSR:Trait_catFeeding and nutrition  -0.1466  -0.0725  *** 
## LnSR:Trait_catGrowth                 -0.0489   0.0073      
## LnSR:Trait_catMetabolic rate          0.2156   0.3456  *** 
## LnSR:Trait_catMovement               -0.0520   0.0298      
## LnSR:Trait_catReproductive success   -0.0364  -0.0088   ** 
## LnSR:Trait_catSurvival               -0.4824  -0.3724  *** 
## 
## ---
## Signif. codes:  0 '***' 0.001 '**' 0.01 '*' 0.05 '.' 0.1 ' ' 1
```

## Robust model

```
Traitcat_model_robust <- robust(Traitcat_model, cluster=Traitcatdata$Study_number)
Traitcat_model_robust
```

```
## 
## Multivariate Meta-Analysis Model (k = 710; method: REML)
## 
## Variance Components:
## 
##             estim    sqrt  nlvls  fixed                        factor 
## sigma^2.1  0.0802  0.2832     85     no                  Study_number 
## sigma^2.2  0.1186  0.3444    396     no  Study_number/Response_number 
## 
## Test for Residual Heterogeneity:
## QE(df = 703) = 19754.3111, p-val < .0001
## 
## Number of estimates:   710
## Number of clusters:    85
## Estimates per cluster: 1-48 (mean: 8.35, median: 6)
## 
## Test of Moderators (coefficients 1:7):¹
## F(df1 = 7, df2 = 78) = 4.9996, p-val = 0.0001
## 
## Model Results:
## 
##                                      estimate      se¹     tval¹  df¹    pval¹ 
## LnSR:Trait_catDevelopment success     -0.1333  0.0992   -1.3436   78   0.1830  
## LnSR:Trait_catFeeding and nutrition   -0.1095  0.1410   -0.7769   78   0.4396  
## LnSR:Trait_catGrowth                  -0.0208  0.0334   -0.6236   78   0.5347  
## LnSR:Trait_catMetabolic rate           0.2806  0.1101    2.5494   78   0.0128  
## LnSR:Trait_catMovement                -0.0111  0.0580   -0.1915   78   0.8486  
## LnSR:Trait_catReproductive success    -0.0226  0.0128   -1.7639   78   0.0817  
## LnSR:Trait_catSurvival                -0.4274  0.1166   -3.6639   78   0.0005  
##                                        ci.lb¹    ci.ub¹      
## LnSR:Trait_catDevelopment success    -0.3308    0.0642       
## LnSR:Trait_catFeeding and nutrition  -0.3902    0.1712       
## LnSR:Trait_catGrowth                 -0.0873    0.0456       
## LnSR:Trait_catMetabolic rate          0.0615    0.4997     * 
## LnSR:Trait_catMovement               -0.1265    0.1043       
## LnSR:Trait_catReproductive success   -0.0481    0.0029     . 
## LnSR:Trait_catSurvival               -0.6596   -0.1952   *** 
## 
## ---
## Signif. codes:  0 '***' 0.001 '**' 0.01 '*' 0.05 '.' 0.1 ' ' 1
## 
## 1) results based on cluster-robust inference (var-cov estimator: CR1,
##    approx t/F-tests and confidence intervals, df: residual method)
```

## Model fit

```
res <- resid(Traitcat_model_robust)
plot(fitted(Traitcat_model_robust), res)
abline(0,0)
```

```
qqnorm(res)
qqline(res)
```

```
plot(density(res))
```

## Overall Publication bias

```
all <- trimfill(rma(LnRR, Variance, data = subset(Traitcatdata)),"left")$k0 
all2 <- trimfill(rma(LnRR, Variance, data = subset(Traitcatdata)),"right")$k0 
all
```

```
## [1] 168
```

```
all2
```

```
## [1] 0
```

## Sensitivity analysis

### Influential data points

```
Traitcatdata2 <- Traitcatdata %>%
  arrange(LnRR)
```

```
datest_traitcat <- Traitcatdata2[-1,]
modeltest_traitcat <- rma.mv(yi = LnRR, V = Variance, mods = ~LnSR:Trait_cat - 1, test="t", random = list(~1|Study_number/Response_number), method = "REML", data = datest_traitcat)
Traitcat_model_robust2 <- robust(modeltest_traitcat, cluster=datest_traitcat$Study_number)
Traitcat_model_robust2
```

```
## 
## Multivariate Meta-Analysis Model (k = 709; method: REML)
## 
## Variance Components:
## 
##             estim    sqrt  nlvls  fixed                        factor 
## sigma^2.1  0.0803  0.2833     85     no                  Study_number 
## sigma^2.2  0.1186  0.3443    396     no  Study_number/Response_number 
## 
## Test for Residual Heterogeneity:
## QE(df = 702) = 19515.0923, p-val < .0001
## 
## Number of estimates:   709
## Number of clusters:    85
## Estimates per cluster: 1-48 (mean: 8.34, median: 6)
## 
## Test of Moderators (coefficients 1:7):¹
## F(df1 = 7, df2 = 78) = 4.9978, p-val = 0.0001
## 
## Model Results:
## 
##                                      estimate      se¹     tval¹  df¹    pval¹ 
## LnSR:Trait_catDevelopment success     -0.1288  0.0985   -1.3074   78   0.1949  
## LnSR:Trait_catFeeding and nutrition   -0.1095  0.1410   -0.7768   78   0.4396  
## LnSR:Trait_catGrowth                  -0.0208  0.0334   -0.6227   78   0.5353  
## LnSR:Trait_catMetabolic rate           0.2806  0.1101    2.5492   78   0.0128  
## LnSR:Trait_catMovement                -0.0111  0.0580   -0.1915   78   0.8486  
## LnSR:Trait_catReproductive success    -0.0226  0.0128   -1.7633   78   0.0818  
## LnSR:Trait_catSurvival                -0.4273  0.1166   -3.6643   78   0.0005  
##                                        ci.lb¹    ci.ub¹      
## LnSR:Trait_catDevelopment success    -0.3249    0.0673       
## LnSR:Trait_catFeeding and nutrition  -0.3902    0.1712       
## LnSR:Trait_catGrowth                 -0.0872    0.0457       
## LnSR:Trait_catMetabolic rate          0.0615    0.4997     * 
## LnSR:Trait_catMovement               -0.1265    0.1043       
## LnSR:Trait_catReproductive success   -0.0481    0.0029     . 
## LnSR:Trait_catSurvival               -0.6594   -0.1951   *** 
## 
## ---
## Signif. codes:  0 '***' 0.001 '**' 0.01 '*' 0.05 '.' 0.1 ' ' 1
## 
## 1) results based on cluster-robust inference (var-cov estimator: CR1,
##    approx t/F-tests and confidence intervals, df: residual method)
```

```
datest_traitcat2 <- datest_traitcat[-1,]
modeltest_traitcat2 <- rma.mv(yi = LnRR, V = Variance, mods = ~LnSR:Trait_cat - 1, test="t", random = list(~1 |Study_number/Response_number), method = "REML", data = datest_traitcat2)
Traitcat_model_robust3 <- robust(modeltest_traitcat2, cluster=datest_traitcat2$Study_number)
Traitcat_model_robust3
```

```
## 
## Multivariate Meta-Analysis Model (k = 708; method: REML)
## 
## Variance Components:
## 
##             estim    sqrt  nlvls  fixed                        factor 
## sigma^2.1  0.0805  0.2837     85     no                  Study_number 
## sigma^2.2  0.1183  0.3440    396     no  Study_number/Response_number 
## 
## Test for Residual Heterogeneity:
## QE(df = 701) = 19479.3103, p-val < .0001
## 
## Number of estimates:   708
## Number of clusters:    85
## Estimates per cluster: 1-48 (mean: 8.33, median: 6)
## 
## Test of Moderators (coefficients 1:7):¹
## F(df1 = 7, df2 = 78) = 4.9976, p-val = 0.0001
## 
## Model Results:
## 
##                                      estimate      se¹     tval¹  df¹    pval¹ 
## LnSR:Trait_catDevelopment success     -0.1281  0.0984   -1.3016   78   0.1969  
## LnSR:Trait_catFeeding and nutrition   -0.1095  0.1410   -0.7767   78   0.4397  
## LnSR:Trait_catGrowth                  -0.0208  0.0334   -0.6228   78   0.5353  
## LnSR:Trait_catMetabolic rate           0.2805  0.1101    2.5476   78   0.0128  
## LnSR:Trait_catMovement                -0.0111  0.0580   -0.1916   78   0.8485  
## LnSR:Trait_catReproductive success    -0.0226  0.0128   -1.7645   78   0.0816  
## LnSR:Trait_catSurvival                -0.4273  0.1166   -3.6647   78   0.0004  
##                                        ci.lb¹    ci.ub¹      
## LnSR:Trait_catDevelopment success    -0.3241    0.0678       
## LnSR:Trait_catFeeding and nutrition  -0.3901    0.1711       
## LnSR:Trait_catGrowth                 -0.0872    0.0457       
## LnSR:Trait_catMetabolic rate          0.0613    0.4996     * 
## LnSR:Trait_catMovement               -0.1266    0.1043       
## LnSR:Trait_catReproductive success   -0.0481    0.0029     . 
## LnSR:Trait_catSurvival               -0.6594   -0.1951   *** 
## 
## ---
## Signif. codes:  0 '***' 0.001 '**' 0.01 '*' 0.05 '.' 0.1 ' ' 1
## 
## 1) results based on cluster-robust inference (var-cov estimator: CR1,
##    approx t/F-tests and confidence intervals, df: residual method)
```

```
datest_traitcat3 <- datest_traitcat2[-1,]
modeltest_traitcat3 <- rma.mv(yi = LnRR, V = Variance, mods = ~LnSR:Trait_cat - 1, test="t", random = list(~1 |Study_number/Response_number), method = "REML", data = datest_traitcat3)
Traitcat_model_robust4 <- robust(modeltest_traitcat3, cluster=datest_traitcat3$Study_number)
Traitcat_model_robust4
```

```
## 
## Multivariate Meta-Analysis Model (k = 707; method: REML)
## 
## Variance Components:
## 
##             estim    sqrt  nlvls  fixed                        factor 
## sigma^2.1  0.0805  0.2838     85     no                  Study_number 
## sigma^2.2  0.1183  0.3440    396     no  Study_number/Response_number 
## 
## Test for Residual Heterogeneity:
## QE(df = 700) = 19458.6998, p-val < .0001
## 
## Number of estimates:   707
## Number of clusters:    85
## Estimates per cluster: 1-48 (mean: 8.32, median: 6)
## 
## Test of Moderators (coefficients 1:7):¹
## F(df1 = 7, df2 = 78) = 4.9975, p-val = 0.0001
## 
## Model Results:
## 
##                                      estimate      se¹     tval¹  df¹    pval¹ 
## LnSR:Trait_catDevelopment success     -0.1276  0.0984   -1.2974   78   0.1983  
## LnSR:Trait_catFeeding and nutrition   -0.1095  0.1410   -0.7767   78   0.4397  
## LnSR:Trait_catGrowth                  -0.0208  0.0334   -0.6227   78   0.5353  
## LnSR:Trait_catMetabolic rate           0.2805  0.1101    2.5476   78   0.0128  
## LnSR:Trait_catMovement                -0.0111  0.0580   -0.1916   78   0.8485  
## LnSR:Trait_catReproductive success    -0.0226  0.0128   -1.7644   78   0.0816  
## LnSR:Trait_catSurvival                -0.4272  0.1166   -3.6647   78   0.0004  
##                                        ci.lb¹    ci.ub¹      
## LnSR:Trait_catDevelopment success    -0.3235    0.0682       
## LnSR:Trait_catFeeding and nutrition  -0.3901    0.1711       
## LnSR:Trait_catGrowth                 -0.0872    0.0457       
## LnSR:Trait_catMetabolic rate          0.0613    0.4996     * 
## LnSR:Trait_catMovement               -0.1266    0.1043       
## LnSR:Trait_catReproductive success   -0.0481    0.0029     . 
## LnSR:Trait_catSurvival               -0.6593   -0.1951   *** 
## 
## ---
## Signif. codes:  0 '***' 0.001 '**' 0.01 '*' 0.05 '.' 0.1 ' ' 1
## 
## 1) results based on cluster-robust inference (var-cov estimator: CR1,
##    approx t/F-tests and confidence intervals, df: residual method)
```

```
datest_traitcat4 <- datest_traitcat3[-1,]
modeltest_traitcat4 <- rma.mv(yi = LnRR, V = Variance, mods = ~LnSR:Trait_cat - 1, test="t", random = list(~1 |Study_number/Response_number), method = "REML", data = datest_traitcat4)
Traitcat_model_robust5 <- robust(modeltest_traitcat4, cluster=datest_traitcat4$Study_number)
Traitcat_model_robust5
```

```
## 
## Multivariate Meta-Analysis Model (k = 706; method: REML)
## 
## Variance Components:
## 
##             estim    sqrt  nlvls  fixed                        factor 
## sigma^2.1  0.0805  0.2838     85     no                  Study_number 
## sigma^2.2  0.1186  0.3444    396     no  Study_number/Response_number 
## 
## Test for Residual Heterogeneity:
## QE(df = 699) = 19448.0622, p-val < .0001
## 
## Number of estimates:   706
## Number of clusters:    85
## Estimates per cluster: 1-48 (mean: 8.31, median: 6)
## 
## Test of Moderators (coefficients 1:7):¹
## F(df1 = 7, df2 = 78) = 4.9981, p-val = 0.0001
## 
## Model Results:
## 
##                                      estimate      se¹     tval¹  df¹    pval¹ 
## LnSR:Trait_catDevelopment success     -0.1273  0.0984   -1.2943   78   0.1994  
## LnSR:Trait_catFeeding and nutrition   -0.1095  0.1410   -0.7768   78   0.4396  
## LnSR:Trait_catGrowth                  -0.0208  0.0334   -0.6227   78   0.5353  
## LnSR:Trait_catMetabolic rate           0.2806  0.1101    2.5489   78   0.0128  
## LnSR:Trait_catMovement                -0.0111  0.0580   -0.1915   78   0.8486  
## LnSR:Trait_catReproductive success    -0.0226  0.0128   -1.7641   78   0.0816  
## LnSR:Trait_catSurvival                -0.4273  0.1166   -3.6642   78   0.0005  
##                                        ci.lb¹    ci.ub¹      
## LnSR:Trait_catDevelopment success    -0.3232    0.0685       
## LnSR:Trait_catFeeding and nutrition  -0.3903    0.1712       
## LnSR:Trait_catGrowth                 -0.0872    0.0457       
## LnSR:Trait_catMetabolic rate          0.0614    0.4997     * 
## LnSR:Trait_catMovement               -0.1265    0.1043       
## LnSR:Trait_catReproductive success   -0.0481    0.0029     . 
## LnSR:Trait_catSurvival               -0.6594   -0.1951   *** 
## 
## ---
## Signif. codes:  0 '***' 0.001 '**' 0.01 '*' 0.05 '.' 0.1 ' ' 1
## 
## 1) results based on cluster-robust inference (var-cov estimator: CR1,
##    approx t/F-tests and confidence intervals, df: residual method)
```

```
datest_traitcat5 <- datest_traitcat4[-1,]
modeltest_traitcat5 <- rma.mv(yi = LnRR, V = Variance, mods = ~LnSR:Trait_cat - 1, test="t", random = list(~1 |Study_number/Response_number), method = "REML", data = datest_traitcat5)
Traitcat_model_robust6 <- robust(modeltest_traitcat5, cluster=datest_traitcat5$Study_number)
Traitcat_model_robust6
```

```
## 
## Multivariate Meta-Analysis Model (k = 705; method: REML)
## 
## Variance Components:
## 
##             estim    sqrt  nlvls  fixed                        factor 
## sigma^2.1  0.0805  0.2838     85     no                  Study_number 
## sigma^2.2  0.1185  0.3443    396     no  Study_number/Response_number 
## 
## Test for Residual Heterogeneity:
## QE(df = 698) = 19432.2453, p-val < .0001
## 
## Number of estimates:   705
## Number of clusters:    85
## Estimates per cluster: 1-48 (mean: 8.29, median: 6)
## 
## Test of Moderators (coefficients 1:7):¹
## F(df1 = 7, df2 = 78) = 4.9990, p-val = 0.0001
## 
## Model Results:
## 
##                                      estimate      se¹     tval¹  df¹    pval¹ 
## LnSR:Trait_catDevelopment success     -0.1273  0.0984   -1.2942   78   0.1994  
## LnSR:Trait_catFeeding and nutrition   -0.1095  0.1410   -0.7768   78   0.4396  
## LnSR:Trait_catGrowth                  -0.0208  0.0334   -0.6220   78   0.5357  
## LnSR:Trait_catMetabolic rate           0.2806  0.1101    2.5486   78   0.0128  
## LnSR:Trait_catMovement                -0.0111  0.0580   -0.1915   78   0.8486  
## LnSR:Trait_catReproductive success    -0.0226  0.0128   -1.7640   78   0.0816  
## LnSR:Trait_catSurvival                -0.4245  0.1160   -3.6609   78   0.0005  
##                                        ci.lb¹    ci.ub¹      
## LnSR:Trait_catDevelopment success    -0.3232    0.0685       
## LnSR:Trait_catFeeding and nutrition  -0.3902    0.1712       
## LnSR:Trait_catGrowth                 -0.0872    0.0457       
## LnSR:Trait_catMetabolic rate          0.0614    0.4997     * 
## LnSR:Trait_catMovement               -0.1265    0.1043       
## LnSR:Trait_catReproductive success   -0.0481    0.0029     . 
## LnSR:Trait_catSurvival               -0.6554   -0.1937   *** 
## 
## ---
## Signif. codes:  0 '***' 0.001 '**' 0.01 '*' 0.05 '.' 0.1 ' ' 1
## 
## 1) results based on cluster-robust inference (var-cov estimator: CR1,
##    approx t/F-tests and confidence intervals, df: residual method)
```

```
datest_traitcat6 <- datest_traitcat5[-1,]
modeltest_traitcat6 <- rma.mv(yi = LnRR, V = Variance, mods = ~LnSR:Trait_cat - 1, test="t", random = list(~1 |Study_number/Response_number), method = "REML", data = datest_traitcat6)
Traitcat_model_robust7 <- robust(modeltest_traitcat6, cluster=datest_traitcat6$Study_number)
Traitcat_model_robust7
```

```
## 
## Multivariate Meta-Analysis Model (k = 704; method: REML)
## 
## Variance Components:
## 
##             estim    sqrt  nlvls  fixed                        factor 
## sigma^2.1  0.0683  0.2613     85     no                  Study_number 
## sigma^2.2  0.1193  0.3454    395     no  Study_number/Response_number 
## 
## Test for Residual Heterogeneity:
## QE(df = 697) = 19376.8141, p-val < .0001
## 
## Number of estimates:   704
## Number of clusters:    85
## Estimates per cluster: 1-48 (mean: 8.28, median: 6)
## 
## Test of Moderators (coefficients 1:7):¹
## F(df1 = 7, df2 = 78) = 5.0203, p-val < .0001
## 
## Model Results:
## 
##                                      estimate      se¹     tval¹  df¹    pval¹ 
## LnSR:Trait_catDevelopment success     -0.1278  0.0984   -1.2992   78   0.1977  
## LnSR:Trait_catFeeding and nutrition   -0.1086  0.1404   -0.7735   78   0.4416  
## LnSR:Trait_catGrowth                  -0.0201  0.0331   -0.6081   78   0.5449  
## LnSR:Trait_catMetabolic rate           0.2815  0.1091    2.5801   78   0.0118  
## LnSR:Trait_catMovement                -0.0111  0.0580   -0.1910   78   0.8490  
## LnSR:Trait_catReproductive success    -0.0225  0.0128   -1.7538   78   0.0834  
## LnSR:Trait_catSurvival                -0.4201  0.1147   -3.6626   78   0.0005  
##                                        ci.lb¹    ci.ub¹      
## LnSR:Trait_catDevelopment success    -0.3236    0.0680       
## LnSR:Trait_catFeeding and nutrition  -0.3881    0.1709       
## LnSR:Trait_catGrowth                 -0.0861    0.0458       
## LnSR:Trait_catMetabolic rate          0.0643    0.4988     * 
## LnSR:Trait_catMovement               -0.1265    0.1043       
## LnSR:Trait_catReproductive success   -0.0481    0.0030     . 
## LnSR:Trait_catSurvival               -0.6484   -0.1917   *** 
## 
## ---
## Signif. codes:  0 '***' 0.001 '**' 0.01 '*' 0.05 '.' 0.1 ' ' 1
## 
## 1) results based on cluster-robust inference (var-cov estimator: CR1,
##    approx t/F-tests and confidence intervals, df: residual method)
```

```
datest_traitcat7 <- datest_traitcat6[-1,]
modeltest_traitcat7 <- rma.mv(yi = LnRR, V = Variance, mods = ~LnSR:Trait_cat - 1, test="t", random = list(~1 |Study_number/Response_number), method = "REML", data = datest_traitcat7)
Traitcat_model_robust8 <- robust(modeltest_traitcat7, cluster=datest_traitcat7$Study_number)
Traitcat_model_robust8
```

```
## 
## Multivariate Meta-Analysis Model (k = 703; method: REML)
## 
## Variance Components:
## 
##             estim    sqrt  nlvls  fixed                        factor 
## sigma^2.1  0.0683  0.2614     85     no                  Study_number 
## sigma^2.2  0.1193  0.3454    395     no  Study_number/Response_number 
## 
## Test for Residual Heterogeneity:
## QE(df = 696) = 19226.9038, p-val < .0001
## 
## Number of estimates:   703
## Number of clusters:    85
## Estimates per cluster: 1-48 (mean: 8.27, median: 6)
## 
## Test of Moderators (coefficients 1:7):¹
## F(df1 = 7, df2 = 78) = 5.0199, p-val < .0001
## 
## Model Results:
## 
##                                      estimate      se¹     tval¹  df¹    pval¹ 
## LnSR:Trait_catDevelopment success     -0.1245  0.0991   -1.2563   78   0.2128  
## LnSR:Trait_catFeeding and nutrition   -0.1086  0.1404   -0.7734   78   0.4416  
## LnSR:Trait_catGrowth                  -0.0201  0.0331   -0.6075   78   0.5453  
## LnSR:Trait_catMetabolic rate           0.2815  0.1091    2.5800   78   0.0118  
## LnSR:Trait_catMovement                -0.0111  0.0580   -0.1910   78   0.8490  
## LnSR:Trait_catReproductive success    -0.0225  0.0128   -1.7535   78   0.0834  
## LnSR:Trait_catSurvival                -0.4200  0.1147   -3.6628   78   0.0005  
##                                        ci.lb¹    ci.ub¹      
## LnSR:Trait_catDevelopment success    -0.3218    0.0728       
## LnSR:Trait_catFeeding and nutrition  -0.3881    0.1709       
## LnSR:Trait_catGrowth                 -0.0861    0.0458       
## LnSR:Trait_catMetabolic rate          0.0643    0.4988     * 
## LnSR:Trait_catMovement               -0.1265    0.1043       
## LnSR:Trait_catReproductive success   -0.0481    0.0030     . 
## LnSR:Trait_catSurvival               -0.6483   -0.1917   *** 
## 
## ---
## Signif. codes:  0 '***' 0.001 '**' 0.01 '*' 0.05 '.' 0.1 ' ' 1
## 
## 1) results based on cluster-robust inference (var-cov estimator: CR1,
##    approx t/F-tests and confidence intervals, df: residual method)
```

```
datest_traitcat8 <- datest_traitcat7[-1,]
modeltest_traitcat8 <- rma.mv(yi = LnRR, V = Variance, mods = ~LnSR:Trait_cat - 1, test="t", random = list(~1 |Study_number/Response_number), method = "REML", data = datest_traitcat8)
Traitcat_model_robust9 <- robust(modeltest_traitcat8, cluster=datest_traitcat8$Study_number)
Traitcat_model_robust9
```

```
## 
## Multivariate Meta-Analysis Model (k = 702; method: REML)
## 
## Variance Components:
## 
##             estim    sqrt  nlvls  fixed                        factor 
## sigma^2.1  0.0619  0.2488     85     no                  Study_number 
## sigma^2.2  0.1165  0.3413    394     no  Study_number/Response_number 
## 
## Test for Residual Heterogeneity:
## QE(df = 695) = 19173.9759, p-val < .0001
## 
## Number of estimates:   702
## Number of clusters:    85
## Estimates per cluster: 1-48 (mean: 8.26, median: 6)
## 
## Test of Moderators (coefficients 1:7):¹
## F(df1 = 7, df2 = 78) = 5.0156, p-val < .0001
## 
## Model Results:
## 
##                                      estimate      se¹     tval¹  df¹    pval¹ 
## LnSR:Trait_catDevelopment success     -0.1233  0.0989   -1.2474   78   0.2160  
## LnSR:Trait_catFeeding and nutrition   -0.1074  0.1394   -0.7703   78   0.4434  
## LnSR:Trait_catGrowth                  -0.0197  0.0330   -0.5981   78   0.5515  
## LnSR:Trait_catMetabolic rate           0.2806  0.1086    2.5826   78   0.0117  
## LnSR:Trait_catMovement                -0.0111  0.0579   -0.1919   78   0.8483  
## LnSR:Trait_catReproductive success    -0.0225  0.0128   -1.7509   78   0.0839  
## LnSR:Trait_catSurvival                -0.4214  0.1148   -3.6712   78   0.0004  
##                                        ci.lb¹    ci.ub¹      
## LnSR:Trait_catDevelopment success    -0.3201    0.0735       
## LnSR:Trait_catFeeding and nutrition  -0.3848    0.1701       
## LnSR:Trait_catGrowth                 -0.0853    0.0459       
## LnSR:Trait_catMetabolic rate          0.0643    0.4968     * 
## LnSR:Trait_catMovement               -0.1264    0.1042       
## LnSR:Trait_catReproductive success   -0.0480    0.0031     . 
## LnSR:Trait_catSurvival               -0.6500   -0.1929   *** 
## 
## ---
## Signif. codes:  0 '***' 0.001 '**' 0.01 '*' 0.05 '.' 0.1 ' ' 1
## 
## 1) results based on cluster-robust inference (var-cov estimator: CR1,
##    approx t/F-tests and confidence intervals, df: residual method)
```

```
datest_traitcat9 <- datest_traitcat8[-1,]
modeltest_traitcat9 <- rma.mv(yi = LnRR, V = Variance, mods = ~LnSR:Trait_cat - 1, test="t", random = list(~1 |Study_number/Response_number), method = "REML", data = datest_traitcat9)
Traitcat_model_robust10 <- robust(modeltest_traitcat9, cluster=datest_traitcat9$Study_number)
Traitcat_model_robust10
```

```
## 
## Multivariate Meta-Analysis Model (k = 701; method: REML)
## 
## Variance Components:
## 
##             estim    sqrt  nlvls  fixed                        factor 
## sigma^2.1  0.0612  0.2473     85     no                  Study_number 
## sigma^2.2  0.1163  0.3410    394     no  Study_number/Response_number 
## 
## Test for Residual Heterogeneity:
## QE(df = 694) = 19142.0454, p-val < .0001
## 
## Number of estimates:   701
## Number of clusters:    85
## Estimates per cluster: 1-48 (mean: 8.25, median: 6)
## 
## Test of Moderators (coefficients 1:7):¹
## F(df1 = 7, df2 = 78) = 5.0182, p-val < .0001
## 
## Model Results:
## 
##                                      estimate      se¹     tval¹  df¹    pval¹ 
## LnSR:Trait_catDevelopment success     -0.1228  0.0988   -1.2433   78   0.2175  
## LnSR:Trait_catFeeding and nutrition   -0.1072  0.1393   -0.7699   78   0.4437  
## LnSR:Trait_catGrowth                  -0.0197  0.0329   -0.5968   78   0.5523  
## LnSR:Trait_catMetabolic rate           0.2805  0.1086    2.5838   78   0.0116  
## LnSR:Trait_catMovement                -0.0111  0.0579   -0.1919   78   0.8483  
## LnSR:Trait_catReproductive success    -0.0225  0.0128   -1.7522   78   0.0837  
## LnSR:Trait_catSurvival                -0.4214  0.1147   -3.6722   78   0.0004  
##                                        ci.lb¹    ci.ub¹      
## LnSR:Trait_catDevelopment success    -0.3195    0.0738       
## LnSR:Trait_catFeeding and nutrition  -0.3845    0.1701       
## LnSR:Trait_catGrowth                 -0.0852    0.0459       
## LnSR:Trait_catMetabolic rate          0.0644    0.4967     * 
## LnSR:Trait_catMovement               -0.1264    0.1042       
## LnSR:Trait_catReproductive success   -0.0481    0.0031     . 
## LnSR:Trait_catSurvival               -0.6498   -0.1929   *** 
## 
## ---
## Signif. codes:  0 '***' 0.001 '**' 0.01 '*' 0.05 '.' 0.1 ' ' 1
## 
## 1) results based on cluster-robust inference (var-cov estimator: CR1,
##    approx t/F-tests and confidence intervals, df: residual method)
```

```
datest_traitcat10 <- datest_traitcat9[-701,]
modeltest_traitcat10 <- rma.mv(yi = LnRR, V = Variance, mods = ~LnSR:Trait_cat - 1, test="t", random = list(~1 |Study_number/Response_number), method = "REML", data = datest_traitcat10)
Traitcat_model_robust11 <- robust(modeltest_traitcat10, cluster=datest_traitcat10$Study_number)
Traitcat_model_robust11
```

```
## 
## Multivariate Meta-Analysis Model (k = 700; method: REML)
## 
## Variance Components:
## 
##             estim    sqrt  nlvls  fixed                        factor 
## sigma^2.1  0.0597  0.2442     85     no                  Study_number 
## sigma^2.2  0.1162  0.3409    394     no  Study_number/Response_number 
## 
## Test for Residual Heterogeneity:
## QE(df = 693) = 19134.8349, p-val < .0001
## 
## Number of estimates:   700
## Number of clusters:    85
## Estimates per cluster: 1-48 (mean: 8.24, median: 6)
## 
## Test of Moderators (coefficients 1:7):¹
## F(df1 = 7, df2 = 78) = 5.0272, p-val < .0001
## 
## Model Results:
## 
##                                      estimate      se¹     tval¹  df¹    pval¹ 
## LnSR:Trait_catDevelopment success     -0.1228  0.0988   -1.2437   78   0.2173  
## LnSR:Trait_catFeeding and nutrition   -0.1070  0.1391   -0.7693   78   0.4440  
## LnSR:Trait_catGrowth                  -0.0194  0.0329   -0.5916   78   0.5558  
## LnSR:Trait_catMetabolic rate           0.2806  0.1084    2.5879   78   0.0115  
## LnSR:Trait_catMovement                -0.0111  0.0579   -0.1919   78   0.8484  
## LnSR:Trait_catReproductive success    -0.0225  0.0128   -1.7560   78   0.0830  
## LnSR:Trait_catSurvival                -0.4213  0.1147   -3.6734   78   0.0004  
##                                        ci.lb¹    ci.ub¹      
## LnSR:Trait_catDevelopment success    -0.3194    0.0738       
## LnSR:Trait_catFeeding and nutrition  -0.3841    0.1700       
## LnSR:Trait_catGrowth                 -0.0849    0.0460       
## LnSR:Trait_catMetabolic rate          0.0647    0.4964     * 
## LnSR:Trait_catMovement               -0.1264    0.1042       
## LnSR:Trait_catReproductive success   -0.0481    0.0030     . 
## LnSR:Trait_catSurvival               -0.6496   -0.1930   *** 
## 
## ---
## Signif. codes:  0 '***' 0.001 '**' 0.01 '*' 0.05 '.' 0.1 ' ' 1
## 
## 1) results based on cluster-robust inference (var-cov estimator: CR1,
##    approx t/F-tests and confidence intervals, df: residual method)
```

### Removing papers that contribute with 2 or more studies.

```
More1study_traitcat <- Traitcatdata %>% group_by(Study_name) %>% summarise(unique(Study_number)) %>% summarise(n())
```

```
## Warning: Returning more (or less) than 1 row per `summarise()` group was deprecated in
## dplyr 1.1.0.
## ℹ Please use `reframe()` instead.
## ℹ When switching from `summarise()` to `reframe()`, remember that `reframe()`
##   always returns an ungrouped data frame and adjust accordingly.
## Call `lifecycle::last_lifecycle_warnings()` to see where this warning was
## generated.
```

```
## `summarise()` has grouped output by 'Study_name'. You can override using the
## `.groups` argument.
```

```
More1study_traitcat$n <- More1study_traitcat$`n()`
subset(More1study_traitcat, n >1)
```

```
## # A tibble: 6 × 3
##   Study_name                     `n()`     n
##   <chr>                          <int> <int>
## 1 Benitez Villalobos et al. 2006     3     3
## 2 Detree et al. 2020                 2     2
## 3 Garcia et al. 2018                 4     4
## 4 Hill and Lawrence 2006             2     2
## 5 Kuhnhold et al. 2019               2     2
## 6 Rupp 1973                          6     6
```

```
Rem_Rupp_traitcat <- subset(Traitcatdata, Study_name !="Rupp 1973")
model_rem_Rupp_traitcat <- rma.mv(yi = LnRR, V = Variance, mods = ~LnSR:Trait_cat - 1, test="t", random = list(~1 |Study_number/Response_number), method = "REML", data = Rem_Rupp_traitcat)
Traitcat_model_robust12 <- robust(model_rem_Rupp_traitcat, cluster=Rem_Rupp_traitcat$Study_number)
Traitcat_model_robust12
```

```
## 
## Multivariate Meta-Analysis Model (k = 682; method: REML)
## 
## Variance Components:
## 
##             estim    sqrt  nlvls  fixed                        factor 
## sigma^2.1  0.0852  0.2919     79     no                  Study_number 
## sigma^2.2  0.1219  0.3491    386     no  Study_number/Response_number 
## 
## Test for Residual Heterogeneity:
## QE(df = 675) = 18268.4045, p-val < .0001
## 
## Number of estimates:   682
## Number of clusters:    79
## Estimates per cluster: 1-48 (mean: 8.63, median: 6)
## 
## Test of Moderators (coefficients 1:7):¹
## F(df1 = 7, df2 = 72) = 4.2054, p-val = 0.0006
## 
## Model Results:
## 
##                                      estimate      se¹     tval¹  df¹    pval¹ 
## LnSR:Trait_catDevelopment success     -0.1706  0.1374   -1.2421   72   0.2182  
## LnSR:Trait_catFeeding and nutrition   -0.1100  0.1424   -0.7728   72   0.4422  
## LnSR:Trait_catGrowth                  -0.0211  0.0337   -0.6265   72   0.5330  
## LnSR:Trait_catMetabolic rate           0.2823  0.1105    2.5560   72   0.0127  
## LnSR:Trait_catMovement                -0.0110  0.0582   -0.1886   72   0.8510  
## LnSR:Trait_catReproductive success    -0.0024  0.0259   -0.0930   72   0.9262  
## LnSR:Trait_catSurvival                -0.4283  0.1177   -3.6391   72   0.0005  
##                                        ci.lb¹    ci.ub¹      
## LnSR:Trait_catDevelopment success    -0.4445    0.1032       
## LnSR:Trait_catFeeding and nutrition  -0.3938    0.1738       
## LnSR:Trait_catGrowth                 -0.0884    0.0461       
## LnSR:Trait_catMetabolic rate          0.0621    0.5025     * 
## LnSR:Trait_catMovement               -0.1270    0.1050       
## LnSR:Trait_catReproductive success   -0.0540    0.0492       
## LnSR:Trait_catSurvival               -0.6630   -0.1937   *** 
## 
## ---
## Signif. codes:  0 '***' 0.001 '**' 0.01 '*' 0.05 '.' 0.1 ' ' 1
## 
## 1) results based on cluster-robust inference (var-cov estimator: CR1,
##    approx t/F-tests and confidence intervals, df: residual method)
```

```
Rem_Garcia_traitcat <- subset(Traitcatdata, Study_name !="Garcia et al. 2018")
model_rem_Garcia_traitcat <- rma.mv(yi = LnRR, V = Variance, mods = ~LnSR:Trait_cat - 1, test="t", random = list(~1 |Study_number/Response_number), method = "REML", data = Rem_Garcia_traitcat)
Traitcat_model_robust13 <- robust(model_rem_Garcia_traitcat, cluster=Rem_Garcia_traitcat$Study_number)
Traitcat_model_robust13
```

```
## 
## Multivariate Meta-Analysis Model (k = 689; method: REML)
## 
## Variance Components:
## 
##             estim    sqrt  nlvls  fixed                        factor 
## sigma^2.1  0.0861  0.2934     81     no                  Study_number 
## sigma^2.2  0.1184  0.3441    385     no  Study_number/Response_number 
## 
## Test for Residual Heterogeneity:
## QE(df = 682) = 19409.2965, p-val < .0001
## 
## Number of estimates:   689
## Number of clusters:    81
## Estimates per cluster: 1-48 (mean: 8.51, median: 6)
## 
## Test of Moderators (coefficients 1:7):¹
## F(df1 = 7, df2 = 74) = 6.0117, p-val < .0001
## 
## Model Results:
## 
##                                      estimate      se¹     tval¹  df¹    pval¹ 
## LnSR:Trait_catDevelopment success     -0.1330  0.0994   -1.3372   74   0.1852  
## LnSR:Trait_catFeeding and nutrition   -0.1099  0.1416   -0.7765   74   0.4400  
## LnSR:Trait_catGrowth                  -0.0204  0.0334   -0.6114   74   0.5428  
## LnSR:Trait_catMetabolic rate           0.2803  0.1107    2.5321   74   0.0135  
## LnSR:Trait_catMovement                -0.0111  0.0581   -0.1912   74   0.8489  
## LnSR:Trait_catReproductive success    -0.0239  0.0140   -1.7111   74   0.0913  
## LnSR:Trait_catSurvival                -0.3582  0.0784   -4.5710   74   <.0001  
##                                        ci.lb¹    ci.ub¹      
## LnSR:Trait_catDevelopment success    -0.3311    0.0652       
## LnSR:Trait_catFeeding and nutrition  -0.3920    0.1722       
## LnSR:Trait_catGrowth                 -0.0870    0.0461       
## LnSR:Trait_catMetabolic rate          0.0597    0.5009     * 
## LnSR:Trait_catMovement               -0.1269    0.1047       
## LnSR:Trait_catReproductive success   -0.0518    0.0039     . 
## LnSR:Trait_catSurvival               -0.5143   -0.2020   *** 
## 
## ---
## Signif. codes:  0 '***' 0.001 '**' 0.01 '*' 0.05 '.' 0.1 ' ' 1
## 
## 1) results based on cluster-robust inference (var-cov estimator: CR1,
##    approx t/F-tests and confidence intervals, df: residual method)
```

```
Rem_Villalobos_traitcat <- subset(Traitcatdata, Study_name !="Villalobos et al. 2006")
model_rem_Villalobos_traitcat <- rma.mv(yi = LnRR, V = Variance, mods = ~LnSR:Trait_cat - 1, test="t", random = list(~1 |Study_number/Response_number), method = "REML", data = Rem_Villalobos_traitcat)
Traitcat_model_robust14 <- robust(model_rem_Villalobos_traitcat, cluster=Rem_Villalobos_traitcat$Study_number)
Traitcat_model_robust14
```

```
## 
## Multivariate Meta-Analysis Model (k = 710; method: REML)
## 
## Variance Components:
## 
##             estim    sqrt  nlvls  fixed                        factor 
## sigma^2.1  0.0802  0.2832     85     no                  Study_number 
## sigma^2.2  0.1186  0.3444    396     no  Study_number/Response_number 
## 
## Test for Residual Heterogeneity:
## QE(df = 703) = 19754.3111, p-val < .0001
## 
## Number of estimates:   710
## Number of clusters:    85
## Estimates per cluster: 1-48 (mean: 8.35, median: 6)
## 
## Test of Moderators (coefficients 1:7):¹
## F(df1 = 7, df2 = 78) = 4.9996, p-val = 0.0001
## 
## Model Results:
## 
##                                      estimate      se¹     tval¹  df¹    pval¹ 
## LnSR:Trait_catDevelopment success     -0.1333  0.0992   -1.3436   78   0.1830  
## LnSR:Trait_catFeeding and nutrition   -0.1095  0.1410   -0.7769   78   0.4396  
## LnSR:Trait_catGrowth                  -0.0208  0.0334   -0.6236   78   0.5347  
## LnSR:Trait_catMetabolic rate           0.2806  0.1101    2.5494   78   0.0128  
## LnSR:Trait_catMovement                -0.0111  0.0580   -0.1915   78   0.8486  
## LnSR:Trait_catReproductive success    -0.0226  0.0128   -1.7639   78   0.0817  
## LnSR:Trait_catSurvival                -0.4274  0.1166   -3.6639   78   0.0005  
##                                        ci.lb¹    ci.ub¹      
## LnSR:Trait_catDevelopment success    -0.3308    0.0642       
## LnSR:Trait_catFeeding and nutrition  -0.3902    0.1712       
## LnSR:Trait_catGrowth                 -0.0873    0.0456       
## LnSR:Trait_catMetabolic rate          0.0615    0.4997     * 
## LnSR:Trait_catMovement               -0.1265    0.1043       
## LnSR:Trait_catReproductive success   -0.0481    0.0029     . 
## LnSR:Trait_catSurvival               -0.6596   -0.1952   *** 
## 
## ---
## Signif. codes:  0 '***' 0.001 '**' 0.01 '*' 0.05 '.' 0.1 ' ' 1
## 
## 1) results based on cluster-robust inference (var-cov estimator: CR1,
##    approx t/F-tests and confidence intervals, df: residual method)
```

```
Rem_Hill_traitcat <- subset(Traitcatdata, Study_name !="Hill and Lawrence 2006")
model_rem_Hill_traitcat <- rma.mv(yi = LnRR, V = Variance, mods = ~LnSR:Trait_cat - 1, test="t", random = list(~1 |Study_number/Response_number), method = "REML", data = Rem_Hill_traitcat)
Traitcat_model_robust15 <- robust(model_rem_Hill_traitcat, cluster=Rem_Hill_traitcat$Study_number)
Traitcat_model_robust15
```

```
## 
## Multivariate Meta-Analysis Model (k = 663; method: REML)
## 
## Variance Components:
## 
##             estim    sqrt  nlvls  fixed                        factor 
## sigma^2.1  0.0836  0.2891     83     no                  Study_number 
## sigma^2.2  0.1322  0.3636    349     no  Study_number/Response_number 
## 
## Test for Residual Heterogeneity:
## QE(df = 656) = 19102.7659, p-val < .0001
## 
## Number of estimates:   663
## Number of clusters:    83
## Estimates per cluster: 1-48 (mean: 7.99, median: 6)
## 
## Test of Moderators (coefficients 1:7):¹
## F(df1 = 7, df2 = 76) = 5.9617, p-val < .0001
## 
## Model Results:
## 
##                                      estimate      se¹     tval¹  df¹    pval¹ 
## LnSR:Trait_catDevelopment success     -0.1333  0.0996   -1.3385   76   0.1847  
## LnSR:Trait_catFeeding and nutrition   -0.1300  0.1559   -0.8340   76   0.4069  
## LnSR:Trait_catGrowth                  -0.0198  0.0339   -0.5822   76   0.5622  
## LnSR:Trait_catMetabolic rate           0.3340  0.0987    3.3856   76   0.0011  
## LnSR:Trait_catMovement                -0.0109  0.0581   -0.1873   76   0.8519  
## LnSR:Trait_catReproductive success    -0.0223  0.0129   -1.7315   76   0.0874  
## LnSR:Trait_catSurvival                -0.4290  0.1181   -3.6332   76   0.0005  
##                                        ci.lb¹    ci.ub¹      
## LnSR:Trait_catDevelopment success    -0.3316    0.0650       
## LnSR:Trait_catFeeding and nutrition  -0.4405    0.1804       
## LnSR:Trait_catGrowth                 -0.0874    0.0478       
## LnSR:Trait_catMetabolic rate          0.1375    0.5305    ** 
## LnSR:Trait_catMovement               -0.1267    0.1049       
## LnSR:Trait_catReproductive success   -0.0479    0.0033     . 
## LnSR:Trait_catSurvival               -0.6641   -0.1938   *** 
## 
## ---
## Signif. codes:  0 '***' 0.001 '**' 0.01 '*' 0.05 '.' 0.1 ' ' 1
## 
## 1) results based on cluster-robust inference (var-cov estimator: CR1,
##    approx t/F-tests and confidence intervals, df: residual method)
```

```
Rem_Detree_traitcat <- subset(Traitcatdata, Study_name !="Detree et al. 2020")
model_rem_Detree_traitcat <- rma.mv(yi = LnRR, V = Variance, mods = ~LnSR:Trait_cat - 1, test="t", random = list(~1 |Study_number/Response_number), method = "REML", data = Rem_Detree_traitcat)
Traitcat_model_robust16 <- robust(model_rem_Detree_traitcat, cluster=Rem_Detree_traitcat$Study_number)
Traitcat_model_robust16
```

```
## 
## Multivariate Meta-Analysis Model (k = 702; method: REML)
## 
## Variance Components:
## 
##             estim    sqrt  nlvls  fixed                        factor 
## sigma^2.1  0.0830  0.2882     83     no                  Study_number 
## sigma^2.2  0.1204  0.3470    388     no  Study_number/Response_number 
## 
## Test for Residual Heterogeneity:
## QE(df = 695) = 19729.4776, p-val < .0001
## 
## Number of estimates:   702
## Number of clusters:    83
## Estimates per cluster: 1-48 (mean: 8.46, median: 6)
## 
## Test of Moderators (coefficients 1:7):¹
## F(df1 = 7, df2 = 76) = 4.9873, p-val = 0.0001
## 
## Model Results:
## 
##                                      estimate      se¹     tval¹  df¹    pval¹ 
## LnSR:Trait_catDevelopment success     -0.1333  0.0994   -1.3412   76   0.1839  
## LnSR:Trait_catFeeding and nutrition   -0.1130  0.1441   -0.7845   76   0.4352  
## LnSR:Trait_catGrowth                  -0.0211  0.0335   -0.6295   76   0.5309  
## LnSR:Trait_catMetabolic rate           0.2808  0.1108    2.5338   76   0.0133  
## LnSR:Trait_catMovement                -0.0098  0.0584   -0.1674   76   0.8675  
## LnSR:Trait_catReproductive success    -0.0225  0.0128   -1.7548   76   0.0833  
## LnSR:Trait_catSurvival                -0.4276  0.1170   -3.6550   76   0.0005  
##                                        ci.lb¹    ci.ub¹      
## LnSR:Trait_catDevelopment success    -0.3312    0.0646       
## LnSR:Trait_catFeeding and nutrition  -0.4001    0.1740       
## LnSR:Trait_catGrowth                 -0.0879    0.0457       
## LnSR:Trait_catMetabolic rate          0.0601    0.5015     * 
## LnSR:Trait_catMovement               -0.1262    0.1066       
## LnSR:Trait_catReproductive success   -0.0481    0.0030     . 
## LnSR:Trait_catSurvival               -0.6607   -0.1946   *** 
## 
## ---
## Signif. codes:  0 '***' 0.001 '**' 0.01 '*' 0.05 '.' 0.1 ' ' 1
## 
## 1) results based on cluster-robust inference (var-cov estimator: CR1,
##    approx t/F-tests and confidence intervals, df: residual method)
```

```
Rem_Kuhnhold_traitcat <- subset(Traitcatdata, Study_name !="Kuhnhold et al. 2019")
model_rem_Kuhnhold_traitcat <- rma.mv(yi = LnRR, V = Variance, mods = ~LnSR:Trait_cat - 1, test="t", random = list(~1 |Study_number/Response_number), method = "REML", data = Rem_Kuhnhold_traitcat)
Traitcat_model_robust17 <- robust(model_rem_Kuhnhold_traitcat, cluster=Rem_Kuhnhold_traitcat$Study_number)
Traitcat_model_robust17
```

```
## 
## Multivariate Meta-Analysis Model (k = 694; method: REML)
## 
## Variance Components:
## 
##             estim    sqrt  nlvls  fixed                        factor 
## sigma^2.1  0.0810  0.2847     83     no                  Study_number 
## sigma^2.2  0.1143  0.3381    380     no  Study_number/Response_number 
## 
## Test for Residual Heterogeneity:
## QE(df = 687) = 19196.2555, p-val < .0001
## 
## Number of estimates:   694
## Number of clusters:    83
## Estimates per cluster: 1-48 (mean: 8.36, median: 6)
## 
## Test of Moderators (coefficients 1:7):¹
## F(df1 = 7, df2 = 76) = 4.9460, p-val = 0.0001
## 
## Model Results:
## 
##                                      estimate      se¹     tval¹  df¹    pval¹ 
## LnSR:Trait_catDevelopment success     -0.1333  0.0992   -1.3431   76   0.1832  
## LnSR:Trait_catFeeding and nutrition   -0.1140  0.1434   -0.7951   76   0.4290  
## LnSR:Trait_catGrowth                  -0.0209  0.0334   -0.6254   76   0.5336  
## LnSR:Trait_catMetabolic rate           0.2869  0.1105    2.5955   76   0.0113  
## LnSR:Trait_catMovement                -0.0112  0.0580   -0.1937   76   0.8469  
## LnSR:Trait_catReproductive success    -0.0227  0.0128   -1.7692   76   0.0809  
## LnSR:Trait_catSurvival                -0.4268  0.1164   -3.6683   76   0.0005  
##                                        ci.lb¹    ci.ub¹      
## LnSR:Trait_catDevelopment success    -0.3309    0.0644       
## LnSR:Trait_catFeeding and nutrition  -0.3995    0.1715       
## LnSR:Trait_catGrowth                 -0.0875    0.0457       
## LnSR:Trait_catMetabolic rate          0.0667    0.5071     * 
## LnSR:Trait_catMovement               -0.1268    0.1043       
## LnSR:Trait_catReproductive success   -0.0482    0.0029     . 
## LnSR:Trait_catSurvival               -0.6586   -0.1951   *** 
## 
## ---
## Signif. codes:  0 '***' 0.001 '**' 0.01 '*' 0.05 '.' 0.1 ' ' 1
## 
## 1) results based on cluster-robust inference (var-cov estimator: CR1,
##    approx t/F-tests and confidence intervals, df: residual method)
```

# Models with observations removed where the experimental temperature is lower than the future (+2.58C) mean annual temperature (MAT)

## Loading data

```
setwd('/Users/bethanlang/Documents/PhD/Literature Review')
Traitcatdata_CC <- read_csv("Lang et al. 2022_dataset_CC2906.csv")
```

```
## Rows: 529 Columns: 43
## ── Column specification ────────────────────────────────────────────────────────
## Delimiter: ","
## chr  (8): Study_name, Species, Class, Lifestage, Latitude_cat, Trait_cat, Tr...
## dbl (35): Study_number, Year, Effect_size_id, Latitude_cont, Response_number...
## 
## ℹ Use `spec()` to retrieve the full column specification for this data.
## ℹ Specify the column types or set `show_col_types = FALSE` to quiet this message.
```

## Turning random effects into factors

```
Traitcatdata_CC$Study_number = factor(Traitcatdata_CC$Study_number)
Traitcatdata_CC$Response_number = factor(Traitcatdata_CC$Response_number)
```

## Ordering data

```
Traitcatdata_CC <- Traitcatdata_CC[order(Traitcatdata_CC$Trait_cat),]
```

## Number in each trait

```
Numbertraits <- Traitcatdata_CC %>% group_by(Trait_cat) %>% summarise(n()) 
Numbertraits$n <- Numbertraits$`n()`
```

## The model

```
Traitcat_model_CC <- rma.mv(yi = LnRR, V = Variance, mods = ~LnSR:Trait_cat-1, test="t", random = list(~1|Study_number/Response_number), method = "REML", data = Traitcatdata_CC)
print(Traitcat_model_CC)
```

```
## 
## Multivariate Meta-Analysis Model (k = 529; method: REML)
## 
## Variance Components:
## 
##             estim    sqrt  nlvls  fixed                        factor 
## sigma^2.1  0.1367  0.3697     79     no                  Study_number 
## sigma^2.2  0.1568  0.3960    364     no  Study_number/Response_number 
## 
## Test for Residual Heterogeneity:
## QE(df = 522) = 15823.7356, p-val < .0001
## 
## Test of Moderators (coefficients 1:7):
## F(df1 = 7, df2 = 522) = 57.3689, p-val < .0001
## 
## Model Results:
## 
##                                      estimate      se      tval   df    pval 
## LnSR:Trait_catDevelopment success     -0.4338  0.0293  -14.8138  522  <.0001 
## LnSR:Trait_catFeeding and nutrition   -0.2315  0.0288   -8.0465  522  <.0001 
## LnSR:Trait_catGrowth                  -0.0798  0.0250   -3.1881  522  0.0015 
## LnSR:Trait_catMetabolic rate           0.1610  0.0453    3.5511  522  0.0004 
## LnSR:Trait_catMovement                -0.1507  0.0772   -1.9525  522  0.0514 
## LnSR:Trait_catReproductive success    -0.0227  0.0195   -1.1630  522  0.2454 
## LnSR:Trait_catSurvival                -0.3567  0.0367   -9.7194  522  <.0001 
##                                        ci.lb    ci.ub      
## LnSR:Trait_catDevelopment success    -0.4913  -0.3762  *** 
## LnSR:Trait_catFeeding and nutrition  -0.2880  -0.1750  *** 
## LnSR:Trait_catGrowth                 -0.1290  -0.0306   ** 
## LnSR:Trait_catMetabolic rate          0.0719   0.2501  *** 
## LnSR:Trait_catMovement               -0.3023   0.0009    . 
## LnSR:Trait_catReproductive success   -0.0611   0.0156      
## LnSR:Trait_catSurvival               -0.4288  -0.2846  *** 
## 
## ---
## Signif. codes:  0 '***' 0.001 '**' 0.01 '*' 0.05 '.' 0.1 ' ' 1
```

## Robust model

```
Traitcat_model_robust_CC <- robust(Traitcat_model_CC, cluster=Traitcatdata_CC$Study_number)
Traitcat_model_robust_CC
```

```
## 
## Multivariate Meta-Analysis Model (k = 529; method: REML)
## 
## Variance Components:
## 
##             estim    sqrt  nlvls  fixed                        factor 
## sigma^2.1  0.1367  0.3697     79     no                  Study_number 
## sigma^2.2  0.1568  0.3960    364     no  Study_number/Response_number 
## 
## Test for Residual Heterogeneity:
## QE(df = 522) = 15823.7356, p-val < .0001
## 
## Number of estimates:   529
## Number of clusters:    79
## Estimates per cluster: 1-36 (mean: 6.70, median: 4)
## 
## Test of Moderators (coefficients 1:7):¹
## F(df1 = 7, df2 = 72) = 4.7591, p-val = 0.0002
## 
## Model Results:
## 
##                                      estimate      se¹     tval¹  df¹    pval¹ 
## LnSR:Trait_catDevelopment success     -0.4338  0.3570   -1.2150   72   0.2283  
## LnSR:Trait_catFeeding and nutrition   -0.2315  0.1494   -1.5498   72   0.1256  
## LnSR:Trait_catGrowth                  -0.0798  0.0872   -0.9151   72   0.3632  
## LnSR:Trait_catMetabolic rate           0.1610  0.0810    1.9869   72   0.0507  
## LnSR:Trait_catMovement                -0.1507  0.2853   -0.5282   72   0.5990  
## LnSR:Trait_catReproductive success    -0.0227  0.0568   -0.3996   72   0.6906  
## LnSR:Trait_catSurvival                -0.3567  0.1001   -3.5641   72   0.0007  
##                                        ci.lb¹    ci.ub¹      
## LnSR:Trait_catDevelopment success    -1.1455    0.2779       
## LnSR:Trait_catFeeding and nutrition  -0.5293    0.0663       
## LnSR:Trait_catGrowth                 -0.2536    0.0940       
## LnSR:Trait_catMetabolic rate         -0.0005    0.3226     . 
## LnSR:Trait_catMovement               -0.7193    0.4180       
## LnSR:Trait_catReproductive success   -0.1360    0.0905       
## LnSR:Trait_catSurvival               -0.5562   -0.1572   *** 
## 
## ---
## Signif. codes:  0 '***' 0.001 '**' 0.01 '*' 0.05 '.' 0.1 ' ' 1
## 
## 1) results based on cluster-robust inference (var-cov estimator: CR1,
##    approx t/F-tests and confidence intervals, df: residual method)
```

metabolism no longer significant

# Life stage

## Life stage (full model)

## Loading data

```
setwd('/Users/bethanlang/Documents/PhD/Literature Review')
Lifestagedata <- read_csv("Lang et al. 2022_dataset_2906_nometabolism.csv")
```

```
## Rows: 658 Columns: 44
## ── Column specification ────────────────────────────────────────────────────────
## Delimiter: ","
## chr  (9): Study_name, Species, Class, Lifestage, Latitude_cat, Latitude_cat2...
## dbl (35): Study_number, Year, Effect_size_id, Latitude_cont, Response_number...
## 
## ℹ Use `spec()` to retrieve the full column specification for this data.
## ℹ Specify the column types or set `show_col_types = FALSE` to quiet this message.
```

## Turning random effects into factors

```
Lifestagedata$Study_number = factor(Lifestagedata$Study_number)
Lifestagedata$Response_number = factor(Lifestagedata$Response_number)
```

## Ordering data

```
Lifestagedata <- Lifestagedata[order(Lifestagedata$Lifestage),]
```

## Number in each lifestage

```
Numberlifestages <- Lifestagedata %>% group_by(Lifestage) %>% summarise(n()) 
Numberlifestages$n <- Numberlifestages$`n()`
```

## The model

```
Lifestage_model <- rma.mv(yi = LnRR, V = Variance, mods = ~LnSR:Lifestage-1, test="t", random = list(~1|Study_number/Response_number), method = "REML", data = Lifestagedata)
print(Lifestage_model)
```

```
## 
## Multivariate Meta-Analysis Model (k = 658; method: REML)
## 
## Variance Components:
## 
##             estim    sqrt  nlvls  fixed                        factor 
## sigma^2.1  0.1044  0.3231     80     no                  Study_number 
## sigma^2.2  0.0887  0.2978    361     no  Study_number/Response_number 
## 
## Test for Residual Heterogeneity:
## QE(df = 653) = 21840.3726, p-val < .0001
## 
## Test of Moderators (coefficients 1:5):
## F(df1 = 5, df2 = 653) = 56.6941, p-val < .0001
## 
## Model Results:
## 
##                                  estimate      se      tval   df    pval 
## LnSR:Lifestagea.Gametes           -0.0277  0.0073   -3.7923  653  0.0002 
## LnSR:Lifestageb.Embryonic stage   -0.1504  0.0131  -11.5260  653  <.0001 
## LnSR:Lifestagec.Larval stage      -0.2208  0.0201  -11.0119  653  <.0001 
## LnSR:Lifestaged.Juvenile stage    -0.0926  0.0213   -4.3562  653  <.0001 
## LnSR:Lifestagee.Adult stage       -0.0094  0.0118   -0.7977  653  0.4253 
##                                    ci.lb    ci.ub      
## LnSR:Lifestagea.Gametes          -0.0420  -0.0133  *** 
## LnSR:Lifestageb.Embryonic stage  -0.1761  -0.1248  *** 
## LnSR:Lifestagec.Larval stage     -0.2602  -0.1814  *** 
## LnSR:Lifestaged.Juvenile stage   -0.1344  -0.0509  *** 
## LnSR:Lifestagee.Adult stage      -0.0327   0.0138      
## 
## ---
## Signif. codes:  0 '***' 0.001 '**' 0.01 '*' 0.05 '.' 0.1 ' ' 1
```

## Robust model

```
Lifestage_model_robust <- robust(Lifestage_model, cluster=Lifestagedata$Study_number)
Lifestage_model_robust
```

```
## 
## Multivariate Meta-Analysis Model (k = 658; method: REML)
## 
## Variance Components:
## 
##             estim    sqrt  nlvls  fixed                        factor 
## sigma^2.1  0.1044  0.3231     80     no                  Study_number 
## sigma^2.2  0.0887  0.2978    361     no  Study_number/Response_number 
## 
## Test for Residual Heterogeneity:
## QE(df = 653) = 21840.3726, p-val < .0001
## 
## Number of estimates:   658
## Number of clusters:    80
## Estimates per cluster: 1-36 (mean: 8.22, median: 6)
## 
## Test of Moderators (coefficients 1:5):¹
## F(df1 = 5, df2 = 75) = 4.6458, p-val = 0.0010
## 
## Model Results:
## 
##                                  estimate      se¹     tval¹  df¹    pval¹ 
## LnSR:Lifestagea.Gametes           -0.0277  0.0131   -2.1055   75   0.0386  
## LnSR:Lifestageb.Embryonic stage   -0.1504  0.1064   -1.4142   75   0.1615  
## LnSR:Lifestagec.Larval stage      -0.2208  0.0628   -3.5171   75   0.0007  
## LnSR:Lifestaged.Juvenile stage    -0.0926  0.0561   -1.6523   75   0.1026  
## LnSR:Lifestagee.Adult stage       -0.0094  0.0514   -0.1837   75   0.8548  
##                                    ci.lb¹    ci.ub¹      
## LnSR:Lifestagea.Gametes          -0.0539   -0.0015     * 
## LnSR:Lifestageb.Embryonic stage  -0.3623    0.0615       
## LnSR:Lifestagec.Larval stage     -0.3459   -0.0957   *** 
## LnSR:Lifestaged.Juvenile stage   -0.2043    0.0190       
## LnSR:Lifestagee.Adult stage      -0.1119    0.0930       
## 
## ---
## Signif. codes:  0 '***' 0.001 '**' 0.01 '*' 0.05 '.' 0.1 ' ' 1
## 
## 1) results based on cluster-robust inference (var-cov estimator: CR1,
##    approx t/F-tests and confidence intervals, df: residual method)
```

## Checking normality

```
res <- resid(Lifestage_model)
plot(fitted(Lifestage_model), res)
abline(0,0)
```

```
qqnorm(res)
qqline(res)
```

```
plot(density(res))
```

## Overall Publication bias

```
all <- trimfill(rma(LnRR, Variance, data = subset(Lifestagedata)),"left")$k0 
all2 <- trimfill(rma(LnRR, Variance, data = subset(Lifestagedata)),"right")$k0 
all
```

```
## [1] 150
```

```
all2
```

```
## [1] 0
```

## Sensitivity analysis

### Influential data points

```
Lifestagedata2 <- Lifestagedata %>%
  arrange(LnRR)
```

```
datest_lifestage <- Lifestagedata2[-1,]
modeltest_lifestage <- rma.mv(yi = LnRR, V = Variance, mods = ~LnSR:Lifestage - 1, test="t", random = list(~1 |Study_number/Response_number), method = "REML", data = datest_lifestage)
Lifestage_model_robust2 <- robust(modeltest_lifestage, cluster=datest_lifestage$Study_number)
Lifestage_model_robust2
```

```
## 
## Multivariate Meta-Analysis Model (k = 657; method: REML)
## 
## Variance Components:
## 
##             estim    sqrt  nlvls  fixed                        factor 
## sigma^2.1  0.1048  0.3238     80     no                  Study_number 
## sigma^2.2  0.0887  0.2978    361     no  Study_number/Response_number 
## 
## Test for Residual Heterogeneity:
## QE(df = 652) = 21617.0923, p-val < .0001
## 
## Number of estimates:   657
## Number of clusters:    80
## Estimates per cluster: 1-36 (mean: 8.21, median: 6)
## 
## Test of Moderators (coefficients 1:5):¹
## F(df1 = 5, df2 = 75) = 4.6479, p-val = 0.0009
## 
## Model Results:
## 
##                                  estimate      se¹     tval¹  df¹    pval¹ 
## LnSR:Lifestagea.Gametes           -0.0277  0.0131   -2.1050   75   0.0386  
## LnSR:Lifestageb.Embryonic stage   -0.1457  0.1055   -1.3809   75   0.1714  
## LnSR:Lifestagec.Larval stage      -0.2207  0.0628   -3.5166   75   0.0007  
## LnSR:Lifestaged.Juvenile stage    -0.0927  0.0561   -1.6523   75   0.1026  
## LnSR:Lifestagee.Adult stage       -0.0095  0.0514   -0.1839   75   0.8546  
##                                    ci.lb¹    ci.ub¹      
## LnSR:Lifestagea.Gametes          -0.0538   -0.0015     * 
## LnSR:Lifestageb.Embryonic stage  -0.3559    0.0645       
## LnSR:Lifestagec.Larval stage     -0.3458   -0.0957   *** 
## LnSR:Lifestaged.Juvenile stage   -0.2044    0.0191       
## LnSR:Lifestagee.Adult stage      -0.1119    0.0930       
## 
## ---
## Signif. codes:  0 '***' 0.001 '**' 0.01 '*' 0.05 '.' 0.1 ' ' 1
## 
## 1) results based on cluster-robust inference (var-cov estimator: CR1,
##    approx t/F-tests and confidence intervals, df: residual method)
```

```
datest_lifestage2 <- datest_lifestage[-1,]
modeltest_lifestage2 <- rma.mv(yi = LnRR, V = Variance, mods = ~LnSR:Lifestage - 1, test="t", random = list(~1 |Study_number/Response_number), method = "REML", data = datest_lifestage2)
Lifestage_model_robust3 <- robust(modeltest_lifestage2, cluster=datest_lifestage2$Study_number)
Lifestage_model_robust3
```

```
## 
## Multivariate Meta-Analysis Model (k = 656; method: REML)
## 
## Variance Components:
## 
##             estim    sqrt  nlvls  fixed                        factor 
## sigma^2.1  0.1049  0.3239     80     no                  Study_number 
## sigma^2.2  0.0885  0.2975    361     no  Study_number/Response_number 
## 
## Test for Residual Heterogeneity:
## QE(df = 651) = 21581.4538, p-val < .0001
## 
## Number of estimates:   656
## Number of clusters:    80
## Estimates per cluster: 1-36 (mean: 8.20, median: 6)
## 
## Test of Moderators (coefficients 1:5):¹
## F(df1 = 5, df2 = 75) = 4.6153, p-val = 0.0010
## 
## Model Results:
## 
##                                  estimate      se¹     tval¹  df¹    pval¹ 
## LnSR:Lifestagea.Gametes           -0.0277  0.0131   -2.1060   75   0.0385  
## LnSR:Lifestageb.Embryonic stage   -0.1457  0.1055   -1.3811   75   0.1713  
## LnSR:Lifestagec.Larval stage      -0.2190  0.0622   -3.5210   75   0.0007  
## LnSR:Lifestaged.Juvenile stage    -0.0927  0.0561   -1.6523   75   0.1026  
## LnSR:Lifestagee.Adult stage       -0.0095  0.0514   -0.1840   75   0.8545  
##                                    ci.lb¹    ci.ub¹      
## LnSR:Lifestagea.Gametes          -0.0538   -0.0015     * 
## LnSR:Lifestageb.Embryonic stage  -0.3559    0.0645       
## LnSR:Lifestagec.Larval stage     -0.3430   -0.0951   *** 
## LnSR:Lifestaged.Juvenile stage   -0.2044    0.0191       
## LnSR:Lifestagee.Adult stage      -0.1119    0.0930       
## 
## ---
## Signif. codes:  0 '***' 0.001 '**' 0.01 '*' 0.05 '.' 0.1 ' ' 1
## 
## 1) results based on cluster-robust inference (var-cov estimator: CR1,
##    approx t/F-tests and confidence intervals, df: residual method)
```

```
datest_lifestage3 <- datest_lifestage2[-1,]
modeltest_lifestage3 <- rma.mv(yi = LnRR, V = Variance, mods = ~LnSR:Lifestage - 1, test="t", random = list(~1 |Study_number/Response_number), method = "REML", data = datest_lifestage3)
Lifestage_model_robust4 <- robust(modeltest_lifestage3, cluster=datest_lifestage3$Study_number)
Lifestage_model_robust4
```

```
## 
## Multivariate Meta-Analysis Model (k = 655; method: REML)
## 
## Variance Components:
## 
##             estim    sqrt  nlvls  fixed                        factor 
## sigma^2.1  0.1050  0.3240     80     no                  Study_number 
## sigma^2.2  0.0885  0.2975    361     no  Study_number/Response_number 
## 
## Test for Residual Heterogeneity:
## QE(df = 650) = 21562.4180, p-val < .0001
## 
## Number of estimates:   655
## Number of clusters:    80
## Estimates per cluster: 1-36 (mean: 8.19, median: 6)
## 
## Test of Moderators (coefficients 1:5):¹
## F(df1 = 5, df2 = 75) = 4.6156, p-val = 0.0010
## 
## Model Results:
## 
##                                  estimate      se¹     tval¹  df¹    pval¹ 
## LnSR:Lifestagea.Gametes           -0.0277  0.0131   -2.1060   75   0.0386  
## LnSR:Lifestageb.Embryonic stage   -0.1452  0.1054   -1.3772   75   0.1725  
## LnSR:Lifestagec.Larval stage      -0.2190  0.0622   -3.5210   75   0.0007  
## LnSR:Lifestaged.Juvenile stage    -0.0927  0.0561   -1.6523   75   0.1026  
## LnSR:Lifestagee.Adult stage       -0.0095  0.0514   -0.1840   75   0.8545  
##                                    ci.lb¹    ci.ub¹      
## LnSR:Lifestagea.Gametes          -0.0538   -0.0015     * 
## LnSR:Lifestageb.Embryonic stage  -0.3553    0.0648       
## LnSR:Lifestagec.Larval stage     -0.3430   -0.0951   *** 
## LnSR:Lifestaged.Juvenile stage   -0.2044    0.0191       
## LnSR:Lifestagee.Adult stage      -0.1119    0.0930       
## 
## ---
## Signif. codes:  0 '***' 0.001 '**' 0.01 '*' 0.05 '.' 0.1 ' ' 1
## 
## 1) results based on cluster-robust inference (var-cov estimator: CR1,
##    approx t/F-tests and confidence intervals, df: residual method)
```

```
datest_lifestage4 <- datest_lifestage3[-1,]
modeltest_lifestage4 <- rma.mv(yi = LnRR, V = Variance, mods = ~LnSR:Lifestage - 1, test="t", random = list(~1 |Study_number/Response_number), method = "REML", data = datest_lifestage4)
Lifestage_model_robust5 <- robust(modeltest_lifestage4, cluster=datest_lifestage4$Study_number)
Lifestage_model_robust5
```

```
## 
## Multivariate Meta-Analysis Model (k = 654; method: REML)
## 
## Variance Components:
## 
##             estim    sqrt  nlvls  fixed                        factor 
## sigma^2.1  0.1051  0.3242     80     no                  Study_number 
## sigma^2.2  0.0887  0.2979    361     no  Study_number/Response_number 
## 
## Test for Residual Heterogeneity:
## QE(df = 649) = 21552.6777, p-val < .0001
## 
## Number of estimates:   654
## Number of clusters:    80
## Estimates per cluster: 1-36 (mean: 8.18, median: 6)
## 
## Test of Moderators (coefficients 1:5):¹
## F(df1 = 5, df2 = 75) = 4.6154, p-val = 0.0010
## 
## Model Results:
## 
##                                  estimate      se¹     tval¹  df¹    pval¹ 
## LnSR:Lifestagea.Gametes           -0.0277  0.0131   -2.1060   75   0.0386  
## LnSR:Lifestageb.Embryonic stage   -0.1449  0.1055   -1.3742   75   0.1735  
## LnSR:Lifestagec.Larval stage      -0.2191  0.0622   -3.5206   75   0.0007  
## LnSR:Lifestaged.Juvenile stage    -0.0927  0.0561   -1.6523   75   0.1026  
## LnSR:Lifestagee.Adult stage       -0.0095  0.0514   -0.1841   75   0.8545  
##                                    ci.lb¹    ci.ub¹      
## LnSR:Lifestagea.Gametes          -0.0538   -0.0015     * 
## LnSR:Lifestageb.Embryonic stage  -0.3550    0.0652       
## LnSR:Lifestagec.Larval stage     -0.3430   -0.0951   *** 
## LnSR:Lifestaged.Juvenile stage   -0.2044    0.0191       
## LnSR:Lifestagee.Adult stage      -0.1119    0.0930       
## 
## ---
## Signif. codes:  0 '***' 0.001 '**' 0.01 '*' 0.05 '.' 0.1 ' ' 1
## 
## 1) results based on cluster-robust inference (var-cov estimator: CR1,
##    approx t/F-tests and confidence intervals, df: residual method)
```

```
datest_lifestage5 <- datest_lifestage4[-1,]
modeltest_lifestage5 <- rma.mv(yi = LnRR, V = Variance, mods = ~LnSR:Lifestage - 1, test="t", random = list(~1 |Study_number/Response_number), method = "REML", data = datest_lifestage5)
Lifestage_model_robust6 <- robust(modeltest_lifestage5, cluster=datest_lifestage5$Study_number)
Lifestage_model_robust6
```

```
## 
## Multivariate Meta-Analysis Model (k = 653; method: REML)
## 
## Variance Components:
## 
##             estim    sqrt  nlvls  fixed                        factor 
## sigma^2.1  0.1050  0.3240     80     no                  Study_number 
## sigma^2.2  0.0887  0.2979    361     no  Study_number/Response_number 
## 
## Test for Residual Heterogeneity:
## QE(df = 648) = 21534.6398, p-val < .0001
## 
## Number of estimates:   653
## Number of clusters:    80
## Estimates per cluster: 1-36 (mean: 8.16, median: 6)
## 
## Test of Moderators (coefficients 1:5):¹
## F(df1 = 5, df2 = 75) = 4.6295, p-val = 0.0010
## 
## Model Results:
## 
##                                  estimate      se¹     tval¹  df¹    pval¹ 
## LnSR:Lifestagea.Gametes           -0.0277  0.0131   -2.1060   75   0.0386  
## LnSR:Lifestageb.Embryonic stage   -0.1449  0.1055   -1.3742   75   0.1735  
## LnSR:Lifestagec.Larval stage      -0.2190  0.0622   -3.5207   75   0.0007  
## LnSR:Lifestaged.Juvenile stage    -0.0909  0.0547   -1.6618   75   0.1007  
## LnSR:Lifestagee.Adult stage       -0.0095  0.0514   -0.1840   75   0.8545  
##                                    ci.lb¹    ci.ub¹      
## LnSR:Lifestagea.Gametes          -0.0538   -0.0015     * 
## LnSR:Lifestageb.Embryonic stage  -0.3550    0.0652       
## LnSR:Lifestagec.Larval stage     -0.3430   -0.0951   *** 
## LnSR:Lifestaged.Juvenile stage   -0.1999    0.0181       
## LnSR:Lifestagee.Adult stage      -0.1119    0.0930       
## 
## ---
## Signif. codes:  0 '***' 0.001 '**' 0.01 '*' 0.05 '.' 0.1 ' ' 1
## 
## 1) results based on cluster-robust inference (var-cov estimator: CR1,
##    approx t/F-tests and confidence intervals, df: residual method)
```

```
datest_lifestage6 <- datest_lifestage5[-1,]
modeltest_lifestage6 <- rma.mv(yi = LnRR, V = Variance, mods = ~LnSR:Lifestage - 1, test="t", random = list(~1 |Study_number/Response_number), method = "REML", data = datest_lifestage6)
Lifestage_model_robust7 <- robust(modeltest_lifestage6, cluster=datest_lifestage6$Study_number)
Lifestage_model_robust7
```

```
## 
## Multivariate Meta-Analysis Model (k = 652; method: REML)
## 
## Variance Components:
## 
##             estim    sqrt  nlvls  fixed                        factor 
## sigma^2.1  0.0906  0.3010     80     no                  Study_number 
## sigma^2.2  0.0893  0.2989    360     no  Study_number/Response_number 
## 
## Test for Residual Heterogeneity:
## QE(df = 647) = 21475.6285, p-val < .0001
## 
## Number of estimates:   652
## Number of clusters:    80
## Estimates per cluster: 1-36 (mean: 8.15, median: 6)
## 
## Test of Moderators (coefficients 1:5):¹
## F(df1 = 5, df2 = 75) = 4.6479, p-val = 0.0009
## 
## Model Results:
## 
##                                  estimate      se¹     tval¹  df¹    pval¹ 
## LnSR:Lifestagea.Gametes           -0.0277  0.0131   -2.1068   75   0.0385  
## LnSR:Lifestageb.Embryonic stage   -0.1449  0.1053   -1.3752   75   0.1732  
## LnSR:Lifestagec.Larval stage      -0.2182  0.0618   -3.5281   75   0.0007  
## LnSR:Lifestaged.Juvenile stage    -0.0901  0.0542   -1.6612   75   0.1009  
## LnSR:Lifestagee.Adult stage       -0.0090  0.0513   -0.1752   75   0.8614  
##                                    ci.lb¹    ci.ub¹      
## LnSR:Lifestagea.Gametes          -0.0538   -0.0015     * 
## LnSR:Lifestageb.Embryonic stage  -0.3547    0.0650       
## LnSR:Lifestagec.Larval stage     -0.3414   -0.0950   *** 
## LnSR:Lifestaged.Juvenile stage   -0.1982    0.0180       
## LnSR:Lifestagee.Adult stage      -0.1112    0.0932       
## 
## ---
## Signif. codes:  0 '***' 0.001 '**' 0.01 '*' 0.05 '.' 0.1 ' ' 1
## 
## 1) results based on cluster-robust inference (var-cov estimator: CR1,
##    approx t/F-tests and confidence intervals, df: residual method)
```

```
datest_lifestage7 <- datest_lifestage6[-1,]
modeltest_lifestage7 <- rma.mv(yi = LnRR, V = Variance, mods = ~LnSR:Lifestage - 1, test="t", random = list(~1 |Study_number/Response_number), method = "REML", data = datest_lifestage7)
Lifestage_model_robust8 <- robust(modeltest_lifestage7, cluster=datest_lifestage7$Study_number)
Lifestage_model_robust8
```

```
## 
## Multivariate Meta-Analysis Model (k = 651; method: REML)
## 
## Variance Components:
## 
##             estim    sqrt  nlvls  fixed                        factor 
## sigma^2.1  0.0909  0.3015     80     no                  Study_number 
## sigma^2.2  0.0894  0.2990    360     no  Study_number/Response_number 
## 
## Test for Residual Heterogeneity:
## QE(df = 646) = 21340.4290, p-val < .0001
## 
## Number of estimates:   651
## Number of clusters:    80
## Estimates per cluster: 1-36 (mean: 8.14, median: 6)
## 
## Test of Moderators (coefficients 1:5):¹
## F(df1 = 5, df2 = 75) = 4.6506, p-val = 0.0009
## 
## Model Results:
## 
##                                  estimate      se¹     tval¹  df¹    pval¹ 
## LnSR:Lifestagea.Gametes           -0.0277  0.0131   -2.1061   75   0.0385  
## LnSR:Lifestageb.Embryonic stage   -0.1415  0.1062   -1.3325   75   0.1867  
## LnSR:Lifestagec.Larval stage      -0.2181  0.0618   -3.5276   75   0.0007  
## LnSR:Lifestaged.Juvenile stage    -0.0901  0.0543   -1.6612   75   0.1009  
## LnSR:Lifestagee.Adult stage       -0.0090  0.0513   -0.1754   75   0.8613  
##                                    ci.lb¹    ci.ub¹      
## LnSR:Lifestagea.Gametes          -0.0538   -0.0015     * 
## LnSR:Lifestageb.Embryonic stage  -0.3530    0.0700       
## LnSR:Lifestagec.Larval stage     -0.3413   -0.0950   *** 
## LnSR:Lifestaged.Juvenile stage   -0.1982    0.0180       
## LnSR:Lifestagee.Adult stage      -0.1112    0.0932       
## 
## ---
## Signif. codes:  0 '***' 0.001 '**' 0.01 '*' 0.05 '.' 0.1 ' ' 1
## 
## 1) results based on cluster-robust inference (var-cov estimator: CR1,
##    approx t/F-tests and confidence intervals, df: residual method)
```

```
datest_lifestage8 <- datest_lifestage7[-1,]
modeltest_lifestage8 <- rma.mv(yi = LnRR, V = Variance, mods = ~LnSR:Lifestage - 1, test="t", random = list(~1 |Study_number/Response_number), method = "REML", data = datest_lifestage8)
Lifestage_model_robust9 <- robust(modeltest_lifestage8, cluster=datest_lifestage8$Study_number)
Lifestage_model_robust9
```

```
## 
## Multivariate Meta-Analysis Model (k = 650; method: REML)
## 
## Variance Components:
## 
##             estim    sqrt  nlvls  fixed                        factor 
## sigma^2.1  0.0825  0.2873     80     no                  Study_number 
## sigma^2.2  0.0874  0.2957    359     no  Study_number/Response_number 
## 
## Test for Residual Heterogeneity:
## QE(df = 645) = 21291.7860, p-val < .0001
## 
## Number of estimates:   650
## Number of clusters:    80
## Estimates per cluster: 1-36 (mean: 8.12, median: 6)
## 
## Test of Moderators (coefficients 1:5):¹
## F(df1 = 5, df2 = 75) = 4.6660, p-val = 0.0009
## 
## Model Results:
## 
##                                  estimate      se¹     tval¹  df¹    pval¹ 
## LnSR:Lifestagea.Gametes           -0.0277  0.0131   -2.1063   75   0.0385  
## LnSR:Lifestageb.Embryonic stage   -0.1411  0.1060   -1.3312   75   0.1872  
## LnSR:Lifestagec.Larval stage      -0.2174  0.0615   -3.5362   75   0.0007  
## LnSR:Lifestaged.Juvenile stage    -0.0895  0.0539   -1.6602   75   0.1010  
## LnSR:Lifestagee.Adult stage       -0.0087  0.0512   -0.1688   75   0.8664  
##                                    ci.lb¹    ci.ub¹      
## LnSR:Lifestagea.Gametes          -0.0538   -0.0015     * 
## LnSR:Lifestageb.Embryonic stage  -0.3523    0.0701       
## LnSR:Lifestagec.Larval stage     -0.3398   -0.0949   *** 
## LnSR:Lifestaged.Juvenile stage   -0.1968    0.0179       
## LnSR:Lifestagee.Adult stage      -0.1107    0.0934       
## 
## ---
## Signif. codes:  0 '***' 0.001 '**' 0.01 '*' 0.05 '.' 0.1 ' ' 1
## 
## 1) results based on cluster-robust inference (var-cov estimator: CR1,
##    approx t/F-tests and confidence intervals, df: residual method)
```

```
datest_lifestage9 <- datest_lifestage8[-1,]
modeltest_lifestage9 <- rma.mv(yi = LnRR, V = Variance, mods = ~LnSR:Lifestage - 1, test="t", random = list(~1 |Study_number/Response_number), method = "REML", data = datest_lifestage9)
Lifestage_model_robust10 <- robust(modeltest_lifestage9, cluster=datest_lifestage9$Study_number)
Lifestage_model_robust10
```

```
## 
## Multivariate Meta-Analysis Model (k = 649; method: REML)
## 
## Variance Components:
## 
##             estim    sqrt  nlvls  fixed                        factor 
## sigma^2.1  0.0818  0.2859     80     no                  Study_number 
## sigma^2.2  0.0872  0.2953    359     no  Study_number/Response_number 
## 
## Test for Residual Heterogeneity:
## QE(df = 644) = 21262.7050, p-val < .0001
## 
## Number of estimates:   649
## Number of clusters:    80
## Estimates per cluster: 1-36 (mean: 8.11, median: 6)
## 
## Test of Moderators (coefficients 1:5):¹
## F(df1 = 5, df2 = 75) = 4.6722, p-val = 0.0009
## 
## Model Results:
## 
##                                  estimate      se¹     tval¹  df¹    pval¹ 
## LnSR:Lifestagea.Gametes           -0.0277  0.0131   -2.1086   75   0.0383  
## LnSR:Lifestageb.Embryonic stage   -0.1406  0.1059   -1.3275   75   0.1884  
## LnSR:Lifestagec.Larval stage      -0.2173  0.0614   -3.5371   75   0.0007  
## LnSR:Lifestaged.Juvenile stage    -0.0894  0.0539   -1.6601   75   0.1011  
## LnSR:Lifestagee.Adult stage       -0.0086  0.0512   -0.1682   75   0.8669  
##                                    ci.lb¹    ci.ub¹      
## LnSR:Lifestagea.Gametes          -0.0539   -0.0015     * 
## LnSR:Lifestageb.Embryonic stage  -0.3516    0.0704       
## LnSR:Lifestagec.Larval stage     -0.3396   -0.0949   *** 
## LnSR:Lifestaged.Juvenile stage   -0.1967    0.0179       
## LnSR:Lifestagee.Adult stage      -0.1107    0.0934       
## 
## ---
## Signif. codes:  0 '***' 0.001 '**' 0.01 '*' 0.05 '.' 0.1 ' ' 1
## 
## 1) results based on cluster-robust inference (var-cov estimator: CR1,
##    approx t/F-tests and confidence intervals, df: residual method)
```

```
datest_lifestage10 <- datest_lifestage9[-649,]
modeltest_lifestage10 <- rma.mv(yi = LnRR, V = Variance, mods = ~LnSR:Lifestage - 1, test="t", random = list(~1 |Study_number/Response_number), method = "REML", data = datest_lifestage10)
Lifestage_model_robust11 <- robust(modeltest_lifestage10, cluster=datest_lifestage10$Study_number)
Lifestage_model_robust11
```

```
## 
## Multivariate Meta-Analysis Model (k = 648; method: REML)
## 
## Variance Components:
## 
##             estim    sqrt  nlvls  fixed                        factor 
## sigma^2.1  0.0798  0.2826     80     no                  Study_number 
## sigma^2.2  0.0872  0.2954    359     no  Study_number/Response_number 
## 
## Test for Residual Heterogeneity:
## QE(df = 643) = 21255.6988, p-val < .0001
## 
## Number of estimates:   648
## Number of clusters:    80
## Estimates per cluster: 1-36 (mean: 8.10, median: 6)
## 
## Test of Moderators (coefficients 1:5):¹
## F(df1 = 5, df2 = 75) = 4.6763, p-val = 0.0009
## 
## Model Results:
## 
##                                  estimate      se¹     tval¹  df¹    pval¹ 
## LnSR:Lifestagea.Gametes           -0.0277  0.0131   -2.1087   75   0.0383  
## LnSR:Lifestageb.Embryonic stage   -0.1406  0.1059   -1.3282   75   0.1881  
## LnSR:Lifestagec.Larval stage      -0.2171  0.0614   -3.5384   75   0.0007  
## LnSR:Lifestaged.Juvenile stage    -0.0895  0.0539   -1.6611   75   0.1009  
## LnSR:Lifestagee.Adult stage       -0.0085  0.0512   -0.1666   75   0.8682  
##                                    ci.lb¹    ci.ub¹      
## LnSR:Lifestagea.Gametes          -0.0539   -0.0015     * 
## LnSR:Lifestageb.Embryonic stage  -0.3516    0.0703       
## LnSR:Lifestagec.Larval stage     -0.3394   -0.0949   *** 
## LnSR:Lifestaged.Juvenile stage   -0.1969    0.0178       
## LnSR:Lifestagee.Adult stage      -0.1105    0.0935       
## 
## ---
## Signif. codes:  0 '***' 0.001 '**' 0.01 '*' 0.05 '.' 0.1 ' ' 1
## 
## 1) results based on cluster-robust inference (var-cov estimator: CR1,
##    approx t/F-tests and confidence intervals, df: residual method)
```

### Removing papers that contribute with 2 or more studies.

```
More1study_lifestage <- Lifestagedata %>% group_by(Study_name) %>% summarise(unique(Study_number)) %>% summarise(n())
```

```
## Warning: Returning more (or less) than 1 row per `summarise()` group was deprecated in
## dplyr 1.1.0.
## ℹ Please use `reframe()` instead.
## ℹ When switching from `summarise()` to `reframe()`, remember that `reframe()`
##   always returns an ungrouped data frame and adjust accordingly.
## Call `lifecycle::last_lifecycle_warnings()` to see where this warning was
## generated.
```

```
## `summarise()` has grouped output by 'Study_name'. You can override using the
## `.groups` argument.
```

```
More1study_lifestage$n <- More1study_lifestage$`n()`
subset(More1study_lifestage, n >1)
```

```
## # A tibble: 6 × 3
##   Study_name                     `n()`     n
##   <chr>                          <int> <int>
## 1 Benitez Villalobos et al. 2006     3     3
## 2 Detree et al. 2020                 2     2
## 3 Garcia et al. 2018                 4     4
## 4 Hill and Lawrence 2006             2     2
## 5 Kuhnhold et al. 2019               2     2
## 6 Rupp 1973                          6     6
```

```
Rem_Rupp_lifestage <- subset(Lifestagedata, Study_name !="Rupp 1973")
model_rem_Rupp_lifestage <- rma.mv(yi = LnRR, V = Variance, mods = ~LnSR:Lifestage - 1, test="t", random = list(~1 |Study_number/Response_number), method = "REML", data = Rem_Rupp_lifestage)
Lifestage_model_robust12 <- robust(model_rem_Rupp_lifestage, cluster=Rem_Rupp_lifestage$Study_number)
Lifestage_model_robust12
```

```
## 
## Multivariate Meta-Analysis Model (k = 630; method: REML)
## 
## Variance Components:
## 
##             estim    sqrt  nlvls  fixed                        factor 
## sigma^2.1  0.1094  0.3308     74     no                  Study_number 
## sigma^2.2  0.0897  0.2995    351     no  Study_number/Response_number 
## 
## Test for Residual Heterogeneity:
## QE(df = 625) = 20910.8235, p-val < .0001
## 
## Number of estimates:   630
## Number of clusters:    74
## Estimates per cluster: 1-36 (mean: 8.51, median: 6)
## 
## Test of Moderators (coefficients 1:5):¹
## F(df1 = 5, df2 = 69) = 3.2122, p-val = 0.0115
## 
## Model Results:
## 
##                                  estimate      se¹     tval¹  df¹    pval¹ 
## LnSR:Lifestagea.Gametes           -0.0127  0.0288   -0.4407   69   0.6608  
## LnSR:Lifestageb.Embryonic stage   -0.1981  0.1499   -1.3218   69   0.1906  
## LnSR:Lifestagec.Larval stage      -0.2220  0.0633   -3.5059   69   0.0008  
## LnSR:Lifestaged.Juvenile stage    -0.0929  0.0564   -1.6480   69   0.1039  
## LnSR:Lifestagee.Adult stage       -0.0096  0.0516   -0.1857   69   0.8532  
##                                    ci.lb¹    ci.ub¹      
## LnSR:Lifestagea.Gametes          -0.0700    0.0447       
## LnSR:Lifestageb.Embryonic stage  -0.4970    0.1009       
## LnSR:Lifestagec.Larval stage     -0.3483   -0.0957   *** 
## LnSR:Lifestaged.Juvenile stage   -0.2054    0.0196       
## LnSR:Lifestagee.Adult stage      -0.1126    0.0934       
## 
## ---
## Signif. codes:  0 '***' 0.001 '**' 0.01 '*' 0.05 '.' 0.1 ' ' 1
## 
## 1) results based on cluster-robust inference (var-cov estimator: CR1,
##    approx t/F-tests and confidence intervals, df: residual method)
```

```
Rem_Garcia_lifestage <- subset(Lifestagedata, Study_name !="Garcia et al. 2018")
model_rem_Garcia_lifestage <- rma.mv(yi = LnRR, V = Variance, mods = ~LnSR:Lifestage - 1, test="t", random = list(~1 |Study_number/Response_number), method = "REML", data = Rem_Garcia_lifestage)
Lifestage_model_robust13 <- robust(model_rem_Garcia_lifestage, cluster=Rem_Garcia_lifestage$Study_number)
Lifestage_model_robust13
```

```
## 
## Multivariate Meta-Analysis Model (k = 637; method: REML)
## 
## Variance Components:
## 
##             estim    sqrt  nlvls  fixed                        factor 
## sigma^2.1  0.1087  0.3297     76     no                  Study_number 
## sigma^2.2  0.0890  0.2983    350     no  Study_number/Response_number 
## 
## Test for Residual Heterogeneity:
## QE(df = 632) = 21323.2408, p-val < .0001
## 
## Number of estimates:   637
## Number of clusters:    76
## Estimates per cluster: 1-36 (mean: 8.38, median: 6)
## 
## Test of Moderators (coefficients 1:5):¹
## F(df1 = 5, df2 = 71) = 8.2469, p-val < .0001
## 
## Model Results:
## 
##                                  estimate      se¹     tval¹  df¹    pval¹ 
## LnSR:Lifestagea.Gametes           -0.0298  0.0147   -2.0310   71   0.0460  
## LnSR:Lifestageb.Embryonic stage   -0.1499  0.1065   -1.4072   71   0.1637  
## LnSR:Lifestagec.Larval stage      -0.1772  0.0299   -5.9313   71   <.0001  
## LnSR:Lifestaged.Juvenile stage    -0.0927  0.0562   -1.6493   71   0.1035  
## LnSR:Lifestagee.Adult stage       -0.0096  0.0516   -0.1857   71   0.8532  
##                                    ci.lb¹    ci.ub¹      
## LnSR:Lifestagea.Gametes          -0.0591   -0.0005     * 
## LnSR:Lifestageb.Embryonic stage  -0.3623    0.0625       
## LnSR:Lifestagec.Larval stage     -0.2367   -0.1176   *** 
## LnSR:Lifestaged.Juvenile stage   -0.2048    0.0194       
## LnSR:Lifestagee.Adult stage      -0.1124    0.0932       
## 
## ---
## Signif. codes:  0 '***' 0.001 '**' 0.01 '*' 0.05 '.' 0.1 ' ' 1
## 
## 1) results based on cluster-robust inference (var-cov estimator: CR1,
##    approx t/F-tests and confidence intervals, df: residual method)
```

```
Rem_Villalobos_lifestage <- subset(Lifestagedata, Study_name !="Villalobos et al. 2006")
model_rem_Villalobos_lifestage <- rma.mv(yi = LnRR, V = Variance, mods = ~LnSR:Lifestage - 1, test="t", random = list(~1 |Study_number/Response_number), method = "REML", data = Rem_Villalobos_lifestage)
Lifestage_model_robust14 <- robust(model_rem_Villalobos_lifestage, cluster=Rem_Villalobos_lifestage$Study_number)
Lifestage_model_robust14
```

```
## 
## Multivariate Meta-Analysis Model (k = 658; method: REML)
## 
## Variance Components:
## 
##             estim    sqrt  nlvls  fixed                        factor 
## sigma^2.1  0.1044  0.3231     80     no                  Study_number 
## sigma^2.2  0.0887  0.2978    361     no  Study_number/Response_number 
## 
## Test for Residual Heterogeneity:
## QE(df = 653) = 21840.3726, p-val < .0001
## 
## Number of estimates:   658
## Number of clusters:    80
## Estimates per cluster: 1-36 (mean: 8.22, median: 6)
## 
## Test of Moderators (coefficients 1:5):¹
## F(df1 = 5, df2 = 75) = 4.6458, p-val = 0.0010
## 
## Model Results:
## 
##                                  estimate      se¹     tval¹  df¹    pval¹ 
## LnSR:Lifestagea.Gametes           -0.0277  0.0131   -2.1055   75   0.0386  
## LnSR:Lifestageb.Embryonic stage   -0.1504  0.1064   -1.4142   75   0.1615  
## LnSR:Lifestagec.Larval stage      -0.2208  0.0628   -3.5171   75   0.0007  
## LnSR:Lifestaged.Juvenile stage    -0.0926  0.0561   -1.6523   75   0.1026  
## LnSR:Lifestagee.Adult stage       -0.0094  0.0514   -0.1837   75   0.8548  
##                                    ci.lb¹    ci.ub¹      
## LnSR:Lifestagea.Gametes          -0.0539   -0.0015     * 
## LnSR:Lifestageb.Embryonic stage  -0.3623    0.0615       
## LnSR:Lifestagec.Larval stage     -0.3459   -0.0957   *** 
## LnSR:Lifestaged.Juvenile stage   -0.2043    0.0190       
## LnSR:Lifestagee.Adult stage      -0.1119    0.0930       
## 
## ---
## Signif. codes:  0 '***' 0.001 '**' 0.01 '*' 0.05 '.' 0.1 ' ' 1
## 
## 1) results based on cluster-robust inference (var-cov estimator: CR1,
##    approx t/F-tests and confidence intervals, df: residual method)
```

```
Rem_Hill_lifestage <- subset(Lifestagedata, Study_name !="Hill and Lawrence 2006")
model_rem_Hill_lifestage <- rma.mv(yi = LnRR, V = Variance, mods = ~LnSR:Lifestage - 1, test="t", random = list(~1 |Study_number/Response_number), method = "REML", data = Rem_Hill_lifestage)
Lifestage_model_robust15 <- robust(model_rem_Hill_lifestage, cluster=Rem_Hill_lifestage$Study_number)
Lifestage_model_robust15
```

```
## 
## Multivariate Meta-Analysis Model (k = 619; method: REML)
## 
## Variance Components:
## 
##             estim    sqrt  nlvls  fixed                        factor 
## sigma^2.1  0.1067  0.3266     78     no                  Study_number 
## sigma^2.2  0.1061  0.3258    322     no  Study_number/Response_number 
## 
## Test for Residual Heterogeneity:
## QE(df = 614) = 21435.8509, p-val < .0001
## 
## Number of estimates:   619
## Number of clusters:    78
## Estimates per cluster: 1-36 (mean: 7.94, median: 6)
## 
## Test of Moderators (coefficients 1:5):¹
## F(df1 = 5, df2 = 73) = 4.6197, p-val = 0.0010
## 
## Model Results:
## 
##                                  estimate      se¹     tval¹  df¹    pval¹ 
## LnSR:Lifestagea.Gametes           -0.0277  0.0131   -2.1059   73   0.0387  
## LnSR:Lifestageb.Embryonic stage   -0.1500  0.1067   -1.4053   73   0.1642  
## LnSR:Lifestagec.Larval stage      -0.2219  0.0636   -3.4907   73   0.0008  
## LnSR:Lifestaged.Juvenile stage    -0.0932  0.0564   -1.6519   73   0.1029  
## LnSR:Lifestagee.Adult stage       -0.0095  0.0519   -0.1840   73   0.8545  
##                                    ci.lb¹    ci.ub¹      
## LnSR:Lifestagea.Gametes          -0.0538   -0.0015     * 
## LnSR:Lifestageb.Embryonic stage  -0.3627    0.0627       
## LnSR:Lifestagec.Larval stage     -0.3485   -0.0952   *** 
## LnSR:Lifestaged.Juvenile stage   -0.2056    0.0192       
## LnSR:Lifestagee.Adult stage      -0.1129    0.0938       
## 
## ---
## Signif. codes:  0 '***' 0.001 '**' 0.01 '*' 0.05 '.' 0.1 ' ' 1
## 
## 1) results based on cluster-robust inference (var-cov estimator: CR1,
##    approx t/F-tests and confidence intervals, df: residual method)
```

```
Rem_Detree_lifestage <- subset(Lifestagedata, Study_name !="Detree et al. 2020")
model_rem_Detree_lifestage <- rma.mv(yi = LnRR, V = Variance, mods = ~LnSR:Lifestage - 1, test="t", random = list(~1 |Study_number/Response_number), method = "REML", data = Rem_Detree_lifestage)
Lifestage_model_robust16 <- robust(model_rem_Detree_lifestage, cluster=Rem_Detree_lifestage$Study_number)
Lifestage_model_robust16
```

```
## 
## Multivariate Meta-Analysis Model (k = 650; method: REML)
## 
## Variance Components:
## 
##             estim    sqrt  nlvls  fixed                        factor 
## sigma^2.1  0.1084  0.3292     78     no                  Study_number 
## sigma^2.2  0.0902  0.3004    353     no  Study_number/Response_number 
## 
## Test for Residual Heterogeneity:
## QE(df = 645) = 21815.7577, p-val < .0001
## 
## Number of estimates:   650
## Number of clusters:    78
## Estimates per cluster: 1-36 (mean: 8.33, median: 6)
## 
## Test of Moderators (coefficients 1:5):¹
## F(df1 = 5, df2 = 73) = 4.6317, p-val = 0.0010
## 
## Model Results:
## 
##                                  estimate      se¹     tval¹  df¹    pval¹ 
## LnSR:Lifestagea.Gametes           -0.0277  0.0132   -2.1037   73   0.0388  
## LnSR:Lifestageb.Embryonic stage   -0.1503  0.1065   -1.4110   73   0.1625  
## LnSR:Lifestagec.Larval stage      -0.2211  0.0630   -3.5097   73   0.0008  
## LnSR:Lifestaged.Juvenile stage    -0.0929  0.0563   -1.6510   73   0.1030  
## LnSR:Lifestagee.Adult stage       -0.0098  0.0517   -0.1888   73   0.8508  
##                                    ci.lb¹    ci.ub¹      
## LnSR:Lifestagea.Gametes          -0.0539   -0.0015     * 
## LnSR:Lifestageb.Embryonic stage  -0.3625    0.0620       
## LnSR:Lifestagec.Larval stage     -0.3467   -0.0956   *** 
## LnSR:Lifestaged.Juvenile stage   -0.2050    0.0192       
## LnSR:Lifestagee.Adult stage      -0.1129    0.0934       
## 
## ---
## Signif. codes:  0 '***' 0.001 '**' 0.01 '*' 0.05 '.' 0.1 ' ' 1
## 
## 1) results based on cluster-robust inference (var-cov estimator: CR1,
##    approx t/F-tests and confidence intervals, df: residual method)
```

```
Rem_Kuhnhold_lifestage <- subset(Lifestagedata, Study_name !="Kuhnhold et al. 2019")
model_rem_Kuhnhold_lifestage <- rma.mv(yi = LnRR, V = Variance, mods = ~LnSR:Lifestage - 1, test="t", random = list(~1 |Study_number/Response_number), method = "REML", data = Rem_Kuhnhold_lifestage)
Lifestage_model_robust17 <- robust(model_rem_Kuhnhold_lifestage, cluster=Rem_Kuhnhold_lifestage$Study_number)
Lifestage_model_robust17
```

```
## 
## Multivariate Meta-Analysis Model (k = 646; method: REML)
## 
## Variance Components:
## 
##             estim    sqrt  nlvls  fixed                        factor 
## sigma^2.1  0.1060  0.3255     78     no                  Study_number 
## sigma^2.2  0.0810  0.2846    349     no  Study_number/Response_number 
## 
## Test for Residual Heterogeneity:
## QE(df = 641) = 21314.4062, p-val < .0001
## 
## Number of estimates:   646
## Number of clusters:    78
## Estimates per cluster: 1-36 (mean: 8.28, median: 6)
## 
## Test of Moderators (coefficients 1:5):¹
## F(df1 = 5, df2 = 73) = 4.6378, p-val = 0.0010
## 
## Model Results:
## 
##                                  estimate      se¹     tval¹  df¹    pval¹ 
## LnSR:Lifestagea.Gametes           -0.0277  0.0132   -2.1023   73   0.0390  
## LnSR:Lifestageb.Embryonic stage   -0.1506  0.1063   -1.4162   73   0.1610  
## LnSR:Lifestagec.Larval stage      -0.2204  0.0625   -3.5251   73   0.0007  
## LnSR:Lifestaged.Juvenile stage    -0.0958  0.0585   -1.6364   73   0.1061  
## LnSR:Lifestagee.Adult stage       -0.0095  0.0515   -0.1839   73   0.8546  
##                                    ci.lb¹    ci.ub¹      
## LnSR:Lifestagea.Gametes          -0.0539   -0.0014     * 
## LnSR:Lifestageb.Embryonic stage  -0.3624    0.0613       
## LnSR:Lifestagec.Larval stage     -0.3450   -0.0958   *** 
## LnSR:Lifestaged.Juvenile stage   -0.2124    0.0209       
## LnSR:Lifestagee.Adult stage      -0.1120    0.0931       
## 
## ---
## Signif. codes:  0 '***' 0.001 '**' 0.01 '*' 0.05 '.' 0.1 ' ' 1
## 
## 1) results based on cluster-robust inference (var-cov estimator: CR1,
##    approx t/F-tests and confidence intervals, df: residual method)
```

# Models with observations removed where the experimental temperature is lower than the future (+2.58C) mean annual temperature (MAT)

## Loading data

```
setwd('/Users/bethanlang/Documents/PhD/Literature Review')
Lifestagedata_CC <- read_csv("Lang et al. 2022_dataset_CC2906_nometabolism.csv")
```

```
## Rows: 488 Columns: 44
## ── Column specification ────────────────────────────────────────────────────────
## Delimiter: ","
## chr  (9): Study_name, Species, Class, Lifestage, Latitude_cat, Latitude_cat2...
## dbl (35): Study_number, Year, Effect_size_id, Latitude_cont, Response_number...
## 
## ℹ Use `spec()` to retrieve the full column specification for this data.
## ℹ Specify the column types or set `show_col_types = FALSE` to quiet this message.
```

## Turning random effects into factors

```
Lifestagedata_CC$Study_number = factor(Lifestagedata_CC$Study_number)
Lifestagedata_CC$Response_number = factor(Lifestagedata_CC$Response_number)
```

## Ordering data

```
Lifestagedata_CC <- Lifestagedata_CC[order(Lifestagedata_CC$Lifestage),]
```

## Number in each lifestage

```
Numberlifestages <- Lifestagedata_CC %>% group_by(Lifestage) %>% summarise(n()) 
Numberlifestages$n <- Numberlifestages$`n()`
```

## The model

```
Lifestage_model_CC <- rma.mv(yi = LnRR, V = Variance, mods = ~LnSR:Lifestage-1, test="t", random = list(~1|Study_number/Response_number), method = "REML", data = Lifestagedata_CC)
print(Lifestage_model_CC)
```

```
## 
## Multivariate Meta-Analysis Model (k = 488; method: REML)
## 
## Variance Components:
## 
##             estim    sqrt  nlvls  fixed                        factor 
## sigma^2.1  0.2081  0.4562     74     no                  Study_number 
## sigma^2.2  0.1276  0.3571    332     no  Study_number/Response_number 
## 
## Test for Residual Heterogeneity:
## QE(df = 483) = 14857.3767, p-val < .0001
## 
## Test of Moderators (coefficients 1:5):
## F(df1 = 5, df2 = 483) = 90.0914, p-val < .0001
## 
## Model Results:
## 
##                                  estimate      se      tval   df    pval 
## LnSR:Lifestagea.Gametes           -0.0552  0.0250   -2.2122  483  0.0274 
## LnSR:Lifestageb.Embryonic stage   -0.4755  0.0294  -16.1956  483  <.0001 
## LnSR:Lifestagec.Larval stage      -0.1703  0.0278   -6.1170  483  <.0001 
## LnSR:Lifestaged.Juvenile stage    -0.5106  0.0409  -12.4695  483  <.0001 
## LnSR:Lifestagee.Adult stage       -0.0050  0.0228   -0.2203  483  0.8257 
##                                    ci.lb    ci.ub      
## LnSR:Lifestagea.Gametes          -0.1042  -0.0062    * 
## LnSR:Lifestageb.Embryonic stage  -0.5332  -0.4178  *** 
## LnSR:Lifestagec.Larval stage     -0.2250  -0.1156  *** 
## LnSR:Lifestaged.Juvenile stage   -0.5911  -0.4301  *** 
## LnSR:Lifestagee.Adult stage      -0.0497   0.0397      
## 
## ---
## Signif. codes:  0 '***' 0.001 '**' 0.01 '*' 0.05 '.' 0.1 ' ' 1
```

## Robust model

```
Lifestage_model_robust_CC <- robust(Lifestage_model_CC, cluster=Lifestagedata_CC$Study_number)
Lifestage_model_robust_CC
```

```
## 
## Multivariate Meta-Analysis Model (k = 488; method: REML)
## 
## Variance Components:
## 
##             estim    sqrt  nlvls  fixed                        factor 
## sigma^2.1  0.2081  0.4562     74     no                  Study_number 
## sigma^2.2  0.1276  0.3571    332     no  Study_number/Response_number 
## 
## Test for Residual Heterogeneity:
## QE(df = 483) = 14857.3767, p-val < .0001
## 
## Number of estimates:   488
## Number of clusters:    74
## Estimates per cluster: 1-30 (mean: 6.59, median: 4.5)
## 
## Test of Moderators (coefficients 1:5):¹
## F(df1 = 5, df2 = 69) = 15.7812, p-val < .0001
## 
## Model Results:
## 
##                                  estimate      se¹     tval¹  df¹    pval¹ 
## LnSR:Lifestagea.Gametes           -0.0552  0.0663   -0.8331   69   0.4076  
## LnSR:Lifestageb.Embryonic stage   -0.4755  0.3749   -1.2686   69   0.2089  
## LnSR:Lifestagec.Larval stage      -0.1703  0.0464   -3.6700   69   0.0005  
## LnSR:Lifestaged.Juvenile stage    -0.5106  0.0656   -7.7823   69   <.0001  
## LnSR:Lifestagee.Adult stage       -0.0050  0.0890   -0.0564   69   0.9552  
##                                    ci.lb¹    ci.ub¹      
## LnSR:Lifestagea.Gametes          -0.1874    0.0770       
## LnSR:Lifestageb.Embryonic stage  -1.2234    0.2723       
## LnSR:Lifestagec.Larval stage     -0.2629   -0.0777   *** 
## LnSR:Lifestaged.Juvenile stage   -0.6415   -0.3797   *** 
## LnSR:Lifestagee.Adult stage      -0.1825    0.1725       
## 
## ---
## Signif. codes:  0 '***' 0.001 '**' 0.01 '*' 0.05 '.' 0.1 ' ' 1
## 
## 1) results based on cluster-robust inference (var-cov estimator: CR1,
##    approx t/F-tests and confidence intervals, df: residual method)
```

# Class

## Class (full model)

```
setwd('/Users/bethanlang/Documents/PhD/Literature Review')
Classdata <- read_csv("Lang et al. 2022_dataset_2906_nometabolism.csv")
```

```
## Rows: 658 Columns: 44
## ── Column specification ────────────────────────────────────────────────────────
## Delimiter: ","
## chr  (9): Study_name, Species, Class, Lifestage, Latitude_cat, Latitude_cat2...
## dbl (35): Study_number, Year, Effect_size_id, Latitude_cont, Response_number...
## 
## ℹ Use `spec()` to retrieve the full column specification for this data.
## ℹ Specify the column types or set `show_col_types = FALSE` to quiet this message.
```

## Turning random effects into factors

```
Classdata$Study_number = factor(Classdata$Study_number)
Classdata$Response_number = factor(Classdata$Response_number)
```

## Ordering data

```
Classdata <- Classdata[order(Classdata$Class),]
```

## Number in each class

```
Numberclasses <- Classdata %>% group_by(Class) %>% summarise(n()) 
Numberclasses$n <- Numberclasses$`n()`
```

## The model

```
Class_model <- rma.mv(yi = LnRR, V = Variance, mods = ~LnSR:Class-1, test="t", random = list(~1|Study_number/Response_number), method = "REML", data = Classdata)
print(Class_model)
```

```
## 
## Multivariate Meta-Analysis Model (k = 658; method: REML)
## 
## Variance Components:
## 
##             estim    sqrt  nlvls  fixed                        factor 
## sigma^2.1  0.1144  0.3383     80     no                  Study_number 
## sigma^2.2  0.0896  0.2994    361     no  Study_number/Response_number 
## 
## Test for Residual Heterogeneity:
## QE(df = 654) = 24179.1460, p-val < .0001
## 
## Test of Moderators (coefficients 1:4):
## F(df1 = 4, df2 = 654) = 142.6969, p-val < .0001
## 
## Model Results:
## 
##                          estimate      se      tval   df    pval    ci.lb 
## LnSR:ClassAsteroidea      -0.3518  0.0157  -22.4800  654  <.0001  -0.3826 
## LnSR:ClassEchinoidea      -0.0066  0.0061   -1.0850  654  0.2783  -0.0186 
## LnSR:ClassHolothuroidea   -0.1100  0.0138   -7.9592  654  <.0001  -0.1371 
## LnSR:ClassOphiuroidea      0.1369  0.1436    0.9538  654  0.3406  -0.1450 
##                            ci.ub      
## LnSR:ClassAsteroidea     -0.3211  *** 
## LnSR:ClassEchinoidea      0.0054      
## LnSR:ClassHolothuroidea  -0.0829  *** 
## LnSR:ClassOphiuroidea     0.4188      
## 
## ---
## Signif. codes:  0 '***' 0.001 '**' 0.01 '*' 0.05 '.' 0.1 ' ' 1
```

## Robust model

```
Class_model_robust <- robust(Class_model, cluster=Classdata$Study_number)
Class_model_robust
```

```
## 
## Multivariate Meta-Analysis Model (k = 658; method: REML)
## 
## Variance Components:
## 
##             estim    sqrt  nlvls  fixed                        factor 
## sigma^2.1  0.1144  0.3383     80     no                  Study_number 
## sigma^2.2  0.0896  0.2994    361     no  Study_number/Response_number 
## 
## Test for Residual Heterogeneity:
## QE(df = 654) = 24179.1460, p-val < .0001
## 
## Number of estimates:   658
## Number of clusters:    80
## Estimates per cluster: 1-36 (mean: 8.22, median: 6)
## 
## Test of Moderators (coefficients 1:4):¹
## F(df1 = 4, df2 = 76) = 14.7350, p-val < .0001
## 
## Model Results:
## 
##                          estimate      se¹     tval¹  df¹    pval¹    ci.lb¹ 
## LnSR:ClassAsteroidea      -0.3518  0.0553   -6.3629   76   <.0001   -0.4619  
## LnSR:ClassEchinoidea      -0.0066  0.0141   -0.4721   76   0.6382   -0.0346  
## LnSR:ClassHolothuroidea   -0.1100  0.0268   -4.1047   76   0.0001   -0.1634  
## LnSR:ClassOphiuroidea      0.1369  0.1165    1.1755   76   0.2435   -0.0951  
##                            ci.ub¹      
## LnSR:ClassAsteroidea     -0.2417   *** 
## LnSR:ClassEchinoidea      0.0214       
## LnSR:ClassHolothuroidea  -0.0566   *** 
## LnSR:ClassOphiuroidea     0.3689       
## 
## ---
## Signif. codes:  0 '***' 0.001 '**' 0.01 '*' 0.05 '.' 0.1 ' ' 1
## 
## 1) results based on cluster-robust inference (var-cov estimator: CR1,
##    approx t/F-tests and confidence intervals, df: residual method)
```

## Checking assumptions

```
res <- resid(Class_model_robust)
plot(fitted(Class_model_robust), res)
abline(0,0)
```

```
qqnorm(res)
qqline(res)
```

```
plot(density(res))
```

## Sensitivity analysis

#### Influential data points

```
Classdata2 <- Classdata %>%
  arrange(LnRR)
```

```
datest_class <- Classdata2[-1,]
modeltest_class <- rma.mv(yi = LnRR, V = Variance, mods = ~LnSR:Class - 1, test="t", random = list(~1 |Study_number/Response_number), method = "REML", data = datest_class)
Class_model_robust2 <- robust(modeltest_class, cluster=datest_class$Study_number)
Class_model_robust2
```

```
## 
## Multivariate Meta-Analysis Model (k = 657; method: REML)
## 
## Variance Components:
## 
##             estim    sqrt  nlvls  fixed                        factor 
## sigma^2.1  0.1141  0.3378     80     no                  Study_number 
## sigma^2.2  0.0897  0.2995    361     no  Study_number/Response_number 
## 
## Test for Residual Heterogeneity:
## QE(df = 653) = 23961.1372, p-val < .0001
## 
## Number of estimates:   657
## Number of clusters:    80
## Estimates per cluster: 1-36 (mean: 8.21, median: 6)
## 
## Test of Moderators (coefficients 1:4):¹
## F(df1 = 4, df2 = 76) = 14.3562, p-val < .0001
## 
## Model Results:
## 
##                          estimate      se¹     tval¹  df¹    pval¹    ci.lb¹ 
## LnSR:ClassAsteroidea      -0.3452  0.0553   -6.2429   76   <.0001   -0.4554  
## LnSR:ClassEchinoidea      -0.0066  0.0141   -0.4720   76   0.6383   -0.0346  
## LnSR:ClassHolothuroidea   -0.1100  0.0268   -4.1044   76   0.0001   -0.1634  
## LnSR:ClassOphiuroidea      0.1369  0.1165    1.1756   76   0.2434   -0.0951  
##                            ci.ub¹      
## LnSR:ClassAsteroidea     -0.2351   *** 
## LnSR:ClassEchinoidea      0.0214       
## LnSR:ClassHolothuroidea  -0.0566   *** 
## LnSR:ClassOphiuroidea     0.3689       
## 
## ---
## Signif. codes:  0 '***' 0.001 '**' 0.01 '*' 0.05 '.' 0.1 ' ' 1
## 
## 1) results based on cluster-robust inference (var-cov estimator: CR1,
##    approx t/F-tests and confidence intervals, df: residual method)
```

```
datest_class2 <- datest_class[-1,]
modeltest_class2 <- rma.mv(yi = LnRR, V = Variance, mods = ~LnSR:Class - 1, test="t", random = list(~1 |Study_number/Response_number), method = "REML", data = datest_class2)
Class_model_robust3 <- robust(modeltest_class2, cluster=datest_class2$Study_number)
Class_model_robust3
```

```
## 
## Multivariate Meta-Analysis Model (k = 656; method: REML)
## 
## Variance Components:
## 
##             estim    sqrt  nlvls  fixed                        factor 
## sigma^2.1  0.1143  0.3381     80     no                  Study_number 
## sigma^2.2  0.0894  0.2990    361     no  Study_number/Response_number 
## 
## Test for Residual Heterogeneity:
## QE(df = 652) = 23925.7641, p-val < .0001
## 
## Number of estimates:   656
## Number of clusters:    80
## Estimates per cluster: 1-36 (mean: 8.20, median: 6)
## 
## Test of Moderators (coefficients 1:4):¹
## F(df1 = 4, df2 = 76) = 14.3537, p-val < .0001
## 
## Model Results:
## 
##                          estimate      se¹     tval¹  df¹    pval¹    ci.lb¹ 
## LnSR:ClassAsteroidea      -0.3452  0.0553   -6.2429   76   <.0001   -0.4554  
## LnSR:ClassEchinoidea      -0.0065  0.0141   -0.4596   76   0.6471   -0.0346  
## LnSR:ClassHolothuroidea   -0.1100  0.0268   -4.1045   76   0.0001   -0.1634  
## LnSR:ClassOphiuroidea      0.1370  0.1164    1.1762   76   0.2432   -0.0949  
##                            ci.ub¹      
## LnSR:ClassAsteroidea     -0.2351   *** 
## LnSR:ClassEchinoidea      0.0216       
## LnSR:ClassHolothuroidea  -0.0566   *** 
## LnSR:ClassOphiuroidea     0.3689       
## 
## ---
## Signif. codes:  0 '***' 0.001 '**' 0.01 '*' 0.05 '.' 0.1 ' ' 1
## 
## 1) results based on cluster-robust inference (var-cov estimator: CR1,
##    approx t/F-tests and confidence intervals, df: residual method)
```

```
datest_class3 <- datest_class2[-1,]
modeltest_class3 <- rma.mv(yi = LnRR, V = Variance, mods = ~LnSR:Class - 1, test="t", random = list(~1 |Study_number/Response_number), method = "REML", data = datest_class3)
modeltest_class3
```

```
## 
## Multivariate Meta-Analysis Model (k = 655; method: REML)
## 
## Variance Components:
## 
##             estim    sqrt  nlvls  fixed                        factor 
## sigma^2.1  0.1143  0.3381     80     no                  Study_number 
## sigma^2.2  0.0894  0.2990    361     no  Study_number/Response_number 
## 
## Test for Residual Heterogeneity:
## QE(df = 651) = 23905.4152, p-val < .0001
## 
## Test of Moderators (coefficients 1:4):
## F(df1 = 4, df2 = 651) = 137.8885, p-val < .0001
## 
## Model Results:
## 
##                          estimate      se      tval   df    pval    ci.lb 
## LnSR:ClassAsteroidea      -0.3452  0.0157  -22.0502  651  <.0001  -0.3760 
## LnSR:ClassEchinoidea      -0.0064  0.0061   -1.0428  651  0.2974  -0.0184 
## LnSR:ClassHolothuroidea   -0.1100  0.0138   -7.9590  651  <.0001  -0.1371 
## LnSR:ClassOphiuroidea      0.1370  0.1435    0.9545  651  0.3402  -0.1448 
##                            ci.ub      
## LnSR:ClassAsteroidea     -0.3145  *** 
## LnSR:ClassEchinoidea      0.0056      
## LnSR:ClassHolothuroidea  -0.0828  *** 
## LnSR:ClassOphiuroidea     0.4187      
## 
## ---
## Signif. codes:  0 '***' 0.001 '**' 0.01 '*' 0.05 '.' 0.1 ' ' 1
```

```
Class_model_robust4 <- robust(modeltest_class3, cluster=datest_class3$Study_number)
Class_model_robust4
```

```
## 
## Multivariate Meta-Analysis Model (k = 655; method: REML)
## 
## Variance Components:
## 
##             estim    sqrt  nlvls  fixed                        factor 
## sigma^2.1  0.1143  0.3381     80     no                  Study_number 
## sigma^2.2  0.0894  0.2990    361     no  Study_number/Response_number 
## 
## Test for Residual Heterogeneity:
## QE(df = 651) = 23905.4152, p-val < .0001
## 
## Number of estimates:   655
## Number of clusters:    80
## Estimates per cluster: 1-36 (mean: 8.19, median: 6)
## 
## Test of Moderators (coefficients 1:4):¹
## F(df1 = 4, df2 = 76) = 14.3517, p-val < .0001
## 
## Model Results:
## 
##                          estimate      se¹     tval¹  df¹    pval¹    ci.lb¹ 
## LnSR:ClassAsteroidea      -0.3452  0.0553   -6.2429   76   <.0001   -0.4554  
## LnSR:ClassEchinoidea      -0.0064  0.0142   -0.4505   76   0.6536   -0.0346  
## LnSR:ClassHolothuroidea   -0.1100  0.0268   -4.1045   76   0.0001   -0.1634  
## LnSR:ClassOphiuroidea      0.1370  0.1164    1.1762   76   0.2432   -0.0949  
##                            ci.ub¹      
## LnSR:ClassAsteroidea     -0.2351   *** 
## LnSR:ClassEchinoidea      0.0218       
## LnSR:ClassHolothuroidea  -0.0566   *** 
## LnSR:ClassOphiuroidea     0.3689       
## 
## ---
## Signif. codes:  0 '***' 0.001 '**' 0.01 '*' 0.05 '.' 0.1 ' ' 1
## 
## 1) results based on cluster-robust inference (var-cov estimator: CR1,
##    approx t/F-tests and confidence intervals, df: residual method)
```

```
datest_class4 <- datest_class3[-1,]
modeltest_class4 <- rma.mv(yi = LnRR, V = Variance, mods = ~LnSR:Class - 1, test="t", random = list(~1 |Study_number/Response_number), method = "REML", data = datest_class4)
Class_model_robust5 <- robust(modeltest_class4, cluster=datest_class4$Study_number)
Class_model_robust5
```

```
## 
## Multivariate Meta-Analysis Model (k = 654; method: REML)
## 
## Variance Components:
## 
##             estim    sqrt  nlvls  fixed                        factor 
## sigma^2.1  0.1143  0.3381     80     no                  Study_number 
## sigma^2.2  0.0896  0.2994    361     no  Study_number/Response_number 
## 
## Test for Residual Heterogeneity:
## QE(df = 650) = 23894.9269, p-val < .0001
## 
## Number of estimates:   654
## Number of clusters:    80
## Estimates per cluster: 1-36 (mean: 8.18, median: 6)
## 
## Test of Moderators (coefficients 1:4):¹
## F(df1 = 4, df2 = 76) = 14.3505, p-val < .0001
## 
## Model Results:
## 
##                          estimate      se¹     tval¹  df¹    pval¹    ci.lb¹ 
## LnSR:ClassAsteroidea      -0.3452  0.0553   -6.2430   76   <.0001   -0.4554  
## LnSR:ClassEchinoidea      -0.0063  0.0142   -0.4453   76   0.6574   -0.0345  
## LnSR:ClassHolothuroidea   -0.1100  0.0268   -4.1045   76   0.0001   -0.1634  
## LnSR:ClassOphiuroidea      0.1369  0.1165    1.1756   76   0.2434   -0.0951  
##                            ci.ub¹      
## LnSR:ClassAsteroidea     -0.2351   *** 
## LnSR:ClassEchinoidea      0.0219       
## LnSR:ClassHolothuroidea  -0.0566   *** 
## LnSR:ClassOphiuroidea     0.3689       
## 
## ---
## Signif. codes:  0 '***' 0.001 '**' 0.01 '*' 0.05 '.' 0.1 ' ' 1
## 
## 1) results based on cluster-robust inference (var-cov estimator: CR1,
##    approx t/F-tests and confidence intervals, df: residual method)
```

```
datest_class5 <- datest_class4[-1,]
modeltest_class5 <- rma.mv(yi = LnRR, V = Variance, mods = ~LnSR:Class - 1, test="t", random = list(~1 |Study_number/Response_number), method = "REML", data = datest_class5)
Class_model_robust6 <- robust(modeltest_class5, cluster=datest_class5$Study_number)
Class_model_robust6
```

```
## 
## Multivariate Meta-Analysis Model (k = 653; method: REML)
## 
## Variance Components:
## 
##             estim    sqrt  nlvls  fixed                        factor 
## sigma^2.1  0.1142  0.3379     80     no                  Study_number 
## sigma^2.2  0.0896  0.2994    361     no  Study_number/Response_number 
## 
## Test for Residual Heterogeneity:
## QE(df = 649) = 23878.4538, p-val < .0001
## 
## Number of estimates:   653
## Number of clusters:    80
## Estimates per cluster: 1-36 (mean: 8.16, median: 6)
## 
## Test of Moderators (coefficients 1:4):¹
## F(df1 = 4, df2 = 76) = 14.2940, p-val < .0001
## 
## Model Results:
## 
##                          estimate      se¹     tval¹  df¹    pval¹    ci.lb¹ 
## LnSR:ClassAsteroidea      -0.3444  0.0553   -6.2249   76   <.0001   -0.4545  
## LnSR:ClassEchinoidea      -0.0063  0.0142   -0.4453   76   0.6574   -0.0345  
## LnSR:ClassHolothuroidea   -0.1100  0.0268   -4.1044   76   0.0001   -0.1634  
## LnSR:ClassOphiuroidea      0.1369  0.1165    1.1757   76   0.2434   -0.0950  
##                            ci.ub¹      
## LnSR:ClassAsteroidea     -0.2342   *** 
## LnSR:ClassEchinoidea      0.0219       
## LnSR:ClassHolothuroidea  -0.0566   *** 
## LnSR:ClassOphiuroidea     0.3689       
## 
## ---
## Signif. codes:  0 '***' 0.001 '**' 0.01 '*' 0.05 '.' 0.1 ' ' 1
## 
## 1) results based on cluster-robust inference (var-cov estimator: CR1,
##    approx t/F-tests and confidence intervals, df: residual method)
```

```
datest_class6 <- datest_class5[-1,]
modeltest_class6 <- rma.mv(yi = LnRR, V = Variance, mods = ~LnSR:Class - 1, test="t", random = list(~1 |Study_number/Response_number), method = "REML", data = datest_class6)
Class_model_robust7 <- robust(modeltest_class6, cluster=datest_class6$Study_number)
Class_model_robust7
```

```
## 
## Multivariate Meta-Analysis Model (k = 652; method: REML)
## 
## Variance Components:
## 
##             estim    sqrt  nlvls  fixed                        factor 
## sigma^2.1  0.1023  0.3199     80     no                  Study_number 
## sigma^2.2  0.0898  0.2997    360     no  Study_number/Response_number 
## 
## Test for Residual Heterogeneity:
## QE(df = 648) = 23821.0923, p-val < .0001
## 
## Number of estimates:   652
## Number of clusters:    80
## Estimates per cluster: 1-36 (mean: 8.15, median: 6)
## 
## Test of Moderators (coefficients 1:4):¹
## F(df1 = 4, df2 = 76) = 14.2059, p-val < .0001
## 
## Model Results:
## 
##                          estimate      se¹     tval¹  df¹    pval¹    ci.lb¹ 
## LnSR:ClassAsteroidea      -0.3432  0.0553   -6.2029   76   <.0001   -0.4533  
## LnSR:ClassEchinoidea      -0.0063  0.0142   -0.4438   76   0.6584   -0.0345  
## LnSR:ClassHolothuroidea   -0.1097  0.0268   -4.0921   76   0.0001   -0.1631  
## LnSR:ClassOphiuroidea      0.1381  0.1165    1.1853   76   0.2396   -0.0939  
##                            ci.ub¹      
## LnSR:ClassAsteroidea     -0.2330   *** 
## LnSR:ClassEchinoidea      0.0219       
## LnSR:ClassHolothuroidea  -0.0563   *** 
## LnSR:ClassOphiuroidea     0.3700       
## 
## ---
## Signif. codes:  0 '***' 0.001 '**' 0.01 '*' 0.05 '.' 0.1 ' ' 1
## 
## 1) results based on cluster-robust inference (var-cov estimator: CR1,
##    approx t/F-tests and confidence intervals, df: residual method)
```

```
datest_class7 <- datest_class6[-1,]
modeltest_class7 <- rma.mv(yi = LnRR, V = Variance, mods = ~LnSR:Class - 1, test="t", random = list(~1 |Study_number/Response_number), method = "REML", data = datest_class7)
Class_model_robust8 <- robust(modeltest_class7, cluster=datest_class7$Study_number)
Class_model_robust8
```

```
## 
## Multivariate Meta-Analysis Model (k = 651; method: REML)
## 
## Variance Components:
## 
##             estim    sqrt  nlvls  fixed                        factor 
## sigma^2.1  0.1023  0.3199     80     no                  Study_number 
## sigma^2.2  0.0898  0.2997    360     no  Study_number/Response_number 
## 
## Test for Residual Heterogeneity:
## QE(df = 647) = 23673.6485, p-val < .0001
## 
## Number of estimates:   651
## Number of clusters:    80
## Estimates per cluster: 1-36 (mean: 8.14, median: 6)
## 
## Test of Moderators (coefficients 1:4):¹
## F(df1 = 4, df2 = 76) = 14.1961, p-val < .0001
## 
## Model Results:
## 
##                          estimate      se¹     tval¹  df¹    pval¹    ci.lb¹ 
## LnSR:ClassAsteroidea      -0.3431  0.0553   -6.2029   76   <.0001   -0.4533  
## LnSR:ClassEchinoidea      -0.0055  0.0139   -0.3979   76   0.6918   -0.0332  
## LnSR:ClassHolothuroidea   -0.1097  0.0268   -4.0921   76   0.0001   -0.1631  
## LnSR:ClassOphiuroidea      0.1381  0.1165    1.1853   76   0.2396   -0.0939  
##                            ci.ub¹      
## LnSR:ClassAsteroidea     -0.2330   *** 
## LnSR:ClassEchinoidea      0.0222       
## LnSR:ClassHolothuroidea  -0.0563   *** 
## LnSR:ClassOphiuroidea     0.3700       
## 
## ---
## Signif. codes:  0 '***' 0.001 '**' 0.01 '*' 0.05 '.' 0.1 ' ' 1
## 
## 1) results based on cluster-robust inference (var-cov estimator: CR1,
##    approx t/F-tests and confidence intervals, df: residual method)
```

```
datest_class8 <- datest_class7[-1,]
modeltest_class8 <- rma.mv(yi = LnRR, V = Variance, mods = ~LnSR:Class - 1, test="t", random = list(~1 |Study_number/Response_number), method = "REML", data = datest_class8)
Class_model_robust9 <- robust(modeltest_class8, cluster=datest_class8$Study_number)
Class_model_robust9
```

```
## 
## Multivariate Meta-Analysis Model (k = 650; method: REML)
## 
## Variance Components:
## 
##             estim    sqrt  nlvls  fixed                        factor 
## sigma^2.1  0.0965  0.3106     80     no                  Study_number 
## sigma^2.2  0.0876  0.2960    359     no  Study_number/Response_number 
## 
## Test for Residual Heterogeneity:
## QE(df = 646) = 23626.4541, p-val < .0001
## 
## Number of estimates:   650
## Number of clusters:    80
## Estimates per cluster: 1-36 (mean: 8.12, median: 6)
## 
## Test of Moderators (coefficients 1:4):¹
## F(df1 = 4, df2 = 76) = 14.0930, p-val < .0001
## 
## Model Results:
## 
##                          estimate      se¹     tval¹  df¹    pval¹    ci.lb¹ 
## LnSR:ClassAsteroidea      -0.3419  0.0554   -6.1727   76   <.0001   -0.4522  
## LnSR:ClassEchinoidea      -0.0055  0.0139   -0.3972   76   0.6924   -0.0332  
## LnSR:ClassHolothuroidea   -0.1095  0.0268   -4.0839   76   0.0001   -0.1629  
## LnSR:ClassOphiuroidea      0.1390  0.1161    1.1974   76   0.2349   -0.0922  
##                            ci.ub¹      
## LnSR:ClassAsteroidea     -0.2316   *** 
## LnSR:ClassEchinoidea      0.0222       
## LnSR:ClassHolothuroidea  -0.0561   *** 
## LnSR:ClassOphiuroidea     0.3702       
## 
## ---
## Signif. codes:  0 '***' 0.001 '**' 0.01 '*' 0.05 '.' 0.1 ' ' 1
## 
## 1) results based on cluster-robust inference (var-cov estimator: CR1,
##    approx t/F-tests and confidence intervals, df: residual method)
```

```
datest_class9 <- datest_class8[-1,]
modeltest_class9 <- rma.mv(yi = LnRR, V = Variance, mods = ~LnSR:Class - 1, test="t", random = list(~1 |Study_number/Response_number), method = "REML", data = datest_class9)
Class_model_robust10 <- robust(modeltest_class9, cluster=datest_class9$Study_number)
Class_model_robust10
```

```
## 
## Multivariate Meta-Analysis Model (k = 649; method: REML)
## 
## Variance Components:
## 
##             estim    sqrt  nlvls  fixed                        factor 
## sigma^2.1  0.0954  0.3089     80     no                  Study_number 
## sigma^2.2  0.0873  0.2955    359     no  Study_number/Response_number 
## 
## Test for Residual Heterogeneity:
## QE(df = 645) = 23595.0026, p-val < .0001
## 
## Number of estimates:   649
## Number of clusters:    80
## Estimates per cluster: 1-36 (mean: 8.11, median: 6)
## 
## Test of Moderators (coefficients 1:4):¹
## F(df1 = 4, df2 = 76) = 14.0857, p-val < .0001
## 
## Model Results:
## 
##                          estimate      se¹     tval¹  df¹    pval¹    ci.lb¹ 
## LnSR:ClassAsteroidea      -0.3418  0.0554   -6.1715   76   <.0001   -0.4521  
## LnSR:ClassEchinoidea      -0.0054  0.0139   -0.3898   76   0.6978   -0.0332  
## LnSR:ClassHolothuroidea   -0.1095  0.0268   -4.0823   76   0.0001   -0.1629  
## LnSR:ClassOphiuroidea      0.1392  0.1160    1.1994   76   0.2341   -0.0919  
##                            ci.ub¹      
## LnSR:ClassAsteroidea     -0.2315   *** 
## LnSR:ClassEchinoidea      0.0223       
## LnSR:ClassHolothuroidea  -0.0561   *** 
## LnSR:ClassOphiuroidea     0.3703       
## 
## ---
## Signif. codes:  0 '***' 0.001 '**' 0.01 '*' 0.05 '.' 0.1 ' ' 1
## 
## 1) results based on cluster-robust inference (var-cov estimator: CR1,
##    approx t/F-tests and confidence intervals, df: residual method)
```

```
datest_class10 <- datest_class9[-649,]
modeltest_class10 <- rma.mv(yi = LnRR, V = Variance, mods = ~LnSR:Class - 1, test="t", random = list(~1 |Study_number/Response_number), method = "REML", data = datest_class10)
Class_model_robust11 <- robust(modeltest_class10, cluster=datest_class10$Study_number)
Class_model_robust11
```

```
## 
## Multivariate Meta-Analysis Model (k = 648; method: REML)
## 
## Variance Components:
## 
##             estim    sqrt  nlvls  fixed                        factor 
## sigma^2.1  0.0936  0.3059     80     no                  Study_number 
## sigma^2.2  0.0873  0.2955    359     no  Study_number/Response_number 
## 
## Test for Residual Heterogeneity:
## QE(df = 644) = 23588.1036, p-val < .0001
## 
## Number of estimates:   648
## Number of clusters:    80
## Estimates per cluster: 1-36 (mean: 8.10, median: 6)
## 
## Test of Moderators (coefficients 1:4):¹
## F(df1 = 4, df2 = 76) = 14.0759, p-val < .0001
## 
## Model Results:
## 
##                          estimate      se¹     tval¹  df¹    pval¹    ci.lb¹ 
## LnSR:ClassAsteroidea      -0.3416  0.0554   -6.1694   76   <.0001   -0.4519  
## LnSR:ClassEchinoidea      -0.0054  0.0139   -0.3911   76   0.6968   -0.0332  
## LnSR:ClassHolothuroidea   -0.1094  0.0268   -4.0799   76   0.0001   -0.1628  
## LnSR:ClassOphiuroidea      0.1394  0.1160    1.2016   76   0.2332   -0.0917  
##                            ci.ub¹      
## LnSR:ClassAsteroidea     -0.2313   *** 
## LnSR:ClassEchinoidea      0.0223       
## LnSR:ClassHolothuroidea  -0.0560   *** 
## LnSR:ClassOphiuroidea     0.3704       
## 
## ---
## Signif. codes:  0 '***' 0.001 '**' 0.01 '*' 0.05 '.' 0.1 ' ' 1
## 
## 1) results based on cluster-robust inference (var-cov estimator: CR1,
##    approx t/F-tests and confidence intervals, df: residual method)
```

### Removing papers that contribute with 2 or more studies.

```
More1study_class <- Classdata %>% group_by(Study_name) %>% summarise(unique(Study_number)) %>% summarise(n())
```

```
## Warning: Returning more (or less) than 1 row per `summarise()` group was deprecated in
## dplyr 1.1.0.
## ℹ Please use `reframe()` instead.
## ℹ When switching from `summarise()` to `reframe()`, remember that `reframe()`
##   always returns an ungrouped data frame and adjust accordingly.
## Call `lifecycle::last_lifecycle_warnings()` to see where this warning was
## generated.
```

```
## `summarise()` has grouped output by 'Study_name'. You can override using the
## `.groups` argument.
```

```
More1study_class$n <- More1study_class$`n()`
subset(More1study_class, n >1)
```

```
## # A tibble: 6 × 3
##   Study_name                     `n()`     n
##   <chr>                          <int> <int>
## 1 Benitez Villalobos et al. 2006     3     3
## 2 Detree et al. 2020                 2     2
## 3 Garcia et al. 2018                 4     4
## 4 Hill and Lawrence 2006             2     2
## 5 Kuhnhold et al. 2019               2     2
## 6 Rupp 1973                          6     6
```

```
Rem_Rupp_class <- subset(Classdata, Study_name !="Rupp 1973")
model_rem_Rupp_class <- rma.mv(yi = LnRR, V = Variance, mods = ~LnSR:Class - 1, test="t", random = list(~1 |Study_number/Response_number), method = "REML", data = Rem_Rupp_class)
Class_model_robust12 <- robust(model_rem_Rupp_class, cluster=Rem_Rupp_class$Study_number)
Class_model_robust12
```

```
## 
## Multivariate Meta-Analysis Model (k = 630; method: REML)
## 
## Variance Components:
## 
##             estim    sqrt  nlvls  fixed                        factor 
## sigma^2.1  0.1210  0.3478     74     no                  Study_number 
## sigma^2.2  0.0920  0.3033    351     no  Study_number/Response_number 
## 
## Test for Residual Heterogeneity:
## QE(df = 626) = 21286.4303, p-val < .0001
## 
## Number of estimates:   630
## Number of clusters:    74
## Estimates per cluster: 1-36 (mean: 8.51, median: 6)
## 
## Test of Moderators (coefficients 1:4):¹
## F(df1 = 4, df2 = 70) = 13.2173, p-val < .0001
## 
## Model Results:
## 
##                          estimate      se¹     tval¹  df¹    pval¹    ci.lb¹ 
## LnSR:ClassAsteroidea      -0.3147  0.0537   -5.8617   70   <.0001   -0.4218  
## LnSR:ClassEchinoidea       0.0124  0.0218    0.5706   70   0.5701   -0.0310  
## LnSR:ClassHolothuroidea   -0.1101  0.0268   -4.1026   70   0.0001   -0.1637  
## LnSR:ClassOphiuroidea      0.1361  0.1171    1.1627   70   0.2489   -0.0974  
##                            ci.ub¹      
## LnSR:ClassAsteroidea     -0.2076   *** 
## LnSR:ClassEchinoidea      0.0559       
## LnSR:ClassHolothuroidea  -0.0566   *** 
## LnSR:ClassOphiuroidea     0.3696       
## 
## ---
## Signif. codes:  0 '***' 0.001 '**' 0.01 '*' 0.05 '.' 0.1 ' ' 1
## 
## 1) results based on cluster-robust inference (var-cov estimator: CR1,
##    approx t/F-tests and confidence intervals, df: residual method)
```

```
Rem_Garcia_class <- subset(Classdata, Study_name !="Garcia et al. 2018")
model_rem_Garcia_class <- rma.mv(yi = LnRR, V = Variance, mods = ~LnSR:Class - 1, test="t", random = list(~1 |Study_number/Response_number), method = "REML", data = Rem_Garcia_class)
Class_model_robust13 <- robust(model_rem_Garcia_class, cluster=Rem_Garcia_class$Study_number)
Class_model_robust13
```

```
## 
## Multivariate Meta-Analysis Model (k = 637; method: REML)
## 
## Variance Components:
## 
##             estim    sqrt  nlvls  fixed                        factor 
## sigma^2.1  0.1227  0.3503     76     no                  Study_number 
## sigma^2.2  0.0902  0.3004    350     no  Study_number/Response_number 
## 
## Test for Residual Heterogeneity:
## QE(df = 633) = 23313.2914, p-val < .0001
## 
## Number of estimates:   637
## Number of clusters:    76
## Estimates per cluster: 1-36 (mean: 8.38, median: 6)
## 
## Test of Moderators (coefficients 1:4):¹
## F(df1 = 4, df2 = 72) = 14.6626, p-val < .0001
## 
## Model Results:
## 
##                          estimate      se¹     tval¹  df¹    pval¹    ci.lb¹ 
## LnSR:ClassAsteroidea      -0.3522  0.0554   -6.3579   72   <.0001   -0.4626  
## LnSR:ClassEchinoidea      -0.0009  0.0168   -0.0511   72   0.9594   -0.0344  
## LnSR:ClassHolothuroidea   -0.1102  0.0268   -4.1065   72   0.0001   -0.1636  
## LnSR:ClassOphiuroidea      0.1362  0.1167    1.1670   72   0.2471   -0.0965  
##                            ci.ub¹      
## LnSR:ClassAsteroidea     -0.2418   *** 
## LnSR:ClassEchinoidea      0.0327       
## LnSR:ClassHolothuroidea  -0.0567   *** 
## LnSR:ClassOphiuroidea     0.3688       
## 
## ---
## Signif. codes:  0 '***' 0.001 '**' 0.01 '*' 0.05 '.' 0.1 ' ' 1
## 
## 1) results based on cluster-robust inference (var-cov estimator: CR1,
##    approx t/F-tests and confidence intervals, df: residual method)
```

```
Rem_Villalobos_class <- subset(Classdata, Study_name !="Villalobos et al. 2006")
model_rem_Villalobos_class <- rma.mv(yi = LnRR, V = Variance, mods = ~LnSR:Class - 1, test="t", random = list(~1 |Study_number/Response_number), method = "REML", data = Rem_Villalobos_class)
Class_model_robust14 <- robust(model_rem_Villalobos_class, cluster=Rem_Villalobos_class$Study_number)
Class_model_robust14
```

```
## 
## Multivariate Meta-Analysis Model (k = 658; method: REML)
## 
## Variance Components:
## 
##             estim    sqrt  nlvls  fixed                        factor 
## sigma^2.1  0.1144  0.3383     80     no                  Study_number 
## sigma^2.2  0.0896  0.2994    361     no  Study_number/Response_number 
## 
## Test for Residual Heterogeneity:
## QE(df = 654) = 24179.1460, p-val < .0001
## 
## Number of estimates:   658
## Number of clusters:    80
## Estimates per cluster: 1-36 (mean: 8.22, median: 6)
## 
## Test of Moderators (coefficients 1:4):¹
## F(df1 = 4, df2 = 76) = 14.7350, p-val < .0001
## 
## Model Results:
## 
##                          estimate      se¹     tval¹  df¹    pval¹    ci.lb¹ 
## LnSR:ClassAsteroidea      -0.3518  0.0553   -6.3629   76   <.0001   -0.4619  
## LnSR:ClassEchinoidea      -0.0066  0.0141   -0.4721   76   0.6382   -0.0346  
## LnSR:ClassHolothuroidea   -0.1100  0.0268   -4.1047   76   0.0001   -0.1634  
## LnSR:ClassOphiuroidea      0.1369  0.1165    1.1755   76   0.2435   -0.0951  
##                            ci.ub¹      
## LnSR:ClassAsteroidea     -0.2417   *** 
## LnSR:ClassEchinoidea      0.0214       
## LnSR:ClassHolothuroidea  -0.0566   *** 
## LnSR:ClassOphiuroidea     0.3689       
## 
## ---
## Signif. codes:  0 '***' 0.001 '**' 0.01 '*' 0.05 '.' 0.1 ' ' 1
## 
## 1) results based on cluster-robust inference (var-cov estimator: CR1,
##    approx t/F-tests and confidence intervals, df: residual method)
```

```
Rem_Hill_class <- subset(Classdata, Study_name !="Hill and Lawrence 2006")
model_rem_Hill_class <- rma.mv(yi = LnRR, V = Variance, mods = ~LnSR:Class - 1, test="t", random = list(~1 |Study_number/Response_number), method = "REML", data = Rem_Hill_class)
Class_model_robust15 <- robust(model_rem_Hill_class, cluster=Rem_Hill_class$Study_number)
Class_model_robust15
```

```
## 
## Multivariate Meta-Analysis Model (k = 619; method: REML)
## 
## Variance Components:
## 
##             estim    sqrt  nlvls  fixed                        factor 
## sigma^2.1  0.1165  0.3414     78     no                  Study_number 
## sigma^2.2  0.1075  0.3279    322     no  Study_number/Response_number 
## 
## Test for Residual Heterogeneity:
## QE(df = 615) = 23409.8352, p-val < .0001
## 
## Number of estimates:   619
## Number of clusters:    78
## Estimates per cluster: 1-36 (mean: 7.94, median: 6)
## 
## Test of Moderators (coefficients 1:4):¹
## F(df1 = 4, df2 = 74) = 14.7284, p-val < .0001
## 
## Model Results:
## 
##                          estimate      se¹     tval¹  df¹    pval¹    ci.lb¹ 
## LnSR:ClassAsteroidea      -0.3522  0.0553   -6.3653   74   <.0001   -0.4625  
## LnSR:ClassEchinoidea      -0.0066  0.0141   -0.4693   74   0.6402   -0.0347  
## LnSR:ClassHolothuroidea   -0.1102  0.0268   -4.1100   74   0.0001   -0.1636  
## LnSR:ClassOphiuroidea      0.1350  0.1191    1.1330   74   0.2609   -0.1024  
##                            ci.ub¹      
## LnSR:ClassAsteroidea     -0.2420   *** 
## LnSR:ClassEchinoidea      0.0215       
## LnSR:ClassHolothuroidea  -0.0568   *** 
## LnSR:ClassOphiuroidea     0.3723       
## 
## ---
## Signif. codes:  0 '***' 0.001 '**' 0.01 '*' 0.05 '.' 0.1 ' ' 1
## 
## 1) results based on cluster-robust inference (var-cov estimator: CR1,
##    approx t/F-tests and confidence intervals, df: residual method)
```

```
Rem_Detree_class <- subset(Classdata, Study_name !="Detree et al. 2020")
model_rem_Detree_class <- rma.mv(yi = LnRR, V = Variance, mods = ~LnSR:Class - 1, test="t", random = list(~1 |Study_number/Response_number), method = "REML", data = Rem_Detree_class)
Class_model_robust16 <- robust(model_rem_Detree_class, cluster=Rem_Detree_class$Study_number)
Class_model_robust16
```

```
## 
## Multivariate Meta-Analysis Model (k = 650; method: REML)
## 
## Variance Components:
## 
##             estim    sqrt  nlvls  fixed                        factor 
## sigma^2.1  0.1187  0.3446     78     no                  Study_number 
## sigma^2.2  0.0912  0.3020    353     no  Study_number/Response_number 
## 
## Test for Residual Heterogeneity:
## QE(df = 646) = 24145.6632, p-val < .0001
## 
## Number of estimates:   650
## Number of clusters:    78
## Estimates per cluster: 1-36 (mean: 8.33, median: 6)
## 
## Test of Moderators (coefficients 1:4):¹
## F(df1 = 4, df2 = 74) = 14.7290, p-val < .0001
## 
## Model Results:
## 
##                          estimate      se¹     tval¹  df¹    pval¹    ci.lb¹ 
## LnSR:ClassAsteroidea      -0.3520  0.0553   -6.3611   74   <.0001   -0.4623  
## LnSR:ClassEchinoidea      -0.0067  0.0141   -0.4753   74   0.6360   -0.0347  
## LnSR:ClassHolothuroidea   -0.1101  0.0268   -4.1064   74   0.0001   -0.1635  
## LnSR:ClassOphiuroidea      0.1364  0.1168    1.1678   74   0.2466   -0.0963  
##                            ci.ub¹      
## LnSR:ClassAsteroidea     -0.2418   *** 
## LnSR:ClassEchinoidea      0.0213       
## LnSR:ClassHolothuroidea  -0.0567   *** 
## LnSR:ClassOphiuroidea     0.3691       
## 
## ---
## Signif. codes:  0 '***' 0.001 '**' 0.01 '*' 0.05 '.' 0.1 ' ' 1
## 
## 1) results based on cluster-robust inference (var-cov estimator: CR1,
##    approx t/F-tests and confidence intervals, df: residual method)
```

```
Rem_Kuhnhold_class <- subset(Classdata, Study_name !="Kuhnhold et al. 2019")
model_rem_Kuhnhold_class <- rma.mv(yi = LnRR, V = Variance, mods = ~LnSR:Class - 1, test="t", random = list(~1 |Study_number/Response_number), method = "REML", data = Rem_Kuhnhold_class)
Class_model_robust17 <- robust(model_rem_Kuhnhold_class, cluster=Rem_Kuhnhold_class$Study_number)
Class_model_robust17
```

```
## 
## Multivariate Meta-Analysis Model (k = 646; method: REML)
## 
## Variance Components:
## 
##             estim    sqrt  nlvls  fixed                        factor 
## sigma^2.1  0.1160  0.3406     78     no                  Study_number 
## sigma^2.2  0.0819  0.2862    349     no  Study_number/Response_number 
## 
## Test for Residual Heterogeneity:
## QE(df = 642) = 23659.3242, p-val < .0001
## 
## Number of estimates:   646
## Number of clusters:    78
## Estimates per cluster: 1-36 (mean: 8.28, median: 6)
## 
## Test of Moderators (coefficients 1:4):¹
## F(df1 = 4, df2 = 74) = 14.7088, p-val < .0001
## 
## Model Results:
## 
##                          estimate      se¹     tval¹  df¹    pval¹    ci.lb¹ 
## LnSR:ClassAsteroidea      -0.3517  0.0553   -6.3564   74   <.0001   -0.4620  
## LnSR:ClassEchinoidea      -0.0066  0.0141   -0.4723   74   0.6381   -0.0347  
## LnSR:ClassHolothuroidea   -0.1113  0.0272   -4.0965   74   0.0001   -0.1655  
## LnSR:ClassOphiuroidea      0.1377  0.1153    1.1947   74   0.2360   -0.0920  
##                            ci.ub¹      
## LnSR:ClassAsteroidea     -0.2415   *** 
## LnSR:ClassEchinoidea      0.0214       
## LnSR:ClassHolothuroidea  -0.0572   *** 
## LnSR:ClassOphiuroidea     0.3674       
## 
## ---
## Signif. codes:  0 '***' 0.001 '**' 0.01 '*' 0.05 '.' 0.1 ' ' 1
## 
## 1) results based on cluster-robust inference (var-cov estimator: CR1,
##    approx t/F-tests and confidence intervals, df: residual method)
```

# Models with observations removed where the experimental temperature is lower than the future (+2.58C) mean annual temperature (MAT)

## Loading data

```
setwd('/Users/bethanlang/Documents/PhD/Literature Review')
Classdata_CC <- read_csv("Lang et al. 2022_dataset_CC2906_nometabolism.csv")
```

```
## Rows: 488 Columns: 44
## ── Column specification ────────────────────────────────────────────────────────
## Delimiter: ","
## chr  (9): Study_name, Species, Class, Lifestage, Latitude_cat, Latitude_cat2...
## dbl (35): Study_number, Year, Effect_size_id, Latitude_cont, Response_number...
## 
## ℹ Use `spec()` to retrieve the full column specification for this data.
## ℹ Specify the column types or set `show_col_types = FALSE` to quiet this message.
```

## Turning random effects into factors

```
Classdata_CC$Study_number = factor(Classdata_CC$Study_number)
Classdata_CC$Response_number = factor(Classdata_CC$Response_number)
```

## Ordering data and number of classes

```
Classdata_CC <- Classdata_CC[order(Classdata_CC$Class),]
```

## Number in each class

```
Numberclasses <- Classdata_CC %>% group_by(Class) %>% summarise(n()) 
Numberclasses$n <- Numberclasses$`n()`
```

## The model

```
Class_model_CC <- rma.mv(yi = LnRR, V = Variance, mods = ~LnSR:Class-1, test="t", random = list(~1|Study_number/Response_number), method = "REML", data = Classdata_CC)
print(Class_model_CC)
```

```
## 
## Multivariate Meta-Analysis Model (k = 488; method: REML)
## 
## Variance Components:
## 
##             estim    sqrt  nlvls  fixed                        factor 
## sigma^2.1  0.2651  0.5148     74     no                  Study_number 
## sigma^2.2  0.1245  0.3528    332     no  Study_number/Response_number 
## 
## Test for Residual Heterogeneity:
## QE(df = 484) = 14838.0360, p-val < .0001
## 
## Test of Moderators (coefficients 1:4):
## F(df1 = 4, df2 = 484) = 139.9274, p-val < .0001
## 
## Model Results:
## 
##                          estimate      se      tval   df    pval    ci.lb 
## LnSR:ClassAsteroidea      -0.7193  0.0368  -19.5409  484  <.0001  -0.7916 
## LnSR:ClassEchinoidea      -0.0099  0.0161   -0.6166  484  0.5378  -0.0416 
## LnSR:ClassHolothuroidea   -0.3267  0.0246  -13.3059  484  <.0001  -0.3750 
## LnSR:ClassOphiuroidea      0.1434  0.2176    0.6592  484  0.5101  -0.2841 
##                            ci.ub      
## LnSR:ClassAsteroidea     -0.6469  *** 
## LnSR:ClassEchinoidea      0.0217      
## LnSR:ClassHolothuroidea  -0.2785  *** 
## LnSR:ClassOphiuroidea     0.5710      
## 
## ---
## Signif. codes:  0 '***' 0.001 '**' 0.01 '*' 0.05 '.' 0.1 ' ' 1
```

## Robust model

```
Class_model_robust_CC <- robust(Class_model_CC, cluster=Classdata_CC$Study_number)
Class_model_robust_CC
```

```
## 
## Multivariate Meta-Analysis Model (k = 488; method: REML)
## 
## Variance Components:
## 
##             estim    sqrt  nlvls  fixed                        factor 
## sigma^2.1  0.2651  0.5148     74     no                  Study_number 
## sigma^2.2  0.1245  0.3528    332     no  Study_number/Response_number 
## 
## Test for Residual Heterogeneity:
## QE(df = 484) = 14838.0360, p-val < .0001
## 
## Number of estimates:   488
## Number of clusters:    74
## Estimates per cluster: 1-30 (mean: 6.59, median: 4.5)
## 
## Test of Moderators (coefficients 1:4):¹
## F(df1 = 4, df2 = 70) = 5.0141, p-val = 0.0013
## 
## Model Results:
## 
##                          estimate      se¹     tval¹  df¹    pval¹    ci.lb¹ 
## LnSR:ClassAsteroidea      -0.7193  0.1863   -3.8618   70   0.0002   -1.0907  
## LnSR:ClassEchinoidea      -0.0099  0.0469   -0.2119   70   0.8328   -0.1035  
## LnSR:ClassHolothuroidea   -0.3267  0.1628   -2.0068   70   0.0486   -0.6514  
## LnSR:ClassOphiuroidea      0.1434  0.1386    1.0348   70   0.3043   -0.1330  
##                            ci.ub¹      
## LnSR:ClassAsteroidea     -0.3478   *** 
## LnSR:ClassEchinoidea      0.0836       
## LnSR:ClassHolothuroidea  -0.0020     * 
## LnSR:ClassOphiuroidea     0.4199       
## 
## ---
## Signif. codes:  0 '***' 0.001 '**' 0.01 '*' 0.05 '.' 0.1 ' ' 1
## 
## 1) results based on cluster-robust inference (var-cov estimator: CR1,
##    approx t/F-tests and confidence intervals, df: residual method)
```

# Region

## Loading data

```
setwd('/Users/bethanlang/Documents/PhD/Literature Review')
Latitudedata <- read_csv("Lang et al. 2022_dataset_2906_nometabolism.csv")
```

```
## Rows: 658 Columns: 44
## ── Column specification ────────────────────────────────────────────────────────
## Delimiter: ","
## chr  (9): Study_name, Species, Class, Lifestage, Latitude_cat, Latitude_cat2...
## dbl (35): Study_number, Year, Effect_size_id, Latitude_cont, Response_number...
## 
## ℹ Use `spec()` to retrieve the full column specification for this data.
## ℹ Specify the column types or set `show_col_types = FALSE` to quiet this message.
```

## Turning random effects into factors

```
Latitudedata$Study_number = factor(Latitudedata$Study_number)
Latitudedata$Response_number = factor(Latitudedata$Response_number)
```

## Ordering data

```
Latitudedata <- Latitudedata[order(Latitudedata$Latitude_cat),]
```

## Number in each latitude

```
Numberlatitude <- Latitudedata %>% group_by(Latitude_cat2) %>% summarise(n()) 
Numberlatitude$n <- Numberlatitude$`n()`
```

## The model

```
Latitude_model <- rma.mv(yi = LnRR, V = Variance, mods = ~LnSR:Latitude_cat2-1,  test="t", random = list(~1|Study_number/Response_number), method = "REML", data = Latitudedata)
print(Latitude_model)
```

```
## 
## Multivariate Meta-Analysis Model (k = 658; method: REML)
## 
## Variance Components:
## 
##             estim    sqrt  nlvls  fixed                        factor 
## sigma^2.1  0.1115  0.3339     80     no                  Study_number 
## sigma^2.2  0.0889  0.2982    361     no  Study_number/Response_number 
## 
## Test for Residual Heterogeneity:
## QE(df = 654) = 25075.4058, p-val < .0001
## 
## Test of Moderators (coefficients 1:4):
## F(df1 = 4, df2 = 654) = 46.6225, p-val < .0001
## 
## Model Results:
## 
##                                             estimate      se      tval   df 
## LnSR:Latitude_cat2a. Polar (62-78°)           0.0308  0.0139    2.2242  654 
## LnSR:Latitude_cat2b. Temperate (36-53°)      -0.0588  0.0110   -5.3551  654 
## LnSR:Latitude_cat2c. Sub-tropical (26-35°)   -0.0926  0.0142   -6.5270  654 
## LnSR:Latitude_cat2d. Tropical (4-23°)        -0.0790  0.0075  -10.5006  654 
##                                               pval    ci.lb    ci.ub      
## LnSR:Latitude_cat2a. Polar (62-78°)         0.0265   0.0036   0.0581    * 
## LnSR:Latitude_cat2b. Temperate (36-53°)     <.0001  -0.0804  -0.0373  *** 
## LnSR:Latitude_cat2c. Sub-tropical (26-35°)  <.0001  -0.1205  -0.0647  *** 
## LnSR:Latitude_cat2d. Tropical (4-23°)       <.0001  -0.0937  -0.0642  *** 
## 
## ---
## Signif. codes:  0 '***' 0.001 '**' 0.01 '*' 0.05 '.' 0.1 ' ' 1
```

## Robust model

```
Latitude_model_robust <- robust(Latitude_model, cluster=Latitudedata$Study_number)
Latitude_model_robust
```

```
## 
## Multivariate Meta-Analysis Model (k = 658; method: REML)
## 
## Variance Components:
## 
##             estim    sqrt  nlvls  fixed                        factor 
## sigma^2.1  0.1115  0.3339     80     no                  Study_number 
## sigma^2.2  0.0889  0.2982    361     no  Study_number/Response_number 
## 
## Test for Residual Heterogeneity:
## QE(df = 654) = 25075.4058, p-val < .0001
## 
## Number of estimates:   658
## Number of clusters:    80
## Estimates per cluster: 1-36 (mean: 8.22, median: 6)
## 
## Test of Moderators (coefficients 1:4):¹
## F(df1 = 4, df2 = 76) = 2.8399, p-val = 0.0299
## 
## Model Results:
## 
##                                             estimate      se¹     tval¹  df¹ 
## LnSR:Latitude_cat2a. Polar (62-78°)           0.0308  0.0329    0.9363   76  
## LnSR:Latitude_cat2b. Temperate (36-53°)      -0.0588  0.0347   -1.6947   76  
## LnSR:Latitude_cat2c. Sub-tropical (26-35°)   -0.0926  0.0388   -2.3871   76  
## LnSR:Latitude_cat2d. Tropical (4-23°)        -0.0790  0.0571   -1.3830   76  
##                                               pval¹    ci.lb¹    ci.ub¹    
## LnSR:Latitude_cat2a. Polar (62-78°)         0.3521   -0.0348    0.0965     
## LnSR:Latitude_cat2b. Temperate (36-53°)     0.0942   -0.1280    0.0103   . 
## LnSR:Latitude_cat2c. Sub-tropical (26-35°)  0.0195   -0.1699   -0.0153   * 
## LnSR:Latitude_cat2d. Tropical (4-23°)       0.1707   -0.1927    0.0348     
## 
## ---
## Signif. codes:  0 '***' 0.001 '**' 0.01 '*' 0.05 '.' 0.1 ' ' 1
## 
## 1) results based on cluster-robust inference (var-cov estimator: CR1,
##    approx t/F-tests and confidence intervals, df: residual method)
```

Now trop N no longer sig.

## Checking model fit

```
res <- resid(Latitude_model_robust)
plot(fitted(Latitude_model_robust), res)
abline(0,0)
```

```
qqnorm(res)
qqline(res)
```

```
plot(density(res))
```

## Sensitivity analysis

### Influential data points

```
Latitudedata2 <- Latitudedata %>%
  arrange(LnRR)
```

```
datest_latitudecat <- Latitudedata2[-1,]
modeltest_latitudecat <- rma.mv(yi = LnRR, V = Variance, mods = ~LnSR:Latitude_cat2 - 1, test="t", random = list(~1 |Study_number/Response_number), method = "REML", data = datest_latitudecat)
Latitude_model_robust2 <- robust(modeltest_latitudecat, cluster=datest_latitudecat$Study_number)
Latitude_model_robust2
```

```
## 
## Multivariate Meta-Analysis Model (k = 657; method: REML)
## 
## Variance Components:
## 
##             estim    sqrt  nlvls  fixed                        factor 
## sigma^2.1  0.1112  0.3335     80     no                  Study_number 
## sigma^2.2  0.0889  0.2982    361     no  Study_number/Response_number 
## 
## Test for Residual Heterogeneity:
## QE(df = 653) = 24838.5371, p-val < .0001
## 
## Number of estimates:   657
## Number of clusters:    80
## Estimates per cluster: 1-36 (mean: 8.21, median: 6)
## 
## Test of Moderators (coefficients 1:4):¹
## F(df1 = 4, df2 = 76) = 2.8360, p-val = 0.0300
## 
## Model Results:
## 
##                                             estimate      se¹     tval¹  df¹ 
## LnSR:Latitude_cat2a. Polar (62-78°)           0.0308  0.0329    0.9363   76  
## LnSR:Latitude_cat2b. Temperate (36-53°)      -0.0588  0.0347   -1.6946   76  
## LnSR:Latitude_cat2c. Sub-tropical (26-35°)   -0.0926  0.0388   -2.3873   76  
## LnSR:Latitude_cat2d. Tropical (4-23°)        -0.0774  0.0562   -1.3771   76  
##                                               pval¹    ci.lb¹    ci.ub¹    
## LnSR:Latitude_cat2a. Polar (62-78°)         0.3521   -0.0348    0.0965     
## LnSR:Latitude_cat2b. Temperate (36-53°)     0.0942   -0.1280    0.0103   . 
## LnSR:Latitude_cat2c. Sub-tropical (26-35°)  0.0195   -0.1698   -0.0153   * 
## LnSR:Latitude_cat2d. Tropical (4-23°)       0.1725   -0.1893    0.0345     
## 
## ---
## Signif. codes:  0 '***' 0.001 '**' 0.01 '*' 0.05 '.' 0.1 ' ' 1
## 
## 1) results based on cluster-robust inference (var-cov estimator: CR1,
##    approx t/F-tests and confidence intervals, df: residual method)
```

```
datest_latitudecat2 <- datest_latitudecat[-1,]
modeltest_latitudecat2 <- rma.mv(yi = LnRR, V = Variance, mods = ~LnSR:Latitude_cat2 - 1, test="t", random = list(~1 |Study_number/Response_number), method = "REML", data = datest_latitudecat2)
Latitude_model_robust3 <- robust(modeltest_latitudecat2, cluster=datest_latitudecat2$Study_number)
Latitude_model_robust3
```

```
## 
## Multivariate Meta-Analysis Model (k = 656; method: REML)
## 
## Variance Components:
## 
##             estim    sqrt  nlvls  fixed                        factor 
## sigma^2.1  0.1114  0.3338     80     no                  Study_number 
## sigma^2.2  0.0886  0.2977    361     no  Study_number/Response_number 
## 
## Test for Residual Heterogeneity:
## QE(df = 652) = 24803.9355, p-val < .0001
## 
## Number of estimates:   656
## Number of clusters:    80
## Estimates per cluster: 1-36 (mean: 8.20, median: 6)
## 
## Test of Moderators (coefficients 1:4):¹
## F(df1 = 4, df2 = 76) = 2.8148, p-val = 0.0310
## 
## Model Results:
## 
##                                             estimate      se¹     tval¹  df¹ 
## LnSR:Latitude_cat2a. Polar (62-78°)           0.0308  0.0329    0.9363   76  
## LnSR:Latitude_cat2b. Temperate (36-53°)      -0.0584  0.0350   -1.6695   76  
## LnSR:Latitude_cat2c. Sub-tropical (26-35°)   -0.0926  0.0388   -2.3872   76  
## LnSR:Latitude_cat2d. Tropical (4-23°)        -0.0774  0.0562   -1.3771   76  
##                                               pval¹    ci.lb¹    ci.ub¹    
## LnSR:Latitude_cat2a. Polar (62-78°)         0.3521   -0.0348    0.0965     
## LnSR:Latitude_cat2b. Temperate (36-53°)     0.0991   -0.1280    0.0113   . 
## LnSR:Latitude_cat2c. Sub-tropical (26-35°)  0.0195   -0.1699   -0.0153   * 
## LnSR:Latitude_cat2d. Tropical (4-23°)       0.1725   -0.1893    0.0345     
## 
## ---
## Signif. codes:  0 '***' 0.001 '**' 0.01 '*' 0.05 '.' 0.1 ' ' 1
## 
## 1) results based on cluster-robust inference (var-cov estimator: CR1,
##    approx t/F-tests and confidence intervals, df: residual method)
```

```
datest_latitudecat3 <- datest_latitudecat2[-1,]
modeltest_latitudecat3 <- rma.mv(yi = LnRR, V = Variance, mods = ~LnSR:Latitude_cat2 - 1, test="t", random = list(~1 |Study_number/Response_number), method = "REML", data = datest_latitudecat3)
Latitude_model_robust4 <- robust(modeltest_latitudecat3, cluster=datest_latitudecat3$Study_number)
Latitude_model_robust4
```

```
## 
## Multivariate Meta-Analysis Model (k = 655; method: REML)
## 
## Variance Components:
## 
##             estim    sqrt  nlvls  fixed                        factor 
## sigma^2.1  0.1114  0.3338     80     no                  Study_number 
## sigma^2.2  0.0886  0.2977    361     no  Study_number/Response_number 
## 
## Test for Residual Heterogeneity:
## QE(df = 651) = 24783.5584, p-val < .0001
## 
## Number of estimates:   655
## Number of clusters:    80
## Estimates per cluster: 1-36 (mean: 8.19, median: 6)
## 
## Test of Moderators (coefficients 1:4):¹
## F(df1 = 4, df2 = 76) = 2.8142, p-val = 0.0310
## 
## Model Results:
## 
##                                             estimate      se¹     tval¹  df¹ 
## LnSR:Latitude_cat2a. Polar (62-78°)           0.0308  0.0329    0.9363   76  
## LnSR:Latitude_cat2b. Temperate (36-53°)      -0.0584  0.0350   -1.6695   76  
## LnSR:Latitude_cat2c. Sub-tropical (26-35°)   -0.0926  0.0388   -2.3872   76  
## LnSR:Latitude_cat2d. Tropical (4-23°)        -0.0772  0.0561   -1.3764   76  
##                                               pval¹    ci.lb¹    ci.ub¹    
## LnSR:Latitude_cat2a. Polar (62-78°)         0.3521   -0.0348    0.0965     
## LnSR:Latitude_cat2b. Temperate (36-53°)     0.0991   -0.1280    0.0113   . 
## LnSR:Latitude_cat2c. Sub-tropical (26-35°)  0.0195   -0.1699   -0.0153   * 
## LnSR:Latitude_cat2d. Tropical (4-23°)       0.1728   -0.1889    0.0345     
## 
## ---
## Signif. codes:  0 '***' 0.001 '**' 0.01 '*' 0.05 '.' 0.1 ' ' 1
## 
## 1) results based on cluster-robust inference (var-cov estimator: CR1,
##    approx t/F-tests and confidence intervals, df: residual method)
```

```
datest_latitudecat4 <- datest_latitudecat3[-1,]
modeltest_latitudecat4 <- rma.mv(yi = LnRR, V = Variance, mods = ~LnSR:Latitude_cat2 - 1, test="t", random = list(~1 |Study_number/Response_number), method = "REML", data = datest_latitudecat4)
Latitude_model_robust5 <- robust(modeltest_latitudecat4, cluster=datest_latitudecat4$Study_number)
Latitude_model_robust5
```

```
## 
## Multivariate Meta-Analysis Model (k = 654; method: REML)
## 
## Variance Components:
## 
##             estim    sqrt  nlvls  fixed                        factor 
## sigma^2.1  0.1114  0.3338     80     no                  Study_number 
## sigma^2.2  0.0889  0.2981    361     no  Study_number/Response_number 
## 
## Test for Residual Heterogeneity:
## QE(df = 650) = 24773.6910, p-val < .0001
## 
## Number of estimates:   654
## Number of clusters:    80
## Estimates per cluster: 1-36 (mean: 8.18, median: 6)
## 
## Test of Moderators (coefficients 1:4):¹
## F(df1 = 4, df2 = 76) = 2.8026, p-val = 0.0316
## 
## Model Results:
## 
##                                             estimate      se¹     tval¹  df¹ 
## LnSR:Latitude_cat2a. Polar (62-78°)           0.0308  0.0329    0.9363   76  
## LnSR:Latitude_cat2b. Temperate (36-53°)      -0.0584  0.0350   -1.6695   76  
## LnSR:Latitude_cat2c. Sub-tropical (26-35°)   -0.0923  0.0388   -2.3775   76  
## LnSR:Latitude_cat2d. Tropical (4-23°)        -0.0772  0.0561   -1.3763   76  
##                                               pval¹    ci.lb¹    ci.ub¹    
## LnSR:Latitude_cat2a. Polar (62-78°)         0.3521   -0.0348    0.0965     
## LnSR:Latitude_cat2b. Temperate (36-53°)     0.0991   -0.1280    0.0113   . 
## LnSR:Latitude_cat2c. Sub-tropical (26-35°)  0.0199   -0.1695   -0.0150   * 
## LnSR:Latitude_cat2d. Tropical (4-23°)       0.1728   -0.1889    0.0345     
## 
## ---
## Signif. codes:  0 '***' 0.001 '**' 0.01 '*' 0.05 '.' 0.1 ' ' 1
## 
## 1) results based on cluster-robust inference (var-cov estimator: CR1,
##    approx t/F-tests and confidence intervals, df: residual method)
```

```
datest_latitudecat5 <- datest_latitudecat4[-1,]
modeltest_latitudecat5 <- rma.mv(yi = LnRR, V = Variance, mods = ~LnSR:Latitude_cat2 - 1, test="t", random = list(~1 |Study_number/Response_number), method = "REML", data = datest_latitudecat5)
Latitude_model_robust6 <- robust(modeltest_latitudecat5, cluster=datest_latitudecat5$Study_number)
Latitude_model_robust6
```

```
## 
## Multivariate Meta-Analysis Model (k = 653; method: REML)
## 
## Variance Components:
## 
##             estim    sqrt  nlvls  fixed                        factor 
## sigma^2.1  0.1114  0.3338     80     no                  Study_number 
## sigma^2.2  0.0889  0.2981    361     no  Study_number/Response_number 
## 
## Test for Residual Heterogeneity:
## QE(df = 649) = 24756.4207, p-val < .0001
## 
## Number of estimates:   653
## Number of clusters:    80
## Estimates per cluster: 1-36 (mean: 8.16, median: 6)
## 
## Test of Moderators (coefficients 1:4):¹
## F(df1 = 4, df2 = 76) = 2.8197, p-val = 0.0308
## 
## Model Results:
## 
##                                             estimate      se¹     tval¹  df¹ 
## LnSR:Latitude_cat2a. Polar (62-78°)           0.0308  0.0329    0.9363   76  
## LnSR:Latitude_cat2b. Temperate (36-53°)      -0.0584  0.0350   -1.6695   76  
## LnSR:Latitude_cat2c. Sub-tropical (26-35°)   -0.0915  0.0382   -2.3918   76  
## LnSR:Latitude_cat2d. Tropical (4-23°)        -0.0772  0.0561   -1.3763   76  
##                                               pval¹    ci.lb¹    ci.ub¹    
## LnSR:Latitude_cat2a. Polar (62-78°)         0.3521   -0.0348    0.0965     
## LnSR:Latitude_cat2b. Temperate (36-53°)     0.0991   -0.1280    0.0113   . 
## LnSR:Latitude_cat2c. Sub-tropical (26-35°)  0.0192   -0.1677   -0.0153   * 
## LnSR:Latitude_cat2d. Tropical (4-23°)       0.1728   -0.1889    0.0345     
## 
## ---
## Signif. codes:  0 '***' 0.001 '**' 0.01 '*' 0.05 '.' 0.1 ' ' 1
## 
## 1) results based on cluster-robust inference (var-cov estimator: CR1,
##    approx t/F-tests and confidence intervals, df: residual method)
```

```
datest_latitudecat6 <- datest_latitudecat5[-1,]
modeltest_latitudecat6 <- rma.mv(yi = LnRR, V = Variance, mods = ~LnSR:Latitude_cat2 - 1, test="t", random = list(~1 |Study_number/Response_number), method = "REML", data = datest_latitudecat6)
Latitude_model_robust7 <- robust(modeltest_latitudecat6, cluster=datest_latitudecat6$Study_number)
Latitude_model_robust7
```

```
## 
## Multivariate Meta-Analysis Model (k = 652; method: REML)
## 
## Variance Components:
## 
##             estim    sqrt  nlvls  fixed                        factor 
## sigma^2.1  0.0961  0.3100     80     no                  Study_number 
## sigma^2.2  0.0895  0.2992    360     no  Study_number/Response_number 
## 
## Test for Residual Heterogeneity:
## QE(df = 648) = 24694.8202, p-val < .0001
## 
## Number of estimates:   652
## Number of clusters:    80
## Estimates per cluster: 1-36 (mean: 8.15, median: 6)
## 
## Test of Moderators (coefficients 1:4):¹
## F(df1 = 4, df2 = 76) = 2.8216, p-val = 0.0307
## 
## Model Results:
## 
##                                             estimate      se¹     tval¹  df¹ 
## LnSR:Latitude_cat2a. Polar (62-78°)           0.0309  0.0329    0.9387   76  
## LnSR:Latitude_cat2b. Temperate (36-53°)      -0.0579  0.0350   -1.6554   76  
## LnSR:Latitude_cat2c. Sub-tropical (26-35°)   -0.0913  0.0380   -2.4017   76  
## LnSR:Latitude_cat2d. Tropical (4-23°)        -0.0772  0.0560   -1.3770   76  
##                                               pval¹    ci.lb¹    ci.ub¹    
## LnSR:Latitude_cat2a. Polar (62-78°)         0.3509   -0.0347    0.0965     
## LnSR:Latitude_cat2b. Temperate (36-53°)     0.1020   -0.1275    0.0118     
## LnSR:Latitude_cat2c. Sub-tropical (26-35°)  0.0188   -0.1671   -0.0156   * 
## LnSR:Latitude_cat2d. Tropical (4-23°)       0.1725   -0.1888    0.0344     
## 
## ---
## Signif. codes:  0 '***' 0.001 '**' 0.01 '*' 0.05 '.' 0.1 ' ' 1
## 
## 1) results based on cluster-robust inference (var-cov estimator: CR1,
##    approx t/F-tests and confidence intervals, df: residual method)
```

```
datest_latitudecat7 <- datest_latitudecat6[-1,]
modeltest_latitudecat7 <- rma.mv(yi = LnRR, V = Variance, mods = ~LnSR:Latitude_cat2 - 1, test="t", random = list(~1 |Study_number/Response_number), method = "REML", data = datest_latitudecat7)
Latitude_model_robust8 <- robust(modeltest_latitudecat7, cluster=datest_latitudecat7$Study_number)
Latitude_model_robust8
```

```
## 
## Multivariate Meta-Analysis Model (k = 651; method: REML)
## 
## Variance Components:
## 
##             estim    sqrt  nlvls  fixed                        factor 
## sigma^2.1  0.0961  0.3100     80     no                  Study_number 
## sigma^2.2  0.0895  0.2992    360     no  Study_number/Response_number 
## 
## Test for Residual Heterogeneity:
## QE(df = 647) = 24547.1110, p-val < .0001
## 
## Number of estimates:   651
## Number of clusters:    80
## Estimates per cluster: 1-36 (mean: 8.14, median: 6)
## 
## Test of Moderators (coefficients 1:4):¹
## F(df1 = 4, df2 = 76) = 2.8032, p-val = 0.0315
## 
## Model Results:
## 
##                                             estimate      se¹     tval¹  df¹ 
## LnSR:Latitude_cat2a. Polar (62-78°)           0.0309  0.0329    0.9387   76  
## LnSR:Latitude_cat2b. Temperate (36-53°)      -0.0579  0.0350   -1.6554   76  
## LnSR:Latitude_cat2c. Sub-tropical (26-35°)   -0.0913  0.0380   -2.4018   76  
## LnSR:Latitude_cat2d. Tropical (4-23°)        -0.0760  0.0563   -1.3501   76  
##                                               pval¹    ci.lb¹    ci.ub¹    
## LnSR:Latitude_cat2a. Polar (62-78°)         0.3509   -0.0347    0.0965     
## LnSR:Latitude_cat2b. Temperate (36-53°)     0.1020   -0.1275    0.0118     
## LnSR:Latitude_cat2c. Sub-tropical (26-35°)  0.0188   -0.1670   -0.0156   * 
## LnSR:Latitude_cat2d. Tropical (4-23°)       0.1810   -0.1882    0.0361     
## 
## ---
## Signif. codes:  0 '***' 0.001 '**' 0.01 '*' 0.05 '.' 0.1 ' ' 1
## 
## 1) results based on cluster-robust inference (var-cov estimator: CR1,
##    approx t/F-tests and confidence intervals, df: residual method)
```

```
datest_latitudecat8 <- datest_latitudecat7[-1,]
modeltest_latitudecat8 <- rma.mv(yi = LnRR, V = Variance, mods = ~LnSR:Latitude_cat2 - 1, test="t", random = list(~1 |Study_number/Response_number), method = "REML", data = datest_latitudecat8)
Latitude_model_robust9 <- robust(modeltest_latitudecat8, cluster=datest_latitudecat8$Study_number)
Latitude_model_robust9
```

```
## 
## Multivariate Meta-Analysis Model (k = 650; method: REML)
## 
## Variance Components:
## 
##             estim    sqrt  nlvls  fixed                        factor 
## sigma^2.1  0.0864  0.2940     80     no                  Study_number 
## sigma^2.2  0.0876  0.2960    359     no  Study_number/Response_number 
## 
## Test for Residual Heterogeneity:
## QE(df = 646) = 24496.2483, p-val < .0001
## 
## Number of estimates:   650
## Number of clusters:    80
## Estimates per cluster: 1-36 (mean: 8.12, median: 6)
## 
## Test of Moderators (coefficients 1:4):¹
## F(df1 = 4, df2 = 76) = 2.7992, p-val = 0.0317
## 
## Model Results:
## 
##                                             estimate      se¹     tval¹  df¹ 
## LnSR:Latitude_cat2a. Polar (62-78°)           0.0310  0.0329    0.9409   76  
## LnSR:Latitude_cat2b. Temperate (36-53°)      -0.0572  0.0350   -1.6369   76  
## LnSR:Latitude_cat2c. Sub-tropical (26-35°)   -0.0912  0.0378   -2.4099   76  
## LnSR:Latitude_cat2d. Tropical (4-23°)        -0.0760  0.0563   -1.3507   76  
##                                               pval¹    ci.lb¹    ci.ub¹    
## LnSR:Latitude_cat2a. Polar (62-78°)         0.3497   -0.0346    0.0966     
## LnSR:Latitude_cat2b. Temperate (36-53°)     0.1058   -0.1268    0.0124     
## LnSR:Latitude_cat2c. Sub-tropical (26-35°)  0.0184   -0.1665   -0.0158   * 
## LnSR:Latitude_cat2d. Tropical (4-23°)       0.1808   -0.1880    0.0361     
## 
## ---
## Signif. codes:  0 '***' 0.001 '**' 0.01 '*' 0.05 '.' 0.1 ' ' 1
## 
## 1) results based on cluster-robust inference (var-cov estimator: CR1,
##    approx t/F-tests and confidence intervals, df: residual method)
```

```
datest_latitudecat9 <- datest_latitudecat8[-1,]
modeltest_latitudecat9 <- rma.mv(yi = LnRR, V = Variance, mods = ~LnSR:Latitude_cat2 - 1, test="t", random = list(~1 |Study_number/Response_number), method = "REML", data = datest_latitudecat9)
Latitude_model_robust10 <- robust(modeltest_latitudecat9, cluster=datest_latitudecat9$Study_number)
Latitude_model_robust10
```

```
## 
## Multivariate Meta-Analysis Model (k = 649; method: REML)
## 
## Variance Components:
## 
##             estim    sqrt  nlvls  fixed                        factor 
## sigma^2.1  0.0856  0.2926     80     no                  Study_number 
## sigma^2.2  0.0873  0.2954    359     no  Study_number/Response_number 
## 
## Test for Residual Heterogeneity:
## QE(df = 645) = 24466.7828, p-val < .0001
## 
## Number of estimates:   649
## Number of clusters:    80
## Estimates per cluster: 1-36 (mean: 8.11, median: 6)
## 
## Test of Moderators (coefficients 1:4):¹
## F(df1 = 4, df2 = 76) = 2.7949, p-val = 0.0319
## 
## Model Results:
## 
##                                             estimate      se¹     tval¹  df¹ 
## LnSR:Latitude_cat2a. Polar (62-78°)           0.0310  0.0329    0.9411   76  
## LnSR:Latitude_cat2b. Temperate (36-53°)      -0.0572  0.0350   -1.6364   76  
## LnSR:Latitude_cat2c. Sub-tropical (26-35°)   -0.0907  0.0377   -2.4066   76  
## LnSR:Latitude_cat2d. Tropical (4-23°)        -0.0760  0.0562   -1.3507   76  
##                                               pval¹    ci.lb¹    ci.ub¹    
## LnSR:Latitude_cat2a. Polar (62-78°)         0.3496   -0.0346    0.0966     
## LnSR:Latitude_cat2b. Temperate (36-53°)     0.1059   -0.1268    0.0124     
## LnSR:Latitude_cat2c. Sub-tropical (26-35°)  0.0185   -0.1657   -0.0156   * 
## LnSR:Latitude_cat2d. Tropical (4-23°)       0.1808   -0.1880    0.0360     
## 
## ---
## Signif. codes:  0 '***' 0.001 '**' 0.01 '*' 0.05 '.' 0.1 ' ' 1
## 
## 1) results based on cluster-robust inference (var-cov estimator: CR1,
##    approx t/F-tests and confidence intervals, df: residual method)
```

```
datest_latitudecat10 <- datest_latitudecat9[-649,]
modeltest_latitudecat10 <- rma.mv(yi = LnRR, V = Variance, mods = ~LnSR:Latitude_cat2 - 1, test="t", random = list(~1 |Study_number/Response_number), method = "REML", data = datest_latitudecat10)
Latitude_model_robust11 <- robust(modeltest_latitudecat10, cluster=datest_latitudecat10$Study_number)
Latitude_model_robust11
```

```
## 
## Multivariate Meta-Analysis Model (k = 648; method: REML)
## 
## Variance Components:
## 
##             estim    sqrt  nlvls  fixed                        factor 
## sigma^2.1  0.0837  0.2893     80     no                  Study_number 
## sigma^2.2  0.0873  0.2955    359     no  Study_number/Response_number 
## 
## Test for Residual Heterogeneity:
## QE(df = 644) = 24459.5663, p-val < .0001
## 
## Number of estimates:   648
## Number of clusters:    80
## Estimates per cluster: 1-36 (mean: 8.10, median: 6)
## 
## Test of Moderators (coefficients 1:4):¹
## F(df1 = 4, df2 = 76) = 2.7972, p-val = 0.0318
## 
## Model Results:
## 
##                                             estimate      se¹     tval¹  df¹ 
## LnSR:Latitude_cat2a. Polar (62-78°)           0.0310  0.0329    0.9416   76  
## LnSR:Latitude_cat2b. Temperate (36-53°)      -0.0572  0.0350   -1.6349   76  
## LnSR:Latitude_cat2c. Sub-tropical (26-35°)   -0.0908  0.0377   -2.4092   76  
## LnSR:Latitude_cat2d. Tropical (4-23°)        -0.0760  0.0562   -1.3509   76  
##                                               pval¹    ci.lb¹    ci.ub¹    
## LnSR:Latitude_cat2a. Polar (62-78°)         0.3494   -0.0346    0.0966     
## LnSR:Latitude_cat2b. Temperate (36-53°)     0.1062   -0.1268    0.0125     
## LnSR:Latitude_cat2c. Sub-tropical (26-35°)  0.0184   -0.1658   -0.0157   * 
## LnSR:Latitude_cat2d. Tropical (4-23°)       0.1808   -0.1880    0.0360     
## 
## ---
## Signif. codes:  0 '***' 0.001 '**' 0.01 '*' 0.05 '.' 0.1 ' ' 1
## 
## 1) results based on cluster-robust inference (var-cov estimator: CR1,
##    approx t/F-tests and confidence intervals, df: residual method)
```

## Removing papers that contribute with 2 or more studies.

```
More1study_latitudecat <- Latitudedata %>% group_by(Study_name) %>% summarise(unique(Study_number)) %>% summarise(n())
```

```
## Warning: Returning more (or less) than 1 row per `summarise()` group was deprecated in
## dplyr 1.1.0.
## ℹ Please use `reframe()` instead.
## ℹ When switching from `summarise()` to `reframe()`, remember that `reframe()`
##   always returns an ungrouped data frame and adjust accordingly.
## Call `lifecycle::last_lifecycle_warnings()` to see where this warning was
## generated.
```

```
## `summarise()` has grouped output by 'Study_name'. You can override using the
## `.groups` argument.
```

```
More1study_latitudecat$n <- More1study_latitudecat$`n()`
subset(More1study_latitudecat, n >1)
```

```
## # A tibble: 6 × 3
##   Study_name                     `n()`     n
##   <chr>                          <int> <int>
## 1 Benitez Villalobos et al. 2006     3     3
## 2 Detree et al. 2020                 2     2
## 3 Garcia et al. 2018                 4     4
## 4 Hill and Lawrence 2006             2     2
## 5 Kuhnhold et al. 2019               2     2
## 6 Rupp 1973                          6     6
```

```
Rem_Rupp_latitudecat <- subset(Latitudedata, Study_name !="Rupp 1973")
model_rem_Rupp_latitudecat <- rma.mv(yi = LnRR, V = Variance, mods = ~LnSR:Latitude_cat2 - 1, test="t", random = list(~1 |Study_number/Response_number), method = "REML", data = Rem_Rupp_latitudecat)
Latitude_model_robust12 <- robust(model_rem_Rupp_latitudecat, cluster=Rem_Rupp_latitudecat$Study_number)
Latitude_model_robust12
```

```
## 
## Multivariate Meta-Analysis Model (k = 630; method: REML)
## 
## Variance Components:
## 
##             estim    sqrt  nlvls  fixed                        factor 
## sigma^2.1  0.1338  0.3658     74     no                  Study_number 
## sigma^2.2  0.0912  0.3020    351     no  Study_number/Response_number 
## 
## Test for Residual Heterogeneity:
## QE(df = 626) = 21865.0516, p-val < .0001
## 
## Number of estimates:   630
## Number of clusters:    74
## Estimates per cluster: 1-36 (mean: 8.51, median: 6)
## 
## Test of Moderators (coefficients 1:4):¹
## F(df1 = 4, df2 = 70) = 3.6596, p-val = 0.0092
## 
## Model Results:
## 
##                                             estimate      se¹     tval¹  df¹ 
## LnSR:Latitude_cat2a. Polar (62-78°)           0.0308  0.0330    0.9312   70  
## LnSR:Latitude_cat2b. Temperate (36-53°)      -0.0590  0.0348   -1.6961   70  
## LnSR:Latitude_cat2c. Sub-tropical (26-35°)   -0.0928  0.0391   -2.3701   70  
## LnSR:Latitude_cat2d. Tropical (4-23°)        -0.2496  0.1087   -2.2972   70  
##                                               pval¹    ci.lb¹    ci.ub¹    
## LnSR:Latitude_cat2a. Polar (62-78°)         0.3549   -0.0351    0.0966     
## LnSR:Latitude_cat2b. Temperate (36-53°)     0.0943   -0.1284    0.0104   . 
## LnSR:Latitude_cat2c. Sub-tropical (26-35°)  0.0205   -0.1709   -0.0147   * 
## LnSR:Latitude_cat2d. Tropical (4-23°)       0.0246   -0.4664   -0.0329   * 
## 
## ---
## Signif. codes:  0 '***' 0.001 '**' 0.01 '*' 0.05 '.' 0.1 ' ' 1
## 
## 1) results based on cluster-robust inference (var-cov estimator: CR1,
##    approx t/F-tests and confidence intervals, df: residual method)
```

```
Rem_Garcia_latitudecat <- subset(Latitudedata, Study_name !="Garcia et al. 2018")
model_rem_Garcia_latitudecat <- rma.mv(yi = LnRR, V = Variance, mods = ~LnSR:Latitude_cat2 - 1, test="t", random = list(~1 |Study_number/Response_number), method = "REML", data = Rem_Garcia_latitudecat)
Latitude_model_robust13 <- robust(model_rem_Garcia_latitudecat, cluster=Rem_Garcia_latitudecat$Study_number)
Latitude_model_robust13
```

```
## 
## Multivariate Meta-Analysis Model (k = 637; method: REML)
## 
## Variance Components:
## 
##             estim    sqrt  nlvls  fixed                        factor 
## sigma^2.1  0.1201  0.3465     76     no                  Study_number 
## sigma^2.2  0.0895  0.2992    350     no  Study_number/Response_number 
## 
## Test for Residual Heterogeneity:
## QE(df = 633) = 24647.3100, p-val < .0001
## 
## Number of estimates:   637
## Number of clusters:    76
## Estimates per cluster: 1-36 (mean: 8.38, median: 6)
## 
## Test of Moderators (coefficients 1:4):¹
## F(df1 = 4, df2 = 72) = 2.2052, p-val = 0.0769
## 
## Model Results:
## 
##                                             estimate      se¹     tval¹  df¹ 
## LnSR:Latitude_cat2a. Polar (62-78°)           0.0308  0.0330    0.9337   72  
## LnSR:Latitude_cat2b. Temperate (36-53°)      -0.0589  0.0348   -1.6946   72  
## LnSR:Latitude_cat2c. Sub-tropical (26-35°)   -0.1209  0.0679   -1.7808   72  
## LnSR:Latitude_cat2d. Tropical (4-23°)        -0.0790  0.0572   -1.3807   72  
##                                               pval¹    ci.lb¹   ci.ub¹    
## LnSR:Latitude_cat2a. Polar (62-78°)         0.3536   -0.0350   0.0966     
## LnSR:Latitude_cat2b. Temperate (36-53°)     0.0945   -0.1282   0.0104   . 
## LnSR:Latitude_cat2c. Sub-tropical (26-35°)  0.0792   -0.2562   0.0144   . 
## LnSR:Latitude_cat2d. Tropical (4-23°)       0.1716   -0.1930   0.0351     
## 
## ---
## Signif. codes:  0 '***' 0.001 '**' 0.01 '*' 0.05 '.' 0.1 ' ' 1
## 
## 1) results based on cluster-robust inference (var-cov estimator: CR1,
##    approx t/F-tests and confidence intervals, df: residual method)
```

```
Rem_Villalobos_latitudecat <- subset(Latitudedata, Study_name !="Villalobos et al. 2006")
model_rem_Villalobos_latitudecat <- rma.mv(yi = LnRR, V = Variance, mods = ~LnSR:Latitude_cat2 - 1, test="t", random = list(~1 |Study_number/Response_number), method = "REML", data = Rem_Villalobos_latitudecat)
Latitude_model_robust14 <- robust(model_rem_Villalobos_latitudecat, cluster=Rem_Villalobos_latitudecat$Study_number)
Latitude_model_robust14
```

```
## 
## Multivariate Meta-Analysis Model (k = 658; method: REML)
## 
## Variance Components:
## 
##             estim    sqrt  nlvls  fixed                        factor 
## sigma^2.1  0.1115  0.3339     80     no                  Study_number 
## sigma^2.2  0.0889  0.2982    361     no  Study_number/Response_number 
## 
## Test for Residual Heterogeneity:
## QE(df = 654) = 25075.4058, p-val < .0001
## 
## Number of estimates:   658
## Number of clusters:    80
## Estimates per cluster: 1-36 (mean: 8.22, median: 6)
## 
## Test of Moderators (coefficients 1:4):¹
## F(df1 = 4, df2 = 76) = 2.8399, p-val = 0.0299
## 
## Model Results:
## 
##                                             estimate      se¹     tval¹  df¹ 
## LnSR:Latitude_cat2a. Polar (62-78°)           0.0308  0.0329    0.9363   76  
## LnSR:Latitude_cat2b. Temperate (36-53°)      -0.0588  0.0347   -1.6947   76  
## LnSR:Latitude_cat2c. Sub-tropical (26-35°)   -0.0926  0.0388   -2.3871   76  
## LnSR:Latitude_cat2d. Tropical (4-23°)        -0.0790  0.0571   -1.3830   76  
##                                               pval¹    ci.lb¹    ci.ub¹    
## LnSR:Latitude_cat2a. Polar (62-78°)         0.3521   -0.0348    0.0965     
## LnSR:Latitude_cat2b. Temperate (36-53°)     0.0942   -0.1280    0.0103   . 
## LnSR:Latitude_cat2c. Sub-tropical (26-35°)  0.0195   -0.1699   -0.0153   * 
## LnSR:Latitude_cat2d. Tropical (4-23°)       0.1707   -0.1927    0.0348     
## 
## ---
## Signif. codes:  0 '***' 0.001 '**' 0.01 '*' 0.05 '.' 0.1 ' ' 1
## 
## 1) results based on cluster-robust inference (var-cov estimator: CR1,
##    approx t/F-tests and confidence intervals, df: residual method)
```

```
Rem_Hill_latitudecat <- subset(Latitudedata, Study_name !="Hill and Lawrence 2006")
model_rem_Hill_latitudecat <- rma.mv(yi = LnRR, V = Variance, mods = ~LnSR:Latitude_cat2 - 1, test="t", random = list(~1 |Study_number/Response_number), method = "REML", data = Rem_Hill_latitudecat)
Latitude_model_robust15 <- robust(model_rem_Hill_latitudecat, cluster=Rem_Hill_latitudecat$Study_number)
Latitude_model_robust15
```

```
## 
## Multivariate Meta-Analysis Model (k = 619; method: REML)
## 
## Variance Components:
## 
##             estim    sqrt  nlvls  fixed                        factor 
## sigma^2.1  0.1133  0.3366     78     no                  Study_number 
## sigma^2.2  0.1065  0.3264    322     no  Study_number/Response_number 
## 
## Test for Residual Heterogeneity:
## QE(df = 615) = 24750.2752, p-val < .0001
## 
## Number of estimates:   619
## Number of clusters:    78
## Estimates per cluster: 1-36 (mean: 7.94, median: 6)
## 
## Test of Moderators (coefficients 1:4):¹
## F(df1 = 4, df2 = 74) = 2.8103, p-val = 0.0314
## 
## Model Results:
## 
##                                             estimate      se¹     tval¹  df¹ 
## LnSR:Latitude_cat2a. Polar (62-78°)           0.0308  0.0330    0.9334   74  
## LnSR:Latitude_cat2b. Temperate (36-53°)      -0.0589  0.0348   -1.6940   74  
## LnSR:Latitude_cat2c. Sub-tropical (26-35°)   -0.0933  0.0395   -2.3645   74  
## LnSR:Latitude_cat2d. Tropical (4-23°)        -0.0790  0.0571   -1.3817   74  
##                                               pval¹    ci.lb¹    ci.ub¹    
## LnSR:Latitude_cat2a. Polar (62-78°)         0.3537   -0.0349    0.0965     
## LnSR:Latitude_cat2b. Temperate (36-53°)     0.0945   -0.1282    0.0104   . 
## LnSR:Latitude_cat2c. Sub-tropical (26-35°)  0.0207   -0.1720   -0.0147   * 
## LnSR:Latitude_cat2d. Tropical (4-23°)       0.1712   -0.1928    0.0349     
## 
## ---
## Signif. codes:  0 '***' 0.001 '**' 0.01 '*' 0.05 '.' 0.1 ' ' 1
## 
## 1) results based on cluster-robust inference (var-cov estimator: CR1,
##    approx t/F-tests and confidence intervals, df: residual method)
```

```
Rem_Detree_latitudecat <- subset(Latitudedata, Study_name !="Detree et al. 2020")
model_rem_Detree_latitudecat <- rma.mv(yi = LnRR, V = Variance, mods = ~LnSR:Latitude_cat2 - 1, test="t", random = list(~1 |Study_number/Response_number), method = "REML", data = Rem_Detree_latitudecat)
Latitude_model_robust16 <- robust(model_rem_Detree_latitudecat, cluster=Rem_Detree_latitudecat$Study_number)
Latitude_model_robust16
```

```
## 
## Multivariate Meta-Analysis Model (k = 650; method: REML)
## 
## Variance Components:
## 
##             estim    sqrt  nlvls  fixed                        factor 
## sigma^2.1  0.1152  0.3394     78     no                  Study_number 
## sigma^2.2  0.0905  0.3009    353     no  Study_number/Response_number 
## 
## Test for Residual Heterogeneity:
## QE(df = 646) = 25050.4953, p-val < .0001
## 
## Number of estimates:   650
## Number of clusters:    78
## Estimates per cluster: 1-36 (mean: 8.33, median: 6)
## 
## Test of Moderators (coefficients 1:4):¹
## F(df1 = 4, df2 = 74) = 2.8365, p-val = 0.0302
## 
## Model Results:
## 
##                                             estimate      se¹     tval¹  df¹ 
## LnSR:Latitude_cat2a. Polar (62-78°)           0.0308  0.0330    0.9349   74  
## LnSR:Latitude_cat2b. Temperate (36-53°)      -0.0592  0.0349   -1.6981   74  
## LnSR:Latitude_cat2c. Sub-tropical (26-35°)   -0.0926  0.0389   -2.3831   74  
## LnSR:Latitude_cat2d. Tropical (4-23°)        -0.0790  0.0571   -1.3819   74  
##                                               pval¹    ci.lb¹    ci.ub¹    
## LnSR:Latitude_cat2a. Polar (62-78°)         0.3529   -0.0349    0.0965     
## LnSR:Latitude_cat2b. Temperate (36-53°)     0.0937   -0.1287    0.0103   . 
## LnSR:Latitude_cat2c. Sub-tropical (26-35°)  0.0197   -0.1701   -0.0152   * 
## LnSR:Latitude_cat2d. Tropical (4-23°)       0.1712   -0.1928    0.0349     
## 
## ---
## Signif. codes:  0 '***' 0.001 '**' 0.01 '*' 0.05 '.' 0.1 ' ' 1
## 
## 1) results based on cluster-robust inference (var-cov estimator: CR1,
##    approx t/F-tests and confidence intervals, df: residual method)
```

```
Rem_Kuhnhold_latitudecat <- subset(Latitudedata, Study_name !="Kuhnhold et al. 2019")
model_rem_Kuhnhold_latitudecat <- rma.mv(yi = LnRR, V = Variance, mods = ~LnSR:Latitude_cat2 - 1, test="t", random = list(~1 |Study_number/Response_number), method = "REML", data = Rem_Kuhnhold_latitudecat)
Latitude_model_robust17 <- robust(model_rem_Kuhnhold_latitudecat, cluster=Rem_Kuhnhold_latitudecat$Study_number)
Latitude_model_robust17
```

```
## 
## Multivariate Meta-Analysis Model (k = 646; method: REML)
## 
## Variance Components:
## 
##             estim    sqrt  nlvls  fixed                        factor 
## sigma^2.1  0.1138  0.3373     78     no                  Study_number 
## sigma^2.2  0.0812  0.2850    349     no  Study_number/Response_number 
## 
## Test for Residual Heterogeneity:
## QE(df = 642) = 24584.2892, p-val < .0001
## 
## Number of estimates:   646
## Number of clusters:    78
## Estimates per cluster: 1-36 (mean: 8.28, median: 6)
## 
## Test of Moderators (coefficients 1:4):¹
## F(df1 = 4, df2 = 74) = 2.8384, p-val = 0.0301
## 
## Model Results:
## 
##                                             estimate      se¹     tval¹  df¹ 
## LnSR:Latitude_cat2a. Polar (62-78°)           0.0309  0.0330    0.9363   74  
## LnSR:Latitude_cat2b. Temperate (36-53°)      -0.0590  0.0348   -1.6958   74  
## LnSR:Latitude_cat2c. Sub-tropical (26-35°)   -0.0926  0.0388   -2.3857   74  
## LnSR:Latitude_cat2d. Tropical (4-23°)        -0.0792  0.0573   -1.3820   74  
##                                               pval¹    ci.lb¹    ci.ub¹    
## LnSR:Latitude_cat2a. Polar (62-78°)         0.3522   -0.0348    0.0966     
## LnSR:Latitude_cat2b. Temperate (36-53°)     0.0941   -0.1283    0.0103   . 
## LnSR:Latitude_cat2c. Sub-tropical (26-35°)  0.0196   -0.1699   -0.0153   * 
## LnSR:Latitude_cat2d. Tropical (4-23°)       0.1711   -0.1935    0.0350     
## 
## ---
## Signif. codes:  0 '***' 0.001 '**' 0.01 '*' 0.05 '.' 0.1 ' ' 1
## 
## 1) results based on cluster-robust inference (var-cov estimator: CR1,
##    approx t/F-tests and confidence intervals, df: residual method)
```

# Models with observations removed where the experimental temperature is lower than the future (+2.58C) mean annual temperature (MAT)

## Loading data

```
setwd('/Users/bethanlang/Documents/PhD/Literature Review')
Latitudedata_CC <- read_csv("Lang et al. 2022_dataset_CC2906_nometabolism.csv")
```

```
## Rows: 488 Columns: 44
## ── Column specification ────────────────────────────────────────────────────────
## Delimiter: ","
## chr  (9): Study_name, Species, Class, Lifestage, Latitude_cat, Latitude_cat2...
## dbl (35): Study_number, Year, Effect_size_id, Latitude_cont, Response_number...
## 
## ℹ Use `spec()` to retrieve the full column specification for this data.
## ℹ Specify the column types or set `show_col_types = FALSE` to quiet this message.
```

## Turning random effects into factors

```
Latitudedata_CC$Study_number = factor(Latitudedata_CC$Study_number)
Latitudedata_CC$Response_number = factor(Latitudedata_CC$Response_number)
```

## Ordering data

```
Latitudedata_CC <- Latitudedata_CC[order(Latitudedata_CC$Latitude_cat),]
```

## Number in each latitude

```
Numberlatitude <- Latitudedata_CC %>% group_by(Latitude_cat2) %>% summarise(n()) 
Numberlatitude$n <- Numberlatitude$`n()`
```

## The model

```
Latitude_model_CC <- rma.mv(yi = LnRR, V = Variance, mods = ~LnSR:Latitude_cat2-1, test="t", random = list(~1|Study_number/Response_number), method = "REML", data = Latitudedata_CC)
print(Latitude_model_CC)
```

```
## 
## Multivariate Meta-Analysis Model (k = 488; method: REML)
## 
## Variance Components:
## 
##             estim    sqrt  nlvls  fixed                        factor 
## sigma^2.1  0.2057  0.4535     74     no                  Study_number 
## sigma^2.2  0.1229  0.3505    332     no  Study_number/Response_number 
## 
## Test for Residual Heterogeneity:
## QE(df = 484) = 14998.9357, p-val < .0001
## 
## Test of Moderators (coefficients 1:4):
## F(df1 = 4, df2 = 484) = 82.6976, p-val < .0001
## 
## Model Results:
## 
##                                 estimate      se      tval   df    pval 
## LnSR:Latitude_cat2Polar           0.0847  0.0362    2.3373  484  0.0198 
## LnSR:Latitude_cat2Sub-tropical   -0.0952  0.0283   -3.3625  484  0.0008 
## LnSR:Latitude_cat2Temperate      -0.1672  0.0184   -9.0807  484  <.0001 
## LnSR:Latitude_cat2Tropical       -0.4082  0.0268  -15.2171  484  <.0001 
##                                   ci.lb    ci.ub      
## LnSR:Latitude_cat2Polar          0.0135   0.1558    * 
## LnSR:Latitude_cat2Sub-tropical  -0.1508  -0.0396  *** 
## LnSR:Latitude_cat2Temperate     -0.2033  -0.1310  *** 
## LnSR:Latitude_cat2Tropical      -0.4609  -0.3555  *** 
## 
## ---
## Signif. codes:  0 '***' 0.001 '**' 0.01 '*' 0.05 '.' 0.1 ' ' 1
```

## Robust model

```
Latitude_model_robust_CC <- robust(Latitude_model_CC, cluster=Latitudedata_CC$Study_number)
Latitude_model_robust_CC
```

```
## 
## Multivariate Meta-Analysis Model (k = 488; method: REML)
## 
## Variance Components:
## 
##             estim    sqrt  nlvls  fixed                        factor 
## sigma^2.1  0.2057  0.4535     74     no                  Study_number 
## sigma^2.2  0.1229  0.3505    332     no  Study_number/Response_number 
## 
## Test for Residual Heterogeneity:
## QE(df = 484) = 14998.9357, p-val < .0001
## 
## Number of estimates:   488
## Number of clusters:    74
## Estimates per cluster: 1-30 (mean: 6.59, median: 4.5)
## 
## Test of Moderators (coefficients 1:4):¹
## F(df1 = 4, df2 = 70) = 1.6212, p-val = 0.1786
## 
## Model Results:
## 
##                                 estimate      se¹     tval¹  df¹    pval¹ 
## LnSR:Latitude_cat2Polar           0.0847  0.0781    1.0837   70   0.2822  
## LnSR:Latitude_cat2Sub-tropical   -0.0952  0.1086   -0.8767   70   0.3836  
## LnSR:Latitude_cat2Temperate      -0.1672  0.1225   -1.3645   70   0.1768  
## LnSR:Latitude_cat2Tropical       -0.4082  0.2494   -1.6370   70   0.1061  
##                                   ci.lb¹   ci.ub¹    
## LnSR:Latitude_cat2Polar         -0.0711   0.2405     
## LnSR:Latitude_cat2Sub-tropical  -0.3118   0.1214     
## LnSR:Latitude_cat2Temperate     -0.4115   0.0772     
## LnSR:Latitude_cat2Tropical      -0.9055   0.0891     
## 
## ---
## Signif. codes:  0 '***' 0.001 '**' 0.01 '*' 0.05 '.' 0.1 ' ' 1
## 
## 1) results based on cluster-robust inference (var-cov estimator: CR1,
##    approx t/F-tests and confidence intervals, df: residual method)
```

# Habituation time

## Loading data check correct datasets

```
setwd('/Users/bethanlang/Documents/PhD/Literature Review')
Habituationtimedata <- read_csv("Lang et al. 2022_dataset_2906_nometabolism.csv")
```

```
## Rows: 658 Columns: 44
## ── Column specification ────────────────────────────────────────────────────────
## Delimiter: ","
## chr  (9): Study_name, Species, Class, Lifestage, Latitude_cat, Latitude_cat2...
## dbl (35): Study_number, Year, Effect_size_id, Latitude_cont, Response_number...
## 
## ℹ Use `spec()` to retrieve the full column specification for this data.
## ℹ Specify the column types or set `show_col_types = FALSE` to quiet this message.
```

## Ordering data

```
Habituationtimedata <- Habituationtimedata[order(Habituationtimedata$Habituation_time),]
```

## Turning random effects into factors

```
Habituationtimedata$Study_number = factor(Habituationtimedata$Study_number)
Habituationtimedata$Response_number = factor(Habituationtimedata$Response_number)
```

## The model

```
Habituationtime_model <- rma.mv(yi = LnRR, V = Variance, mods = ~Habituation_time-1, test="t", random = list(~1|Study_number/Response_number), method = "REML", data = Habituationtimedata)
```

```
## Warning: 299 rows with NAs omitted from model fitting.
```

```
print(Habituationtime_model)
```

```
## 
## Multivariate Meta-Analysis Model (k = 359; method: REML)
## 
## Variance Components:
## 
##             estim    sqrt  nlvls  fixed                        factor 
## sigma^2.1  0.0549  0.2343     35     no                  Study_number 
## sigma^2.2  0.1093  0.3307    178     no  Study_number/Response_number 
## 
## Test for Residual Heterogeneity:
## QE(df = 358) = 25001.8438, p-val < .0001
## 
## Model Results:
## 
##                   estimate      se    tval   df    pval    ci.lb   ci.ub    
## Habituation_time    0.0032  0.0024  1.3358  358  0.1825  -0.0015  0.0078    
## 
## ---
## Signif. codes:  0 '***' 0.001 '**' 0.01 '*' 0.05 '.' 0.1 ' ' 1
```

## Robust model

```
Habituationtime_model_robust <- robust(Habituationtime_model, cluster=Habituationtimedata$Study_number)
Habituationtime_model_robust
```

```
## 
## Multivariate Meta-Analysis Model (k = 359; method: REML)
## 
## Variance Components:
## 
##             estim    sqrt  nlvls  fixed                        factor 
## sigma^2.1  0.0549  0.2343     35     no                  Study_number 
## sigma^2.2  0.1093  0.3307    178     no  Study_number/Response_number 
## 
## Test for Residual Heterogeneity:
## QE(df = 358) = 25001.8438, p-val < .0001
## 
## Number of estimates:   359
## Number of clusters:    35
## Estimates per cluster: 2-36 (mean: 10.26, median: 8)
## 
## Model Results:
## 
##                   estimate      se¹    tval¹  df¹    pval¹    ci.lb¹   ci.ub¹ 
## Habituation_time    0.0032  0.0039   0.8235   34   0.4159   -0.0047   0.0110  
##                     
## Habituation_time    
## 
## ---
## Signif. codes:  0 '***' 0.001 '**' 0.01 '*' 0.05 '.' 0.1 ' ' 1
## 
## 1) results based on cluster-robust inference (var-cov estimator: CR1,
##    approx t/F-tests and confidence intervals, df: residual method)
```

## Checking model fit

```
res <- resid(Habituationtime_model_robust)
plot(fitted(Habituationtime_model_robust), res)
abline(0,0)
```

```
qqnorm(res)
qqline(res)
```

```
plot(density(res))
```

# Models with observations removed where the experimental temperature is lower than the future (+2.58C) mean annual temperature (MAT)

## Loading data

```
setwd('/Users/bethanlang/Documents/PhD/Literature Review')
Habituationtimedata_CC <- read_csv("Lang et al. 2022_dataset_CC2906_nometabolism.csv")
```

```
## Rows: 488 Columns: 44
## ── Column specification ────────────────────────────────────────────────────────
## Delimiter: ","
## chr  (9): Study_name, Species, Class, Lifestage, Latitude_cat, Latitude_cat2...
## dbl (35): Study_number, Year, Effect_size_id, Latitude_cont, Response_number...
## 
## ℹ Use `spec()` to retrieve the full column specification for this data.
## ℹ Specify the column types or set `show_col_types = FALSE` to quiet this message.
```

## Turning random effects into factors

```
Habituationtimedata_CC$Study_number = factor(Habituationtimedata_CC$Study_number)
Habituationtimedata_CC$Response_number = factor(Habituationtimedata_CC$Response_number)
```

## Ordering data

```
Habituationtimedata_CC <- Habituationtimedata_CC[order(Habituationtimedata_CC$Habituation_time),]
```

## The model

```
Habituationtime_model_CC <- rma.mv(yi = LnRR, V = Variance, mods = ~Habituation_time-1, test="t", random = list(~1|Study_number/Response_number), method = "REML", data = Habituationtimedata_CC)
```

```
## Warning: 229 rows with NAs omitted from model fitting.
```

```
print(Habituationtime_model_CC)
```

```
## 
## Multivariate Meta-Analysis Model (k = 259; method: REML)
## 
## Variance Components:
## 
##             estim    sqrt  nlvls  fixed                        factor 
## sigma^2.1  0.0824  0.2870     32     no                  Study_number 
## sigma^2.2  0.1461  0.3822    160     no  Study_number/Response_number 
## 
## Test for Residual Heterogeneity:
## QE(df = 258) = 8746.4777, p-val < .0001
## 
## Model Results:
## 
##                   estimate      se    tval   df    pval    ci.lb   ci.ub    
## Habituation_time    0.0025  0.0029  0.8645  258  0.3881  -0.0032  0.0081    
## 
## ---
## Signif. codes:  0 '***' 0.001 '**' 0.01 '*' 0.05 '.' 0.1 ' ' 1
```

## Robust model

```
Habituationtime_model_robust_CC <- robust(Habituationtime_model_CC, cluster=Habituationtimedata_CC$Study_number)
Habituationtime_model_robust_CC
```

```
## 
## Multivariate Meta-Analysis Model (k = 259; method: REML)
## 
## Variance Components:
## 
##             estim    sqrt  nlvls  fixed                        factor 
## sigma^2.1  0.0824  0.2870     32     no                  Study_number 
## sigma^2.2  0.1461  0.3822    160     no  Study_number/Response_number 
## 
## Test for Residual Heterogeneity:
## QE(df = 258) = 8746.4777, p-val < .0001
## 
## Number of estimates:   259
## Number of clusters:    32
## Estimates per cluster: 1-30 (mean: 8.09, median: 5.5)
## 
## Model Results:
## 
##                   estimate      se¹    tval¹  df¹    pval¹    ci.lb¹   ci.ub¹ 
## Habituation_time    0.0025  0.0042   0.5929   31   0.5576   -0.0061   0.0110  
##                     
## Habituation_time    
## 
## ---
## Signif. codes:  0 '***' 0.001 '**' 0.01 '*' 0.05 '.' 0.1 ' ' 1
## 
## 1) results based on cluster-robust inference (var-cov estimator: CR1,
##    approx t/F-tests and confidence intervals, df: residual method)
```

# Warming rate (all data)

## Loading data

```
setwd('/Users/bethanlang/Documents/PhD/Literature Review')
Warmingratedata <- read_csv("Lang et al. 2022_dataset_2906_nometabolism.csv")
```

```
## Rows: 658 Columns: 44
## ── Column specification ────────────────────────────────────────────────────────
## Delimiter: ","
## chr  (9): Study_name, Species, Class, Lifestage, Latitude_cat, Latitude_cat2...
## dbl (35): Study_number, Year, Effect_size_id, Latitude_cont, Response_number...
## 
## ℹ Use `spec()` to retrieve the full column specification for this data.
## ℹ Specify the column types or set `show_col_types = FALSE` to quiet this message.
```

## Turning random effects into factors

```
Warmingratedata$Study_number = factor(Warmingratedata$Study_number)
Warmingratedata$Response_number = factor(Warmingratedata$Response_number)
```

## Ordering data

```
Warmingratedata <- Warmingratedata[order(Warmingratedata$Warming_rate),]
```

## The model

```
Warmingrate_model <- rma.mv(yi = LnRR, V = Variance, mods = Warming_rate-1, test="t", random = list(~1|Study_number/Response_number), method = "REML", data = Warmingratedata)
```

```
## Warning: 531 rows with NAs omitted from model fitting.
```

```
print(Warmingrate_model)
```

```
## 
## Multivariate Meta-Analysis Model (k = 127; method: REML)
## 
## Variance Components:
## 
##             estim    sqrt  nlvls  fixed                        factor 
## sigma^2.1  0.1976  0.4445     17     no                  Study_number 
## sigma^2.2  0.1012  0.3180     80     no  Study_number/Response_number 
## 
## Test for Residual Heterogeneity:
## QE(df = 125) = 3564.6259, p-val < .0001
## 
## Test of Moderators (coefficient 2):
## F(df1 = 1, df2 = 125) = 1.4686, p-val = 0.2279
## 
## Model Results:
## 
##          estimate      se    tval   df    pval    ci.lb   ci.ub    
## intrcpt    0.9319  0.8141  1.1447  125  0.2545  -0.6793  2.5431    
## mods       1.0438  0.8613  1.2118  125  0.2279  -0.6609  2.7485    
## 
## ---
## Signif. codes:  0 '***' 0.001 '**' 0.01 '*' 0.05 '.' 0.1 ' ' 1
```

## Robust model

```
Warmingrate_model_robust <- robust(Warmingrate_model, cluster=Warmingratedata$Study_number)
Warmingrate_model_robust
```

```
## 
## Multivariate Meta-Analysis Model (k = 127; method: REML)
## 
## Variance Components:
## 
##             estim    sqrt  nlvls  fixed                        factor 
## sigma^2.1  0.1976  0.4445     17     no                  Study_number 
## sigma^2.2  0.1012  0.3180     80     no  Study_number/Response_number 
## 
## Test for Residual Heterogeneity:
## QE(df = 125) = 3564.6259, p-val < .0001
## 
## Number of estimates:   127
## Number of clusters:    17
## Estimates per cluster: 2-36 (mean: 7.47, median: 5)
## 
## Test of Moderators (coefficient 2):¹
## F(df1 = 1, df2 = 15) = 10.4436, p-val = 0.0056
## 
## Model Results:
## 
##          estimate      se¹    tval¹  df¹    pval¹   ci.lb¹   ci.ub¹     
## intrcpt    0.9319  0.3275   2.8452   15   0.0123   0.2338   1.6300    * 
## mods       1.0438  0.3230   3.2317   15   0.0056   0.3554   1.7322   ** 
## 
## ---
## Signif. codes:  0 '***' 0.001 '**' 0.01 '*' 0.05 '.' 0.1 ' ' 1
## 
## 1) results based on cluster-robust inference (var-cov estimator: CR1,
##    approx t/F-tests and confidence intervals, df: residual method)
```

## Checking model fit

```
res <- resid(Warmingrate_model_robust)
plot(fitted(Warmingrate_model_robust), res)
abline(0,0)
```

```
qqnorm(res)
qqline(res)
```

```
plot(density(res))
```

# Models with observations removed where the experimental temperature is lower than the future (+2.58C) mean annual temperature (MAT)

## Loading data

```
setwd('/Users/bethanlang/Documents/PhD/Literature Review')
Warmingratedata_CC <- read_csv("Lang et al. 2022_dataset_CC2906_nometabolism.csv")
```

```
## Rows: 488 Columns: 44
## ── Column specification ────────────────────────────────────────────────────────
## Delimiter: ","
## chr  (9): Study_name, Species, Class, Lifestage, Latitude_cat, Latitude_cat2...
## dbl (35): Study_number, Year, Effect_size_id, Latitude_cont, Response_number...
## 
## ℹ Use `spec()` to retrieve the full column specification for this data.
## ℹ Specify the column types or set `show_col_types = FALSE` to quiet this message.
```

## Turning random effects into factors

```
Warmingratedata_CC$Study_number = factor(Warmingratedata_CC$Study_number)
Warmingratedata_CC$Response_number = factor(Warmingratedata_CC$Response_number)
```

## Ordering data

```
Warmingratedata_CC <- Warmingratedata_CC[order(Warmingratedata_CC$Warming_rate),]
```

## The model

```
Warmingrate_model_CC <- rma.mv(yi = LnRR, V = Variance, mods = ~Warming_rate-1, test="t", random = list(~1|Study_number/Response_number), method = "REML", data = Warmingratedata_CC)
```

```
## Warning: 378 rows with NAs omitted from model fitting.
```

```
print(Warmingrate_model_CC)
```

```
## 
## Multivariate Meta-Analysis Model (k = 110; method: REML)
## 
## Variance Components:
## 
##             estim    sqrt  nlvls  fixed                        factor 
## sigma^2.1  0.1880  0.4336     16     no                  Study_number 
## sigma^2.2  0.1050  0.3240     79     no  Study_number/Response_number 
## 
## Test for Residual Heterogeneity:
## QE(df = 109) = 3506.6950, p-val < .0001
## 
## Model Results:
## 
##               estimate      se    tval   df    pval    ci.lb   ci.ub    
## Warming_rate    0.6373  0.7780  0.8193  109  0.4144  -0.9045  2.1792    
## 
## ---
## Signif. codes:  0 '***' 0.001 '**' 0.01 '*' 0.05 '.' 0.1 ' ' 1
```

## Robust model

```
Warmingrate_model_robust_CC <- robust(Warmingrate_model_CC, cluster=Warmingratedata_CC$Study_number)
Warmingrate_model_robust_CC
```

```
## 
## Multivariate Meta-Analysis Model (k = 110; method: REML)
## 
## Variance Components:
## 
##             estim    sqrt  nlvls  fixed                        factor 
## sigma^2.1  0.1880  0.4336     16     no                  Study_number 
## sigma^2.2  0.1050  0.3240     79     no  Study_number/Response_number 
## 
## Test for Residual Heterogeneity:
## QE(df = 109) = 3506.6950, p-val < .0001
## 
## Number of estimates:   110
## Number of clusters:    16
## Estimates per cluster: 2-27 (mean: 6.88, median: 5)
## 
## Model Results:
## 
##               estimate      se¹    tval¹  df¹    pval¹    ci.lb¹   ci.ub¹    
## Warming_rate    0.6373  0.6474   0.9845   15   0.3405   -0.7425   2.0172     
## 
## ---
## Signif. codes:  0 '***' 0.001 '**' 0.01 '*' 0.05 '.' 0.1 ' ' 1
## 
## 1) results based on cluster-robust inference (var-cov estimator: CR1,
##    approx t/F-tests and confidence intervals, df: residual method)
```

# Exposure time (all data)

## Loading data

```
setwd('/Users/bethanlang/Documents/PhD/Literature Review')
Exposuretimedata <- read_csv("Lang et al. 2022_dataset_2906_nometabolism.csv")
```

```
## Rows: 658 Columns: 44
## ── Column specification ────────────────────────────────────────────────────────
## Delimiter: ","
## chr  (9): Study_name, Species, Class, Lifestage, Latitude_cat, Latitude_cat2...
## dbl (35): Study_number, Year, Effect_size_id, Latitude_cont, Response_number...
## 
## ℹ Use `spec()` to retrieve the full column specification for this data.
## ℹ Specify the column types or set `show_col_types = FALSE` to quiet this message.
```

## Turning random effects into factors

```
Exposuretimedata$Study_number = factor(Exposuretimedata$Study_number)
Exposuretimedata$Response_number = factor(Exposuretimedata$Response_number)
```

## Ordering data

```
Exposuretimedata <- Exposuretimedata[order(Exposuretimedata$Exposure_time),]
```

## The model

```
Exposuretime_model <- rma.mv(yi = LnRR, V = Variance, mods = ~Exposure_time-1, test="t", random = list(~1|Study_number/Response_number), method = "REML", data = Exposuretimedata)
```

```
## Warning: 391 rows with NAs omitted from model fitting.
```

```
print(Exposuretime_model)
```

```
## 
## Multivariate Meta-Analysis Model (k = 267; method: REML)
## 
## Variance Components:
## 
##             estim    sqrt  nlvls  fixed                        factor 
## sigma^2.1  0.0802  0.2832     34     no                  Study_number 
## sigma^2.2  0.1004  0.3169    182     no  Study_number/Response_number 
## 
## Test for Residual Heterogeneity:
## QE(df = 266) = 6559.8797, p-val < .0001
## 
## Model Results:
## 
##                estimate      se    tval   df    pval    ci.lb   ci.ub    
## Exposure_time    0.0001  0.0002  0.6397  266  0.5229  -0.0002  0.0005    
## 
## ---
## Signif. codes:  0 '***' 0.001 '**' 0.01 '*' 0.05 '.' 0.1 ' ' 1
```

## Robust model

```
Exposuretime_model_robust <- robust(Exposuretime_model, cluster=Exposuretimedata$Study_number)
Exposuretime_model_robust
```

```
## 
## Multivariate Meta-Analysis Model (k = 267; method: REML)
## 
## Variance Components:
## 
##             estim    sqrt  nlvls  fixed                        factor 
## sigma^2.1  0.0802  0.2832     34     no                  Study_number 
## sigma^2.2  0.1004  0.3169    182     no  Study_number/Response_number 
## 
## Test for Residual Heterogeneity:
## QE(df = 266) = 6559.8797, p-val < .0001
## 
## Number of estimates:   267
## Number of clusters:    34
## Estimates per cluster: 2-30 (mean: 7.85, median: 5)
## 
## Model Results:
## 
##                estimate      se¹    tval¹  df¹    pval¹    ci.lb¹   ci.ub¹    
## Exposure_time    0.0001  0.0002   0.6543   33   0.5175   -0.0002   0.0005     
## 
## ---
## Signif. codes:  0 '***' 0.001 '**' 0.01 '*' 0.05 '.' 0.1 ' ' 1
## 
## 1) results based on cluster-robust inference (var-cov estimator: CR1,
##    approx t/F-tests and confidence intervals, df: residual method)
```

## Checking model fit

```
res <- resid(Exposuretime_model_robust)
plot(fitted(Exposuretime_model_robust), res)
abline(0,0)
```

```
qqnorm(res)
qqline(res)
```

```
plot(density(res))
```

# Models with observations removed where the experimental temperature is lower than the future (+2.58C) mean annual temperature (MAT)

## Loading data

```
setwd('/Users/bethanlang/Documents/PhD/Literature Review')
Exposuretimedata_CC <- read_csv("Lang et al. 2022_dataset_CC2906_nometabolism.csv")
```

```
## Rows: 488 Columns: 44
## ── Column specification ────────────────────────────────────────────────────────
## Delimiter: ","
## chr  (9): Study_name, Species, Class, Lifestage, Latitude_cat, Latitude_cat2...
## dbl (35): Study_number, Year, Effect_size_id, Latitude_cont, Response_number...
## 
## ℹ Use `spec()` to retrieve the full column specification for this data.
## ℹ Specify the column types or set `show_col_types = FALSE` to quiet this message.
```

## Turning random effects into factors

```
Exposuretimedata_CC$Study_number = factor(Exposuretimedata_CC$Study_number)
Exposuretimedata_CC$Response_number = factor(Exposuretimedata_CC$Response_number)
```

## Ordering data

```
Exposuretimedata_CC <- Exposuretimedata_CC[order(Exposuretimedata_CC$Exposure_time),]
```

## The model

```
Exposuretime_model_CC <- rma.mv(yi = LnRR, V = Variance, mods = ~Exposure_time-1, test="t", random = list(~1|Study_number/Response_number), method = "REML", data = Exposuretimedata_CC)
```

```
## Warning: 263 rows with NAs omitted from model fitting.
```

```
print(Exposuretime_model_CC)
```

```
## 
## Multivariate Meta-Analysis Model (k = 225; method: REML)
## 
## Variance Components:
## 
##             estim    sqrt  nlvls  fixed                        factor 
## sigma^2.1  0.0901  0.3001     31     no                  Study_number 
## sigma^2.2  0.1005  0.3170    172     no  Study_number/Response_number 
## 
## Test for Residual Heterogeneity:
## QE(df = 224) = 6024.4399, p-val < .0001
## 
## Model Results:
## 
##                estimate      se    tval   df    pval    ci.lb   ci.ub    
## Exposure_time    0.0001  0.0003  0.5511  224  0.5821  -0.0004  0.0006    
## 
## ---
## Signif. codes:  0 '***' 0.001 '**' 0.01 '*' 0.05 '.' 0.1 ' ' 1
```

## Robust model

```
Exposuretime_model_robust_CC <- robust(Exposuretime_model_CC, cluster=Exposuretimedata_CC$Study_number)
Exposuretime_model_robust_CC
```

```
## 
## Multivariate Meta-Analysis Model (k = 225; method: REML)
## 
## Variance Components:
## 
##             estim    sqrt  nlvls  fixed                        factor 
## sigma^2.1  0.0901  0.3001     31     no                  Study_number 
## sigma^2.2  0.1005  0.3170    172     no  Study_number/Response_number 
## 
## Test for Residual Heterogeneity:
## QE(df = 224) = 6024.4399, p-val < .0001
## 
## Number of estimates:   225
## Number of clusters:    31
## Estimates per cluster: 1-30 (mean: 7.26, median: 5)
## 
## Model Results:
## 
##                estimate      se¹    tval¹  df¹    pval¹    ci.lb¹   ci.ub¹    
## Exposure_time    0.0001  0.0003   0.4135   30   0.6822   -0.0006   0.0008     
## 
## ---
## Signif. codes:  0 '***' 0.001 '**' 0.01 '*' 0.05 '.' 0.1 ' ' 1
## 
## 1) results based on cluster-robust inference (var-cov estimator: CR1,
##    approx t/F-tests and confidence intervals, df: residual method)
```

# Models without Acanthaster spp.

## Class

```
setwd('/Users/bethanlang/Documents/PhD/Literature Review')
Classdata <- read_csv("Lang et al. 2022_dataset_2909_noCoTS_nometabolism.csv")
```

```
## New names:
## Rows: 548 Columns: 50
## ── Column specification
## ──────────────────────────────────────────────────────── Delimiter: "," chr
## (9): Study_name, Species, Class, Lifestage, Latitude_cat, Latitude_cat2... dbl
## (40): Study_number, Year, Effect_size_id, lat...8, Response_number, Ctl_... lgl
## (1): Exp_temp_between_CC_MAT_WARM
## ℹ Use `spec()` to retrieve the full column specification for this data. ℹ
## Specify the column types or set `show_col_types = FALSE` to quiet this message.
## • `lat` -> `lat...8`
## • `Exp_temp` -> `Exp_temp...19`
## • `lat` -> `lat...35`
## • `Exp_temp` -> `Exp_temp...42`
```

## Turning random effects into factors

```
Classdata$Study_number = factor(Classdata$Study_number)
Classdata$Response_number = factor(Classdata$Response_number)
```

## Ordering data

```
Classdata <- Classdata[order(Classdata$Class),]
```

## The model

```
Class_model <- rma.mv(yi = LnRR, V = Variance, mods = ~LnSR:Class-1, test="t", random = list(~1|Study_number/Response_number), method = "REML", data = Classdata)
print(Class_model)
```

```
## 
## Multivariate Meta-Analysis Model (k = 548; method: REML)
## 
## Variance Components:
## 
##             estim    sqrt  nlvls  fixed                        factor 
## sigma^2.1  0.1160  0.3407     72     no                  Study_number 
## sigma^2.2  0.0968  0.3111    312     no  Study_number/Response_number 
## 
## Test for Residual Heterogeneity:
## QE(df = 544) = 21860.4678, p-val < .0001
## 
## Test of Moderators (coefficients 1:4):
## F(df1 = 4, df2 = 544) = 58.0422, p-val < .0001
## 
## Model Results:
## 
##                          estimate      se      tval   df    pval    ci.lb 
## LnSR:ClassAsteroidea      -0.3668  0.0284  -12.9109  544  <.0001  -0.4226 
## LnSR:ClassEchinoidea      -0.0066  0.0061   -1.0845  544  0.2786  -0.0186 
## LnSR:ClassHolothuroidea   -0.1101  0.0138   -7.9638  544  <.0001  -0.1372 
## LnSR:ClassOphiuroidea      0.1360  0.1450    0.9378  544  0.3488  -0.1489 
##                            ci.ub      
## LnSR:ClassAsteroidea     -0.3110  *** 
## LnSR:ClassEchinoidea      0.0054      
## LnSR:ClassHolothuroidea  -0.0829  *** 
## LnSR:ClassOphiuroidea     0.4209      
## 
## ---
## Signif. codes:  0 '***' 0.001 '**' 0.01 '*' 0.05 '.' 0.1 ' ' 1
```

## Robust model

```
Class_model_robust <- robust(Class_model, cluster=Classdata$Study_number)
Class_model_robust
```

```
## 
## Multivariate Meta-Analysis Model (k = 548; method: REML)
## 
## Variance Components:
## 
##             estim    sqrt  nlvls  fixed                        factor 
## sigma^2.1  0.1160  0.3407     72     no                  Study_number 
## sigma^2.2  0.0968  0.3111    312     no  Study_number/Response_number 
## 
## Test for Residual Heterogeneity:
## QE(df = 544) = 21860.4678, p-val < .0001
## 
## Number of estimates:   548
## Number of clusters:    72
## Estimates per cluster: 1-36 (mean: 7.61, median: 6)
## 
## Test of Moderators (coefficients 1:4):¹
## F(df1 = 4, df2 = 68) = 5.8830, p-val = 0.0004
## 
## Model Results:
## 
##                          estimate      se¹     tval¹  df¹    pval¹    ci.lb¹ 
## LnSR:ClassAsteroidea      -0.3668  0.1609   -2.2794   68   0.0258   -0.6880  
## LnSR:ClassEchinoidea      -0.0066  0.0141   -0.4705   68   0.6395   -0.0348  
## LnSR:ClassHolothuroidea   -0.1101  0.0269   -4.0968   68   0.0001   -0.1637  
## LnSR:ClassOphiuroidea      0.1360  0.1179    1.1537   68   0.2527   -0.0993  
##                            ci.ub¹      
## LnSR:ClassAsteroidea     -0.0457     * 
## LnSR:ClassEchinoidea      0.0215       
## LnSR:ClassHolothuroidea  -0.0565   *** 
## LnSR:ClassOphiuroidea     0.3713       
## 
## ---
## Signif. codes:  0 '***' 0.001 '**' 0.01 '*' 0.05 '.' 0.1 ' ' 1
## 
## 1) results based on cluster-robust inference (var-cov estimator: CR1,
##    approx t/F-tests and confidence intervals, df: residual method)
```

## Latitude

```
setwd('/Users/bethanlang/Documents/PhD/Literature Review')
Latitudedata <- read_csv("Lang et al. 2022_dataset_2909_noCoTS_nometabolism.csv")
```

```
## New names:
## Rows: 548 Columns: 50
## ── Column specification
## ──────────────────────────────────────────────────────── Delimiter: "," chr
## (9): Study_name, Species, Class, Lifestage, Latitude_cat, Latitude_cat2... dbl
## (40): Study_number, Year, Effect_size_id, lat...8, Response_number, Ctl_... lgl
## (1): Exp_temp_between_CC_MAT_WARM
## ℹ Use `spec()` to retrieve the full column specification for this data. ℹ
## Specify the column types or set `show_col_types = FALSE` to quiet this message.
## • `lat` -> `lat...8`
## • `Exp_temp` -> `Exp_temp...19`
## • `lat` -> `lat...35`
## • `Exp_temp` -> `Exp_temp...42`
```

## Turning random effects into factors

```
Latitudedata$Study_number = factor(Latitudedata$Study_number)
Latitudedata$Response_number = factor(Latitudedata$Response_number)
```

## Ordering data

```
Latitudedata <- Latitudedata[order(Latitudedata$Latitude_cat2),]
```

## The model

```
Latitude_model <- rma.mv(yi = LnRR, V = Variance, mods = ~LnSR:Latitude_cat2-1,  test="t", random = list(~1|Study_number/Response_number), method = "REML", data = Latitudedata)
print(Latitude_model)
```

```
## 
## Multivariate Meta-Analysis Model (k = 548; method: REML)
## 
## Variance Components:
## 
##             estim    sqrt  nlvls  fixed                        factor 
## sigma^2.1  0.1231  0.3508     72     no                  Study_number 
## sigma^2.2  0.0958  0.3095    312     no  Study_number/Response_number 
## 
## Test for Residual Heterogeneity:
## QE(df = 544) = 20600.4865, p-val < .0001
## 
## Test of Moderators (coefficients 1:4):
## F(df1 = 4, df2 = 544) = 21.9394, p-val < .0001
## 
## Model Results:
## 
##                                          estimate      se     tval   df    pval 
## LnSR:Latitude_cat2Polar (62-78°)           0.0308  0.0139   2.2179  544  0.0270 
## LnSR:Latitude_cat2Sub-tropical (26-35°)   -0.0927  0.0142  -6.5225  544  <.0001 
## LnSR:Latitude_cat2Temperate (36-53°)      -0.0589  0.0110  -5.3556  544  <.0001 
## LnSR:Latitude_cat2Tropical (4-22°)        -0.0280  0.0082  -3.4077  544  0.0007 
##                                            ci.lb    ci.ub      
## LnSR:Latitude_cat2Polar (62-78°)          0.0035   0.0580    * 
## LnSR:Latitude_cat2Sub-tropical (26-35°)  -0.1206  -0.0648  *** 
## LnSR:Latitude_cat2Temperate (36-53°)     -0.0806  -0.0373  *** 
## LnSR:Latitude_cat2Tropical (4-22°)       -0.0441  -0.0118  *** 
## 
## ---
## Signif. codes:  0 '***' 0.001 '**' 0.01 '*' 0.05 '.' 0.1 ' ' 1
```

## Robust model

```
Latitude_model_robust <- robust(Latitude_model, cluster=Latitudedata$Study_number)
Latitude_model_robust
```

```
## 
## Multivariate Meta-Analysis Model (k = 548; method: REML)
## 
## Variance Components:
## 
##             estim    sqrt  nlvls  fixed                        factor 
## sigma^2.1  0.1231  0.3508     72     no                  Study_number 
## sigma^2.2  0.0958  0.3095    312     no  Study_number/Response_number 
## 
## Test for Residual Heterogeneity:
## QE(df = 544) = 20600.4865, p-val < .0001
## 
## Number of estimates:   548
## Number of clusters:    72
## Estimates per cluster: 1-36 (mean: 7.61, median: 6)
## 
## Test of Moderators (coefficients 1:4):¹
## F(df1 = 4, df2 = 68) = 3.4400, p-val = 0.0128
## 
## Model Results:
## 
##                                          estimate      se¹     tval¹  df¹ 
## LnSR:Latitude_cat2Polar (62-78°)           0.0308  0.0331    0.9312   68  
## LnSR:Latitude_cat2Sub-tropical (26-35°)   -0.0927  0.0391   -2.3725   68  
## LnSR:Latitude_cat2Temperate (36-53°)      -0.0589  0.0348   -1.6926   68  
## LnSR:Latitude_cat2Tropical (4-22°)        -0.0280  0.0133   -2.0974   68  
##                                            pval¹    ci.lb¹    ci.ub¹    
## LnSR:Latitude_cat2Polar (62-78°)         0.3550   -0.0352    0.0967     
## LnSR:Latitude_cat2Sub-tropical (26-35°)  0.0205   -0.1707   -0.0147   * 
## LnSR:Latitude_cat2Temperate (36-53°)     0.0951   -0.1284    0.0105   . 
## LnSR:Latitude_cat2Tropical (4-22°)       0.0397   -0.0546   -0.0014   * 
## 
## ---
## Signif. codes:  0 '***' 0.001 '**' 0.01 '*' 0.05 '.' 0.1 ' ' 1
## 
## 1) results based on cluster-robust inference (var-cov estimator: CR1,
##    approx t/F-tests and confidence intervals, df: residual method)
```

# Models without Strongylocentrotus droebachiensis

## Class

```
setwd('/Users/bethanlang/Documents/PhD/Literature Review')
Classdata <- read_csv("Lang et al. 2022_dataset_2906_nometabolism_noSD.csv")
```

```
## Rows: 605 Columns: 44
## ── Column specification ────────────────────────────────────────────────────────
## Delimiter: ","
## chr  (9): Study_name, Species, Class, Lifestage, Latitude_cat, Latitude_cat2...
## dbl (35): Study_number, Year, Effect_size_id, Latitude_cont, Response_number...
## 
## ℹ Use `spec()` to retrieve the full column specification for this data.
## ℹ Specify the column types or set `show_col_types = FALSE` to quiet this message.
```

## Turning random effects into factors

```
Classdata$Study_number = factor(Classdata$Study_number)
Classdata$Response_number = factor(Classdata$Response_number)
```

## Ordering data

```
Classdata <- Classdata[order(Classdata$Class),]
```

## The model

```
Class_model <- rma.mv(yi = LnRR, V = Variance, mods = ~LnSR:Class-1, test="t", random = list(~1|Study_number/Response_number), method = "REML", data = Classdata)
print(Class_model)
```

```
## 
## Multivariate Meta-Analysis Model (k = 605; method: REML)
## 
## Variance Components:
## 
##             estim    sqrt  nlvls  fixed                        factor 
## sigma^2.1  0.1208  0.3475     77     no                  Study_number 
## sigma^2.2  0.0925  0.3041    343     no  Study_number/Response_number 
## 
## Test for Residual Heterogeneity:
## QE(df = 601) = 22860.2718, p-val < .0001
## 
## Test of Moderators (coefficients 1:4):
## F(df1 = 4, df2 = 601) = 144.1071, p-val < .0001
## 
## Model Results:
## 
##                          estimate      se      tval   df    pval    ci.lb 
## LnSR:ClassAsteroidea      -0.3522  0.0157  -22.4794  601  <.0001  -0.3829 
## LnSR:ClassEchinoidea      -0.0169  0.0065   -2.6047  601  0.0094  -0.0297 
## LnSR:ClassHolothuroidea   -0.1101  0.0138   -7.9663  601  <.0001  -0.1373 
## LnSR:ClassOphiuroidea      0.1361  0.1467    0.9280  601  0.3538  -0.1519 
##                            ci.ub      
## LnSR:ClassAsteroidea     -0.3214  *** 
## LnSR:ClassEchinoidea     -0.0042   ** 
## LnSR:ClassHolothuroidea  -0.0830  *** 
## LnSR:ClassOphiuroidea     0.4241      
## 
## ---
## Signif. codes:  0 '***' 0.001 '**' 0.01 '*' 0.05 '.' 0.1 ' ' 1
```

## Robust model

```
Class_model_robust <- robust(Class_model, cluster=Classdata$Study_number)
Class_model_robust
```

```
## 
## Multivariate Meta-Analysis Model (k = 605; method: REML)
## 
## Variance Components:
## 
##             estim    sqrt  nlvls  fixed                        factor 
## sigma^2.1  0.1208  0.3475     77     no                  Study_number 
## sigma^2.2  0.0925  0.3041    343     no  Study_number/Response_number 
## 
## Test for Residual Heterogeneity:
## QE(df = 601) = 22860.2718, p-val < .0001
## 
## Number of estimates:   605
## Number of clusters:    77
## Estimates per cluster: 1-36 (mean: 7.86, median: 6)
## 
## Test of Moderators (coefficients 1:4):¹
## F(df1 = 4, df2 = 73) = 15.3235, p-val < .0001
## 
## Model Results:
## 
##                          estimate      se¹     tval¹  df¹    pval¹    ci.lb¹ 
## LnSR:ClassAsteroidea      -0.3522  0.0554   -6.3601   73   <.0001   -0.4625  
## LnSR:ClassEchinoidea      -0.0169  0.0105   -1.6190   73   0.1098   -0.0378  
## LnSR:ClassHolothuroidea   -0.1101  0.0268   -4.1072   73   0.0001   -0.1636  
## LnSR:ClassOphiuroidea      0.1361  0.1170    1.1630   73   0.2486   -0.0971  
##                            ci.ub¹      
## LnSR:ClassAsteroidea     -0.2418   *** 
## LnSR:ClassEchinoidea      0.0039       
## LnSR:ClassHolothuroidea  -0.0567   *** 
## LnSR:ClassOphiuroidea     0.3693       
## 
## ---
## Signif. codes:  0 '***' 0.001 '**' 0.01 '*' 0.05 '.' 0.1 ' ' 1
## 
## 1) results based on cluster-robust inference (var-cov estimator: CR1,
##    approx t/F-tests and confidence intervals, df: residual method)
```

## Latitude

```
setwd('/Users/bethanlang/Documents/PhD/Literature Review')
Latitudedata <- read_csv("Lang et al. 2022_dataset_2906_nometabolism_noSD.csv")
```

```
## Rows: 605 Columns: 44
## ── Column specification ────────────────────────────────────────────────────────
## Delimiter: ","
## chr  (9): Study_name, Species, Class, Lifestage, Latitude_cat, Latitude_cat2...
## dbl (35): Study_number, Year, Effect_size_id, Latitude_cont, Response_number...
## 
## ℹ Use `spec()` to retrieve the full column specification for this data.
## ℹ Specify the column types or set `show_col_types = FALSE` to quiet this message.
```

## Turning random effects into factors

```
Latitudedata$Study_number = factor(Latitudedata$Study_number)
Latitudedata$Response_number = factor(Latitudedata$Response_number)
```

## Ordering data

```
Latitudedata <- Latitudedata[order(Latitudedata$Latitude_cat2),]
```

## The model

```
Latitude_model <- rma.mv(yi = LnRR, V = Variance, mods = ~LnSR:Latitude_cat2-1,  test="t", random = list(~1|Study_number/Response_number), method = "REML", data = Latitudedata)
print(Latitude_model)
```

```
## 
## Multivariate Meta-Analysis Model (k = 605; method: REML)
## 
## Variance Components:
## 
##             estim    sqrt  nlvls  fixed                        factor 
## sigma^2.1  0.1187  0.3445     77     no                  Study_number 
## sigma^2.2  0.0919  0.3031    343     no  Study_number/Response_number 
## 
## Test for Residual Heterogeneity:
## QE(df = 601) = 23829.7337, p-val < .0001
## 
## Test of Moderators (coefficients 1:4):
## F(df1 = 4, df2 = 601) = 45.8381, p-val < .0001
## 
## Model Results:
## 
##                                             estimate      se      tval   df 
## LnSR:Latitude_cat2a. Polar (62-78°)          -0.0310  0.0218   -1.4237  601 
## LnSR:Latitude_cat2b. Temperate (36-53°)      -0.0588  0.0110   -5.3362  601 
## LnSR:Latitude_cat2c. Sub-tropical (26-35°)   -0.0927  0.0142   -6.5244  601 
## LnSR:Latitude_cat2d. Tropical (4-23°)        -0.0790  0.0075  -10.5015  601 
##                                               pval    ci.lb    ci.ub      
## LnSR:Latitude_cat2a. Polar (62-78°)         0.1551  -0.0738   0.0118      
## LnSR:Latitude_cat2b. Temperate (36-53°)     <.0001  -0.0804  -0.0372  *** 
## LnSR:Latitude_cat2c. Sub-tropical (26-35°)  <.0001  -0.1206  -0.0648  *** 
## LnSR:Latitude_cat2d. Tropical (4-23°)       <.0001  -0.0937  -0.0642  *** 
## 
## ---
## Signif. codes:  0 '***' 0.001 '**' 0.01 '*' 0.05 '.' 0.1 ' ' 1
```

## Robust model

```
Latitude_model_robust <- robust(Latitude_model, cluster=Latitudedata$Study_number)
Latitude_model_robust
```

```
## 
## Multivariate Meta-Analysis Model (k = 605; method: REML)
## 
## Variance Components:
## 
##             estim    sqrt  nlvls  fixed                        factor 
## sigma^2.1  0.1187  0.3445     77     no                  Study_number 
## sigma^2.2  0.0919  0.3031    343     no  Study_number/Response_number 
## 
## Test for Residual Heterogeneity:
## QE(df = 601) = 23829.7337, p-val < .0001
## 
## Number of estimates:   605
## Number of clusters:    77
## Estimates per cluster: 1-36 (mean: 7.86, median: 6)
## 
## Test of Moderators (coefficients 1:4):¹
## F(df1 = 4, df2 = 73) = 2.6924, p-val = 0.0375
## 
## Model Results:
## 
##                                             estimate      se¹     tval¹  df¹ 
## LnSR:Latitude_cat2a. Polar (62-78°)          -0.0310  0.0520   -0.5964   73  
## LnSR:Latitude_cat2b. Temperate (36-53°)      -0.0588  0.0349   -1.6857   73  
## LnSR:Latitude_cat2c. Sub-tropical (26-35°)   -0.0927  0.0389   -2.3800   73  
## LnSR:Latitude_cat2d. Tropical (4-23°)        -0.0790  0.0572   -1.3812   73  
##                                               pval¹    ci.lb¹    ci.ub¹    
## LnSR:Latitude_cat2a. Polar (62-78°)         0.5528   -0.1346    0.0726     
## LnSR:Latitude_cat2b. Temperate (36-53°)     0.0961   -0.1283    0.0107   . 
## LnSR:Latitude_cat2c. Sub-tropical (26-35°)  0.0199   -0.1703   -0.0151   * 
## LnSR:Latitude_cat2d. Tropical (4-23°)       0.1714   -0.1929    0.0350     
## 
## ---
## Signif. codes:  0 '***' 0.001 '**' 0.01 '*' 0.05 '.' 0.1 ' ' 1
## 
## 1) results based on cluster-robust inference (var-cov estimator: CR1,
##    approx t/F-tests and confidence intervals, df: residual method)
```

# Models without Apostichopus japonicus

## Class

```
setwd('/Users/bethanlang/Documents/PhD/Literature Review')
Classdata <- read_csv("Lang et al. 2022_dataset_2909_nometabolism_noAJ.csv")
```

```
## Rows: 592 Columns: 44
## ── Column specification ────────────────────────────────────────────────────────
## Delimiter: ","
## chr  (9): Study_name, Species, Class, Lifestage, Latitude_cat, Latitude_cat2...
## dbl (35): Study_number, Year, Effect_size_id, Latitude_cont, Response_number...
## 
## ℹ Use `spec()` to retrieve the full column specification for this data.
## ℹ Specify the column types or set `show_col_types = FALSE` to quiet this message.
```

## Turning random effects into factors

```
Classdata$Study_number = factor(Classdata$Study_number)
Classdata$Response_number = factor(Classdata$Response_number)
```

## Ordering data

```
Classdata <- Classdata[order(Classdata$Class),]
```

## The model

```
Class_model <- rma.mv(yi = LnRR, V = Variance, mods = ~LnSR:Class-1, test="t", random = list(~1|Study_number/Response_number), method = "REML", data = Classdata)
print(Class_model)
```

```
## 
## Multivariate Meta-Analysis Model (k = 592; method: REML)
## 
## Variance Components:
## 
##             estim    sqrt  nlvls  fixed                        factor 
## sigma^2.1  0.2373  0.4871     75     no                  Study_number 
## sigma^2.2  0.0966  0.3108    339     no  Study_number/Response_number 
## 
## Test for Residual Heterogeneity:
## QE(df = 588) = 22001.8440, p-val < .0001
## 
## Test of Moderators (coefficients 1:4):
## F(df1 = 4, df2 = 588) = 151.1485, p-val < .0001
## 
## Model Results:
## 
##                          estimate      se      tval   df    pval    ci.lb 
## LnSR:ClassAsteroidea      -0.3549  0.0158  -22.4656  588  <.0001  -0.3860 
## LnSR:ClassEchinoidea      -0.0067  0.0061   -1.0918  588  0.2754  -0.0187 
## LnSR:ClassHolothuroidea   -1.0628  0.1073   -9.9092  588  <.0001  -1.2735 
## LnSR:ClassOphiuroidea      0.1331  0.1876    0.7096  588  0.4782  -0.2353 
##                            ci.ub      
## LnSR:ClassAsteroidea     -0.3239  *** 
## LnSR:ClassEchinoidea      0.0053      
## LnSR:ClassHolothuroidea  -0.8522  *** 
## LnSR:ClassOphiuroidea     0.5015      
## 
## ---
## Signif. codes:  0 '***' 0.001 '**' 0.01 '*' 0.05 '.' 0.1 ' ' 1
```

## Robust model

```
Class_model_robust <- robust(Class_model, cluster=Classdata$Study_number)
Class_model_robust
```

```
## 
## Multivariate Meta-Analysis Model (k = 592; method: REML)
## 
## Variance Components:
## 
##             estim    sqrt  nlvls  fixed                        factor 
## sigma^2.1  0.2373  0.4871     75     no                  Study_number 
## sigma^2.2  0.0966  0.3108    339     no  Study_number/Response_number 
## 
## Test for Residual Heterogeneity:
## QE(df = 588) = 22001.8440, p-val < .0001
## 
## Number of estimates:   592
## Number of clusters:    75
## Estimates per cluster: 1-30 (mean: 7.89, median: 6)
## 
## Test of Moderators (coefficients 1:4):¹
## F(df1 = 4, df2 = 71) = 11.6844, p-val < .0001
## 
## Model Results:
## 
##                          estimate      se¹     tval¹  df¹    pval¹    ci.lb¹ 
## LnSR:ClassAsteroidea      -0.3549  0.0557   -6.3776   71   <.0001   -0.4659  
## LnSR:ClassEchinoidea      -0.0067  0.0141   -0.4758   71   0.6357   -0.0348  
## LnSR:ClassHolothuroidea   -1.0628  0.5004   -2.1241   71   0.0371   -2.0605  
## LnSR:ClassOphiuroidea      0.1331  0.1156    1.1512   71   0.2535   -0.0974  
##                            ci.ub¹      
## LnSR:ClassAsteroidea     -0.2440   *** 
## LnSR:ClassEchinoidea      0.0214       
## LnSR:ClassHolothuroidea  -0.0651     * 
## LnSR:ClassOphiuroidea     0.3636       
## 
## ---
## Signif. codes:  0 '***' 0.001 '**' 0.01 '*' 0.05 '.' 0.1 ' ' 1
## 
## 1) results based on cluster-robust inference (var-cov estimator: CR1,
##    approx t/F-tests and confidence intervals, df: residual method)
```

## Latitude

```
setwd('/Users/bethanlang/Documents/PhD/Literature Review')
Latitudedata <- read_csv("Lang et al. 2022_dataset_2909_nometabolism_noAJ.csv")
```

```
## Rows: 592 Columns: 44
## ── Column specification ────────────────────────────────────────────────────────
## Delimiter: ","
## chr  (9): Study_name, Species, Class, Lifestage, Latitude_cat, Latitude_cat2...
## dbl (35): Study_number, Year, Effect_size_id, Latitude_cont, Response_number...
## 
## ℹ Use `spec()` to retrieve the full column specification for this data.
## ℹ Specify the column types or set `show_col_types = FALSE` to quiet this message.
```

## Turning random effects into factors

```
Latitudedata$Study_number = factor(Latitudedata$Study_number)
Latitudedata$Response_number = factor(Latitudedata$Response_number)
```

## Ordering data

```
Latitudedata <- Latitudedata[order(Latitudedata$Latitude_cat2),]
```

## The model

```
Latitude_model <- rma.mv(yi = LnRR, V = Variance, mods = ~LnSR:Latitude_cat2-1,  test="t", random = list(~1|Study_number/Response_number), method = "REML", data = Latitudedata)
print(Latitude_model)
```

```
## 
## Multivariate Meta-Analysis Model (k = 592; method: REML)
## 
## Variance Components:
## 
##             estim    sqrt  nlvls  fixed                        factor 
## sigma^2.1  0.1146  0.3386     75     no                  Study_number 
## sigma^2.2  0.0956  0.3092    339     no  Study_number/Response_number 
## 
## Test for Residual Heterogeneity:
## QE(df = 588) = 22907.7958, p-val < .0001
## 
## Test of Moderators (coefficients 1:4):
## F(df1 = 4, df2 = 588) = 39.4437, p-val < .0001
## 
## Model Results:
## 
##                                             estimate      se      tval   df 
## LnSR:Latitude_cat2a. Polar (62-78°)           0.0308  0.0139    2.2209  588 
## LnSR:Latitude_cat2b. Temperate (36-53°)      -0.0012  0.0178   -0.0661  588 
## LnSR:Latitude_cat2c. Sub-tropical (26-35°)   -0.0926  0.0142   -6.5250  588 
## LnSR:Latitude_cat2d. Tropical (4-23°)        -0.0790  0.0075  -10.5006  588 
##                                               pval    ci.lb    ci.ub      
## LnSR:Latitude_cat2a. Polar (62-78°)         0.0267   0.0036   0.0581    * 
## LnSR:Latitude_cat2b. Temperate (36-53°)     0.9473  -0.0362   0.0338      
## LnSR:Latitude_cat2c. Sub-tropical (26-35°)  <.0001  -0.1205  -0.0648  *** 
## LnSR:Latitude_cat2d. Tropical (4-23°)       <.0001  -0.0937  -0.0642  *** 
## 
## ---
## Signif. codes:  0 '***' 0.001 '**' 0.01 '*' 0.05 '.' 0.1 ' ' 1
```

## Robust model

```
Latitude_model_robust <- robust(Latitude_model, cluster=Latitudedata$Study_number)
Latitude_model_robust
```

```
## 
## Multivariate Meta-Analysis Model (k = 592; method: REML)
## 
## Variance Components:
## 
##             estim    sqrt  nlvls  fixed                        factor 
## sigma^2.1  0.1146  0.3386     75     no                  Study_number 
## sigma^2.2  0.0956  0.3092    339     no  Study_number/Response_number 
## 
## Test for Residual Heterogeneity:
## QE(df = 588) = 22907.7958, p-val < .0001
## 
## Number of estimates:   592
## Number of clusters:    75
## Estimates per cluster: 1-30 (mean: 7.89, median: 6)
## 
## Test of Moderators (coefficients 1:4):¹
## F(df1 = 4, df2 = 71) = 2.1102, p-val = 0.0885
## 
## Model Results:
## 
##                                             estimate      se¹     tval¹  df¹ 
## LnSR:Latitude_cat2a. Polar (62-78°)           0.0308  0.0330    0.9334   71  
## LnSR:Latitude_cat2b. Temperate (36-53°)      -0.0012  0.0442   -0.0267   71  
## LnSR:Latitude_cat2c. Sub-tropical (26-35°)   -0.0926  0.0389   -2.3798   71  
## LnSR:Latitude_cat2d. Tropical (4-23°)        -0.0790  0.0572   -1.3803   71  
##                                               pval¹    ci.lb¹    ci.ub¹    
## LnSR:Latitude_cat2a. Polar (62-78°)         0.3538   -0.0350    0.0966     
## LnSR:Latitude_cat2b. Temperate (36-53°)     0.9788   -0.0893    0.0869     
## LnSR:Latitude_cat2c. Sub-tropical (26-35°)  0.0200   -0.1703   -0.0150   * 
## LnSR:Latitude_cat2d. Tropical (4-23°)       0.1718   -0.1930    0.0351     
## 
## ---
## Signif. codes:  0 '***' 0.001 '**' 0.01 '*' 0.05 '.' 0.1 ' ' 1
## 
## 1) results based on cluster-robust inference (var-cov estimator: CR1,
##    approx t/F-tests and confidence intervals, df: residual method)
```
